# Controlling the LCST-Phase Transition in Azobenzene-Functionalized Poly (*N*-Isopropylacrlyamide) Hydrogels by Light

**DOI:** 10.3390/gels9020075

**Published:** 2023-01-17

**Authors:** Ruchira Colaco, Clement Appiah, Anne Staubitz

**Affiliations:** 1 Institute for Organic and Analytical Chemistry, University of Bremen, Leobener Str. 7, 28359 Bremen, Germany; 2 MAPEX Center for Materials and Process, University of Bremen, Bibliothekstr. 1, 28359 Bremen, Germany

**Keywords:** azobenzene, bimodal phase transition, PNIPAAm, hydrogels, photoisomerization

## Abstract

Poly(*N*-isopropylacrylamide) PNIPAAm hydrogels were modified with a new azobenzene-containing co-monomer. In this work, light responsiveness as an additional functionality, is conceptualized to induce two phase transitions in the same material, which can be controlled by light. For a hydrogel with merely 2.5 mol% of this co-monomer, the lower critical solution transition temperature (LCST) was lowered by 12 °C (to 20 °C) compared to PNIPAAm (LCST at 32 °C), as analyzed by differential scanning calorimetry (DSC). The untreated unimodal endotherm split into a bimodal peak upon irradiation with UV-light, giving a second onset due to the switched (*Z*) isomer-rich regions, LCST*_H2.5%__-**(*Z*)**_ = 26 °C. On irradiation with 450 nm, leading to the reverse (*Z*) to (*E*) isomerization, the endotherm was also reversible. Thus, a photo-switchable hydrogel whose LCST and structure are tunable with the hydrophobicity-hydrophilicity of the (*E*) and (*Z*) isomeric state of azobenzene was obtained. The influence of the increase in the mol% of azoacrylate on the LCST was evaluated via DSC, in combination with NMR studies, UV-vis spectroscopy and control experiments with linear polymers. The large light-driven modulation of the LCST adds bistability in thermoresponsive hydrogels, which may open diverse applications in the field of soft robotics actuators.

## 1. Introduction

Poly(*N*-isopropylacrylamide) (PNIPAAm) is a thermoresponsive polymer due to the temperature-induced, coil-to-globule phase transition occurring in water [1,2]. Because of the temperature-dependent water-polymer, polymer-polymer and water-water interactions, the PNIPAAm polymer chains expand below, or conversely contract above, a critical regime known as the lower critical solution transition temperature (LCST) [2,3]. Owing to the higher water-polymer interaction at T < LCST, in comparison to the other interactions, the polymer has higher solubility at lower temperatures, which is in contrast to the typical solubility/temperature relationship, in which, polymer solubility increases at higher temperatures [4]. Above that (T > LCST), the polymer demixes out of the solution at the critical concentration (*ɸ*) [5]. PNIPAAm polymers show an off-zero limiting critical concentration, *ɸ*_L_ ≠ 0 when the temperature is other than the LCST. The deswelling of the polymer chains thus occurs sharply around the LCST, with considerable shrinkage in the polymers’ chain size and assembly [6,7]. Polymers with an LCST behavior have tremendous technological application due to the reversible change in hydrophilicity with temperature [8]. The reason for this lies in the combined structural extremes of the amphiphilic side chain and hydrophobic polymer backbone [9]. Thus, the crosslinked 3D networks of PNIPAAm, reswollen in water, undergo reversible volume change in response to temperature. They are termed thermoresponsive hydrogels, with an onset close to the physiological temperature of humans, LCST_PNIPAAm_ = 32 °C, similar to their linear polymers [10,11]. Such volumetric changes in hydrogels have great potential, e.g., in actuation and microfluidics systems [12,13]. For example, a PNIPAAM hydrogel was microengineered with ZnO-based microchannels to induce a 7.5-fold larger weight reduction in comparison to its bulk PNIPAM, which enhanced its thermoresponsive actuating motions [14]. Similarly, the hydrogel materials were modified to respond to different stimuli such as chemicals [15], pH [16] and electric field [16] to control deformation and recovery in the material. This has led PNIPAAm hydrogels to be the best-researched hydrogels, opening applications in the field of drug release [17], scaffold and tissue engineering [18,19].

Scientific attention is focused on further tuning the LCST, thus influencing the temperature at which the permanent network undergoes a volume change, which is crucial for designing actuating systems for many bio-medical applications, as organisms are very sensitive to even small temperature changes [13,19,20]. The polymeric composition of PNIPAAm determines the onset of the LCST and can be tuned with hydrophilic and hydrophobic co-monomers [21,22,23]. This change in the LCST temperature regime in copolymeric systems with NIPAAm is, however, only thermoresponsive. In order to induce a multi-stimulus LCST, light-responsive moieties, for example molecular photo-switches, need to be co-polymerized with NIPAAm [10,24]. The photoactive molecules can be reversibly switched between two isomeric states of varied dipole moments [24]. This difference in the polarity of the isomer could concomitantly influence the hydrophilic-hydrophobic properties of the hybrid hydrogel, thus modulating the onset of LCST [25]. The LCST temperature can thus be hypothesized to switch on and off with respect to the isomeric state of the photo-switch, driven by light irradiation.

Among the plethora of photochromic moieties, isomerizable azobenzene offers efficient reversibility and interconversion between its two forms: (*E*) and (*Z*) even when incorporated in polymeric materials [26,27,28,29,30]. Irie and co-workers first reported the light-tunable phase transition of linear PNIPAAm and azobenzene-based copolymers [24]. In this work, upon UV irradiation, a 6 °C change in the cloud point temperature (CPT) [31] of the polymer with an azobenzene content of 2.7 mol% was reported. A larger shift in the CPT for photo-thermoresponsive polymers was only observed in organic/aqueous solvent mixtures, which is a limitation factor for applications in biological systems [32]. The photo-switched CPT in PNIPAAm increased to 10 °C by terminally functionalizing the polymer with 4-(2-hydroxyethoxy)-4′-(2-(2-chloropropionyloxy)-ethoxy)azobenzene units [33]. Such large CPT for 1.5 mol% was only achieved in polymers with a very low molecular weight of 1.7–12.0 kDa [34]. A combination of hydrophilic *N*,*N*-dimethylacrylamide (DMA) with 4.5 mol% of the hydrophobic 4-phenylazophenyl acrylate indicated a shift of approximately 8 °C in the CPT upon UV irradiation in the linear polymers [27]. All these examples reported the LCST of linear polymers and not cross-linked hydrogels. This is very relevant, as the former are much easier to analyse and to modify, but due to their mechanical instability can only be used in limited (and complementary) applications.

The study in polymers encouraged the implementation of photochromes in PNIPAAm-based thermoresponsive materials in a host-guest system, triggering a reversible gel-to-sol conversion [29]. Light-induced conformational changes in azobenzene-based photoresponsive crosslinkers influence the hydrogel material stiffness [3,11] upon irradiation with UV or vis light, opening up applications in scaffold design that are beyond the scope of light-functional polymers [27,28]. Therefore, crosslinked systems such as hydrogels are desired for the incorporation of the photochromes as co-monomers, to control the LCST behavior and consequently exert a physical response in the material structure [35]. It has been reported for spirobenzopyran-modified PNIPAAm hydrogel that light induced a volume change in acidic medium due to the spiropyran-merocyanine isomerization [36] and the influence of the medium (pH) on the fast reversal from the hydrophilic merocyanine to the hydrophobic spiropyran [37]. A soft-actuating walker was developed with an *N*-isopropylacrylamide-*co*-spiropyran-*co*-acrylic acid system [38]. Additionally, a sulfonate-based water-soluble spiropyran-functionalized di(ethylene glycol) methyl ether methacrylate (DEGMA) and oligo(ethylene glycol) methyl ether methacrylate (OEGMA) hydrogel demonstrated negative phototaxis on light illumination [39]. Irrespective of the success with spiropyran-functionalized hydrogels, the photo-bleaching effect limits the cyclic stability of the photochrome to about 10 cycles [40]. The stability of the photo-switched isomer is also crucial to further investigating the change in the LCST in hydrogel.

An accurate determination of LCST in both polymers and hydrogels can be performed via calorimetric techniques such as differential scanning calorimetry (DSC) [25,41,42]. The DSC scans can reveal and differentiate the nature of the endotherm from a single, unimodal in case of PNIPAAm homopolymer to bimodal, two-phase transitions observed in PNIPAAm-azopyridine copolymers [43]. The LCST of cross-linked hydrogels is best observed by DSC as clound points in UV-vis require soluble polymers.

Currently, only Liu and co-workers. have investigated the photocontrol of LCST in hydrogels (as opposed to a linear polymer) via DSC [35]. They employed a water-soluble photochrome, 4-[(4-(acryloyloxy)ethoxy) phenylazo]benzoic acid as a co-monomer in PNIPAAm hydrogels. The hydrogel material showed an LCST shift of only 3 °C upon irradiation with UV light. By eliminating the linker of the previous moiety to incorporate 4-((4-(acryloyloxy)phenyl)diazenyl)benzoic acid as a co-monomer, an even lower effect of 1 °C on the LCST upon irradiation with UV light was present [44]. The literature suggests that the light-induced shift of the CPT/LCST in linear polymeric or crosslinked hydrogelpolymeric systems, functionalized with water-soluble photochromic molecules still remains very small in water [27,35,44]. The probable reason for this small shift is linked to the similarity of the aqueous solubility of the water-soluble photochromic derivatives to that of NIPAAm monomer [35]. Additionally, to the best of our knowledge, no quantification of the photochemical enrichment of the (*Z*) isomer post UV irradiation in the polymer/hydrogels has been associated to the LCST shift, measured via DSC. It would be of great interest to obtain and control a distinctive shift in the LCST of PNIPAAm crosslinked hydrogels by isomerizing the photochromic molecule such as azobenzene, making it a functional material.

To overcome the previous limitations, we developed a strategy for achieving a PNIPAAm hydrogel system with (*E*)-4-(*p*-tolyldiazenyl)phenyl acrylate or azoacrylate (**Azo(1)**) moiety, which is a non-water-soluble, neutral co-monomer. Although this meant a synthetically more challenging route, a greater effect of photo-switching upon the LCST is expected [35,45]. The covalent incorporation of a hydrophobic (*E*)-azobenzene moiety should influence the aqueous solubility of the PNIPAAm polymer chains in the hydrogel matrix and alter the resultant hydrophobicity of the network. The hybrid hydrogels should undergo the LCST at a much lower temperature. The polarity difference between the two isomers (*E*) to (*Z*) could thus be utilized to reversibly alter the hydrophobicity–hydrophilicity of the network, thus leading to a material with multiple phase transitions driven by light. The temperature range that is tunable by light, which concomitantly controls the structure of the gel, can be termed as the bistable temperature, ΔLCST*.

In this study, the photoconversion of the (*E*) and (*Z*) isomers of the monomer was investigated via ^1^H NMR spectroscopy for the monomer in an organic solution (because it was insoluble in water) and for the polymers and hydrogels in the aqueous medium, which is the relevant medium for hydrogel applications. The influence of the **Azo(1)** content (1, 2.5 and 5) mol% on the swelling properties, leading to a significant lowering of water retention in the network, is shown. Furthermore, the effect of the incorporation of the azoacrylate co-monomer on the LCST of the hydrogel with an increase in the azo content (1, 2.5 and 5) mol% via DSC was studied. The DSC endotherms showed a significant lowering of the LCST of PNIPAAm by 12 °C upon incorporating the hydrophobic azoacrylate moiety at an extent of a mere 2.5 mol%. As the azobenzene is photo-switchable between two isomers, (*E*) and (*Z*), with different dipole moments, the hybrid gel could be further oriented into two different states post synthesis. In the photostationary state (PSS), the hydrogel could be switched reversibly between hydrophilic (*Z*) and hydrophobic (*E*) states. A stimulus-responsive hydrogel with two transition temperatures, LCST**_H2.5%,_**_(***E***)_ = 20 °C and the UV-switched LCST**_H2.5%,_**_(***Z***)_ = 26 °C, that can be turned on and off based on the isomerization state (*E*) or (*Z*) of the azobenzene, is reported. The UV-switched hybrid gel with LCST**_H2.5%,_**_(***Z***)_ = 26 °C remained swollen at a higher temperature, whereas the non-treated gels with LCST**_H2.5%,_**_(***E***)_ = 20 °C shrunk at a lower temperature. Additionally, the phase transition endotherm of the (*Z*) state also revealed, for the first time, a bimodal feature in the hybrid hydrogels via DSC, which was reversible in nature on irradiation with visible light or heat. The shift in the LCST of 6 °C controlled by light is the bistable temperature, which is crucial for future applications of light-actuating soft materials. The light-driven LCST of azobenzene-functionalized hydrogels, adding bistability to the hybrid material with modulation of the material structure, is a key advancement in soft robotics.

## 2. Results

### 2.1. Synthesis: Azo-co-NIPAAm Hydrogels

To functionalize NIPAAm with a photochromic moiety, an azobenzene with an acrylate functional group, azoacrylate (**Azo (1)**), was chosen as a comonomer [26]. With the synthesis of functional polymers whose intended use requires their production on a larger scale, high yields and a small number of steps are important. The photochromic comonomer **Azo(1)** was obtained in two steps with an overall yield of 73% (for details, see Supplementary Material, Scheme S1). As ^1^H NMR spectroscopy is an important analytical technique for quantifying the isomerization ratio (*E*/*Z*) of **Azo(1)**, it was crucial that the peaks were easily identifiable and integratable. Hence, the methyl group (-CH_3_) in the *para* position was key in distinguishing the NMR shifts in the aromatic region by rendering the system an AA’XX’ system.

**Scheme 1:**
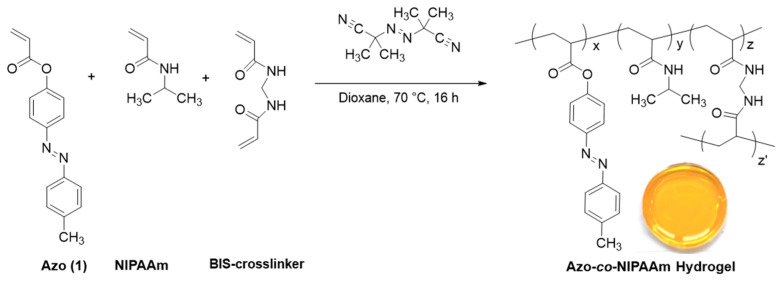
Synthetic scheme for Azo-co-NIPAAm hydrogel.

The introduction of a hydrophobic co-monomer into an amphiphilic NIPAAm and *N*, *N*’-methylenenbisacrylamide (BIS) mixture is not trivial (for details, see Supplementary Material, Tables S3–S6). The commonly used aqueous initiator system of ammonium persulfate (APS) with tetramethylethylenediamine (TEMED) as an accelerator resulted in the precipitation of **Azo(1)**, and did not lead to gel formation. As complete and fast mixing was paramount to the success of the synthesis, the reactions were performed in 1,4-dioxane, in which all components were well soluble. Consequently, the former initiator was replaced with azobisisobutyronitrile (AIBN), in order to thermally initiate polymerization at 70 °C. As AIBN has a half-life of 8 h at 70 °C, the reaction mixture was allowed to crosslink and cure for 16 h in order to obtain the azoacrylate-functionalized PNIPAAm, Azo-*co*-NIPAAm, hydrogels (Scheme 1).

Using the above reaction conditions, the amount of **Azo(1)** was varied (1, 2.5, 5 mol%), in order to obtain three respective Azo-*co*-NIPAAm hydrogel systems, namely **H_1%_**, **H_2.5%_** and **H_5%_**. The control PNIPAAm hydrogel, **H_0%_**, without the photochrome, was used as a reference for all further characterization. The content of the crosslinker *N*,*N*’-methylenenbisacrylamide (BIS) was also crucial in obtaining stable hydrogels across the range of the **Azo(1)** content (see Supplementary Material, Table S6). The crosslinker amount of 6.5 mol% resulted in stable hydrogels for all of the given formulations (Table 1).

### 2.2. UV-vis and ^1^H NMR Spectroscopy Study: Quantification of the Photoresponsiveness of Azoacrylate monomer Azo (1), Azo-co-NIPAAm Hydrogels and Polymers

**Figure 1:**
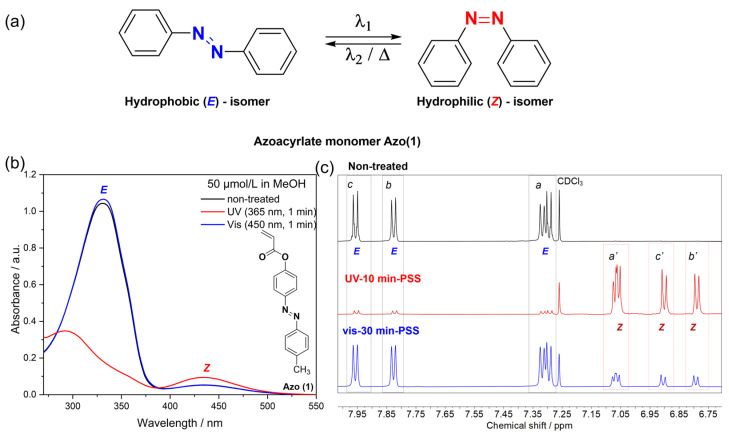
(**a**) Schematic diagram representing the light-induced reversible switching of azobenzene from the (*E*) to the (*Z*) isomer; (**b**) UV-vis spectra of the azoacrylate monomer, **Azo(1)**, in methanol (50 µmol/L), indicating reversible switching when irradiated with UV light (365 nm, 1 min) and vis light (450 nm, 1 min); (**c**) ^1^H NMR spectrum of the **Azo(1)** monomer in CDCl_3_ (4.88 mmol/L), indicating (*E*) isomer (>98%) in the non-treated form; mixture enriched with the (*Z*) isomer after irradiation with UV light (red) and back-switched to an (*E*)-isomer-rich mixture upon irradiation with vis light (blue).

- Monomer:

The photochemical properties of the azoacrylate monomer **Azo(1)** (50 µmol in methanol) were investigated via UV-vis spectroscopy, to assess its suitability as a light-switchable monomer. Initially, the photostationary state (PSS), which is the equilibrium isomer composition at a specific wavelength of irradiation, was investigated in the reversible photochemical reaction between the (*E*) and (*Z*) isomers. Varying the UV and vis light irradiation time (0 s, 30 s, 1 min and 2 min), the PSS was observed to have been reached after 1 min, as the spectra showed no further changes in the absorbance maximum (see Supplementary Material, Figure S1). On irradiation of the sample with UV light (λ = 365 nm, 1 min, 1030 mW, nominal intensity = 11.2 mW/cm^2^), the azobenzene moiety underwent a π-π* transition leading to an isomerization from the thermodynamically stable (*E*) isomer to the (*Z*) isomer (Figure 1a,b). Subsequently, the reversing reaction from the (*Z*) to (*E*) isomer due to the n-π* transition was induced photochemically with blue light (λ = 450 nm, 1 min, 900 mW, nominal intensity = 9.8 mW/cm^2^). The UV and vis light dosage received by the sample after different irradiation times is described in the Supplementary Material, Section S10, (Table S12). Finally, the thermal relaxation of the metastable (*Z*) isomer to the (*E*) isomer at 25 °C was monitored for 16 h by recording a spectrum at intervals of 30 min. The first-order kinetic profile indicated a half-life *t*_1/2_ = 1.7 days, which is the time required for the (*Z*) isomer’s concentration to decrease to 50% (see Supplementary Material Section S8, Figure S2). A slow thermal reversal of the **Azo(1)-*E*** is advantageous for its incorporation in hydrogel materials, as the effect of the (*Z*)-isomer-enriched state is longer lasting, which is important in application: light can be controlled, and the thermal relaxation is a background reaction. Furthermore, a system with a long half-life can be investigated in-depth using a greater number of techniques.

Quantitative elucidation of the isomer interconversion between (*E*) and (*Z*) forms was carried out using ^1^H NMR spectroscopy. The **Azo(1)** was dissolved in deuterated methanol and chloroform in the desired concentration range (~4.0 mmol/L) for ^1^H NMR analysis and the spectrum for the non-treated UV- and vis-light-irradiated sample was recorded. As the isomeric enrichment in CDCl_3_ was higher than in MeOD (see Supplementary Material, Figures S4 and S27), the integrals of the former are used in the discussion. The ^1^H NMR spectrum of the non-treated azoacrylate monomer **Azo(1)** post synthesis indicated that only the thermodynamically stable (*E*) isomer was present (Figure 1c).

The NMR shifts of the aromatic protons were observed downfield as a multiplet peak, *a* (7.29 ppm), and a set of two doublets, *b* (7.85 ppm) and *c* (7.96 ppm), respectively. Illumination with 365 nm for 10 min produced the photo stationary state (PSS), which was 93% enriched with the (*Z*) form. This could be seen by the upfield shift of the aromatic signals as a multiplet, *a’* (7.29 ppm), and two doublets, *b’* (6.77 ppm) and *c’* (6.89 ppm), respectively. A longer irradiation time of 30 min (UV dosage = 20.2 J/cm^2^) was required to achieve a PSS at 450 nm, resulting in a mixture of 80% being enriched with the (*E*) form. For the integration of signals in the PSS (365 nm and 450 nm) with the full spectra, see Supplementary Material, Section S13 (Figures S25 and S26).

Such an effective photochrome **Azo(1)** was ideal for incorporation as a co-monomer to produce the Azo-*co*-NIPAAm hydrogel networks. As these networks are reswollen in water, the photo-switchability of **Azo(1)** covalently linked to a linear polymer chain in an aqueous environment is crucial to investigate as a model system that can be more easily analyzed.

**Figure 2:**
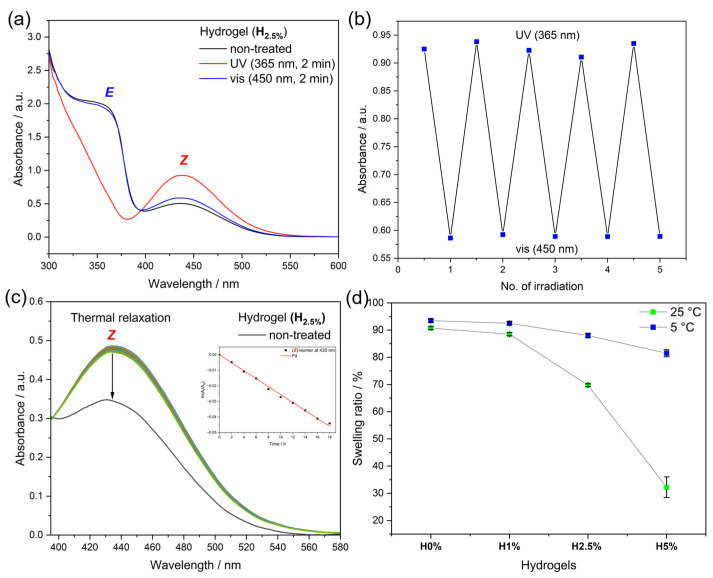
(**a**) UV-vis absorption spectra of dried Azo-*co*-NIPAAm hydrogel film (**H_2.5%_**, 0.1-mm-thickness) following synthesis and work-up: non-treated sample (black curve); (*Z*) isomer upon irradiation with UV light, λ = 365 nm for 2 min (red curve); (*E*) isomer converted by λ = 450 nm for 2 min (blue curve); (**b**) absorption intensity at λ_max_ = 439 nm tracking the five cyclic measurements, reversibly switching between (*E*) and (*Z*); (**c**) thermal relaxation of hydrogel film (**H_2.5%_**, 0.7 mm in thickness) swollen in D_2_O, after irradiation with UV light, λ = 365 nm for 1 min (PSS), and recorded at intervals of 30 min for the (*Z*) isomer. Inset: first-order kinetics of **H_2.5%_** film from the PSS (450 nm) at λ_max_ = 430 nm in deionized H_2_O; (**d**) swelling ratio of the control **H_0%_**, and the functional **H_1%,_ H_2.5%_**, and **H_5%_** hydrogels for four samples, S1 to S4, after equilibrating for 24 h at 25 °C and 5 °C, respectively.

- Azo-*co*-NIPAAm Hydrogels:

Monitoring the switchability of the azobenzene moiety in the hydrogel network via UV-vis spectroscopy was of key importance in further examining whether controlling the lower critical solution transition temperature (LCST) by light was viable. Firstly, the photochemical enrichment of the (*E*) and (*Z*) isomers was achieved in the dry **H_2.5%_** film on irradiation with 450 nm and 365 nm, respectively, for 2 min (Figure 2a). Secondly, the absorption intensity of the (*Z*), λ_max_ = 439 nm, was used to track the interconvertibility between the two isomers. Repeating the irradiation cycles fives time showed efficient reversibility between the (*E*) and (*Z*) isomers with no significant signs of photo fatigue (Figure 2b). The results prove the principle that the azobenzene motif covalently linked into a crosslinked polymer network of PNIPAAm can be switched reversibly.

How does the isomerization state (*E*) and (*Z*) of the azobenzene affect the LCST of the functional hydrogel? For the correlation of two very different techniques, UV-vis spectroscopy and differential scanning calorimetry (DSC), the changes induced in the hydrogel material during the measurements need to be taken into account. An LCST measurement involves the temperature ramp from 5 °C to 50 °C at a heating and cooling rate of 10 K/min, which typically takes 30 min. Thus, it was crucial to examine the stability of the enriched isomers (*E*) or (*Z*) with respect to time and external thermal energy. For the thermodynamically stable (*E*) isomer, the enrichment was expected not to be affected in the dark at an ambient temperature of 25 °C. Thus, investigating the effect of the predominant (*E*) isomer would be possible. However, the photoactivated metastable (*Z*) isomer undergoes thermal relaxation to (*E*) at 25 °C. Therefore, it was of key importance to understand how fast the thermal relaxation of (*Z*) to (*E*) takes place at 25 °C in the hydrated hydrogel. The swollen hydrogel film, **H_2.5%_**, reached PSS at 25 °C under UV light (365 nm, 1 min). The spontaneous back-conversion at 25 °C was tracked for 18 h, at intervals of 30 min. The first-order kinetics of **H_2.5%_** indicated a long half-life, *t*_1/2_ = 11.2 days (Figure 2c). This is considerably longer than the half-life of the monomer in an organic solvent. A slow thermal relaxation of the meta-stable isomer in the hydrogel material ensured investigation of the effect of the predominant (*Z*) isomer on the LCST measurement.

The dry film (0.1 mm thickness) represents a shrunken state of the **H_2.5%_** hydrogel, which on reswelling in water expanded to a thickness of 0.7 mm. In the dry film, the PSS was reached after 2 min, whereas for the swollen film, this took only 1 min. Additionally, the absorbance value was ca. double for the UV-vis spectra in the dried film in comparison to the reswollen film of **H_2.5%_**. The solid-state UV-vis measurements were sensitive to the thickness of the films and their absorbance values. Therefore, the swelling capacity of the varied hydrogel film plays a significant role in performing these measurements. Hydrogels with 1 mol% of azo content had a swelling ratio of 88% and 92 % at 25 °C and 5 °C respectively, that was in the same range as the control hydrogel **H_0%_** (Figure 2d). Due to which, the **H_1%_** film swelled >1.0 mm, giving a plateau-like absorbance feature, whereby no significant absorbance maxima could be observed. The incorporation of 2.5 mol% reduced the swelling capacity of **H_2.5%_** to 70% at 25 °C, whereas on cooling to 5 °C, equilibrium at 88% was attained. A steep reduction in the swelling capacity was observed for **H_5%_**, with swelling occurring to only 32% at 25 °C and 81% at 5 °C. The **H**_5%_ film thus retained a shrunken state, giving high absorbances >5.0 a.u, limiting the measurement of the respective hydrogels systems. Thus, the correlational investigation between UV-vis and ^1^H NMR was only carried out for the swollen **H_2.5%_** hydrogel.

The quantification of the (*E*) and (*Z*) isomers in the hydrogels via ^1^H NMR spectroscopy was challenging, as crosslinked systems give rise to very broad signals (see Supplementary Material Section S13, Figure S28). The ^1^H NMR spectra of the **H_2.5_**_%_ hydrogel in D_2_O indicated the overlap of the -NH signal of the NIPAAm monomer with the aromatic signals of the **Azo(1)** comonomer in the range of 8.6 ppm to 7.2 ppm. By heating the NMR sample to 50 °C for 10 min, a proton–deuterium exchange was facilitated, ameliorating this issue, but the NMR signals remained non-integratable, making it impossible to quantify the isomeric ratio of (*E*) and (*Z*) of the azobenzene in the hydrogel (see Supplementary Material, Figure S29). Therefore, the switchability of the azobenzene and the *E*/*Z* ratio at PSS (365 nm and 450 nm) of the linear polymer in aqueous medium, which should present similar photochemical behavior, was used as a model system. Thus, the corresponding polymers were synthesized to quantify the (*E*) and (*Z*) isomer percentages via ^1^H NMR spectroscopy.

**Figure 3:**
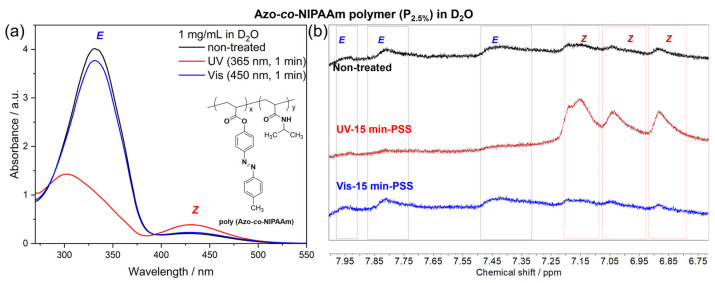
(**a**) UV-vis spectra of the Azo-*co*-NIPAAm polymer, **P_2.5%_**, effectively switching in D_2_O (1 mg/mL) and retaining the photochromic properties in aqueous medium; (**b**) ^1^H NMR spectra of the polymer **P_2.5%_** in D_2_O (10 mg/mL), indicating a mixture of 68% (*E*) and 32% (*Z*) in the non-treated state, an enriched (*Z*) isomer after irradiation with UV light (red), and a mixed form when back-switched with vis light (blue).

- Azo-*co*-NIPAAm Polymers:

Free radical polymerization using the same reaction conditions as optimized for the hydrogel synthesis gave the corresponding Azo-*co*-NIPAAm polymers, **P_1%_**, **P_2.5%_**, **P_5%_**, with contents of 1, 2.5 and 5 mol% of **Azo(1)**, respectively. The pristine polymer of PNIPAAm (**P_0%_**) and the three functionalized copolymers yielded molecular weights ranging from 20 to 32 kDa (see Supplementary Material, Table S14 and Figure S19 for the GPC plots). To prove the covalent incorporation of the **Azo(1)** in the polymeric chain, the GPC measurement was recorded with the UV cut-off wavelength set at 340 nm. All three photosensitive polymers shared an elution profile in which the absorbance signal increased (**P_1%_** to **P_5%_**), confirming the attachment of the photochrome (see Supplementary Material, Figure S20). On the other hand, the control PNIPAAm polymer did not show a peak in the UV-vis detected elution profile at 340 nm. For the UV-vis studies, polymer solutions were prepared in D_2_O for **P_1%_**, **P_2.5%_** (1 mg/mL), and **P_5%_** (0.5 mg/mL); the latter sample was prepared with a lower concentration due to the high absorbance found for the previous concentration. Reversible (*E*) to (*Z*) isomerization was observed for solution **P_2.5%_**, as shown in Figure 3a, and for **P_1%_**, **P_5%_** (see Supplementary Material, Figure S3a,b). The **Azo(1)**-functionalized PNIPAAm polymers were effectively switched in aqueous medium, similar to the pure monomer. All of the polymers showed excellent interconvertibility over 10 irradiation cycles (see Supplementary Material, Figure S3c). The slope of the first-order thermal relaxation kinetics of **Azo(1)** gave a rate constant k = −6.81 × 10^−3^ h^−1^, and the calculated half-life was *t*_1/2_ = 4.2 d in methanol. By comparison, the rate of thermal relaxation kinetic decreased with increasing **Azo(1)** content in the co-polymers in D_2_O. In the case of **P_1%_**, the rate was k = −3.4 × 10^−3^ h^−1^ and the calculated half-life was *t*_1/2_ = 8.5 d; for **P_2.5%_**, k = −3.04 × 10^−3^ h^−1^ with *t*_1/2_ = 9.5 d; and for **P_5%_**, k = −2.46 × 10^−3^ h^−1^ with *t*_1/2_ = 11.7 d. The monomer relaxed more quickly than the polymers, which is probably due to the different solvents.

To quantify the amount of each isomer before irradiation and in the photo stationary state (PSS), the ^1^H NMR spectra of **P_1%,_ P_2.5%,_ P_5%_** (D_2_O, 10 mg/mL) were recorded at 25 °C. Although the incorporation of azobenzene imparts hydrophobicity (*E*) to the amphiphilic nature of the PNIPAAm polymer, the aqueous environment led to some enrichment of the azobenzene in the (*Z*) configuration, as the Z-isomer is more polar. This is evident from the ^1^H NMR spectrum of **P_2.5%_** as synthesized in the non-treated state, where six broad signals can be seen in the aromatic region (Figure 3b). These represent the mixed form of the two isomers, 68% (*E*) and 32% (*Z*) (see Supplementary Material, Table S11, Figure S31). This is in contrast to the thermodynamically stable (*E*) isomer observed in the ^1^H NMR spectrum of the as-synthesized non-treated monomer **Azo(1)** in CDCl_3_. The difference in the ^1^H NMR shift of ~1.0 ppm enabled us to resolve the two isomers (*E*) and (*Z*) clearly, when they were in the side chain of the polymer. In this part of the study, it can be concluded that the solvent affects both the solubility and the photo isomeric equilibrium of the Azo-*co*-NIPAAm polymers.

Furthermore, to investigate the time required and the isomer percentage at the PSS, the polymer solutions were irradiated with UV and vis light, in sequence. On irradiation with UV light (365 nm, 15 min), the polymer attained a PSS with a (*Z*)-isomer-enriched state of 96%. On back-irradiation with blue light (450 nm, 15 min), the azobenzene achieved a PSS in which the (*E*) isomer was enriched to 75%. The NMR shifts for the (*E*) and (*Z*) isomer in the polymer sample qualitatively matched the shift observed for the **Azo(1)** in CDCl_3_. Further increasing the irradiation time to 30 min did not increase the (*E*) isomer amount in **P_2.5%_**_._ This confirms that 15 min of irradiation was sufficient to enrich the (*E*) isomer (75%), which was greater than the 68% observed in the non-treated polymer, **P_2.5%_**.

The ^1^H NMR spectrum for the non-treated **P_1%_** polymer was 80% (*E*) and 20% (*Z*). On irradiation with UV (365 nm, 15 min), 86% of the (*Z*) isomer was enriched, whereas upon back-switching with blue light (450 nm, 15 min), 75% (*E*) was attained (see Supplementary Material, Figure S34, Table S11). On the other hand, the polymer with the highest mol% of **Azo(1)** co-monomer, **P_5%_** (5 mol%); remained insoluble in D_2_O due to its high hydrophobicity. When exposed to UV irradiation, the polymer coagulated, and thus the ^1^H NMR shift did not yield integratable signals (see Supplementary Material, Figure S35). Among the three polymeric systems, the sample with 1 and 2.5 mol% of **Azo(1)** (**P_1%_**, **P_2.5%_**) showed the most effective photoconversion to (*Z*) isomer and back. Hence, their corresponding hydrogels, **H_1%_** and **H_2.5%_**, were investigated in detail in thermal analyses via differential scanning calorimetry (DSC).

### 2.3. Photo Tunable LCST of Azo-co-PNIPAAm Hydrogels via DSC

The effect of the isomerization of azobenzene on the LCST of the PNIPAAm hydrogels was investigated via differential scanning calorimetry (DSC). The equilibrated swollen control PNIPAAm hydrogel (**H_0%_**) and the Azo-*co*-NIPAAm hydrogel, (**H_2.5%_**) hydrogels in deionized water were subjected to a temperature ramp from of 5 °C to 50 °C. An ideal heating rate in thermal analyses gives narrow, baseline-separated thermal features. Three different rates of heating and cooling (10, 15 and 20 K/min) were used to record the phase transition (see Supplementary Material, Figure S6). The narrowest signal with the best signal-to-noise ratio was obtained when running the DSC with 10 K/min. In addition to a narrow endotherm (heating), the exothermic feature in the cooling curve was only detected with the 10 K/min cooling rate for (**H_0%,_ H_1%_**, **H_2.5%_**). However, for **P_5%_** and **H_5%_**, with a heating rate of 10 K/min it was not possible to distinguish a clear onset of the LCST (see Supplementary Material, Figures S9 and S10). Therefore, the heating rate was lowered to 1 K/min to obtain a baseline separated signal. An isothermal segment of 10 min each at the start and end temperature ensured thermal equilibrium in the sample before the respective dynamic ramp. The irradiation of the hydrogels was carried out at 5 °C to minimize (a) heating effects from the UV source and (b) irradiating around the investigated LCST range. The integrals for determining the onset were fixed in the range of 15 °C to 40 °C for Azo-*co*-NIPAAm hydrogel, **H_2.5%_** and the onset of the phase transition was detected by the DSC software STARe. The phase transitions with respect to light treatment were denoted as follows: nontreated sample = LCST_non-treated_, UV (λ = 365 nm) switched = LCST*_(***Z***)_, vis (λ = 450 nm) switched = LCST*_(***E***)._ The temperature difference between the two switched states that can be controlled with light was termed the bistable temperature, ΔLCST*.

**Figure 4:**
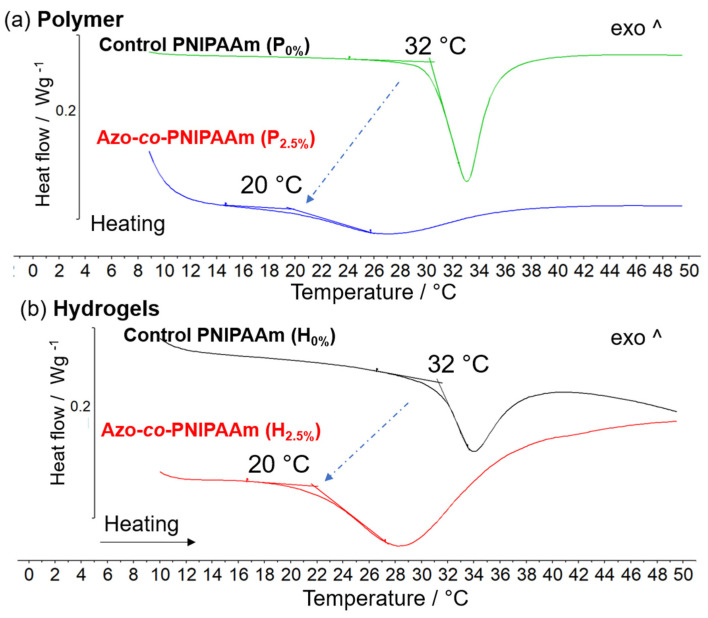
DSC scans (10 K/min) comparing the effect of incorporating 2.5 mol% of **Azo(1)** on the lower critical solution temperature (LCST) of the control PNIPAAm in (**a**) polymers **P_0%_** and **P_2.5%_** and (**b**) hydrogels **H_0%_** and **H_2.5%_**.

The onset of the LCST in the control PNIPAAm polymer, **P_0%_**, and hydrogel, **H_0%_**, was LCST**_P0/H0%_** = 32 °C (Figure 4a). The two systems were used as a reference to examine the effect of the increase in **Azo(1)** content on its LCST. 

In both cases Azo-*co*-PNIPAAm polymer/hydrogel an increase in **Azo(1)** from 1 mol% to 2.5 mol% lowered the phase transition temperature by 6 °C, LCST**_P1/H1%_** = 26 °C, and by 12 °C, LCST**_P2.5/H2.5%_** = 20 °C, respectively (Figure 4b, and see Supplementary Material, Figures S8 and S11). Further increase to 5 mol% resulted in a steep lowering of the phase transition temperature by 23 °C, LCST**_P5/H5%_** = 9 °C.

A narrow peak for the control PNIPAAm polymer, **P_0%_**, and hydrogel, **H_0%_**, was observed, due to the homopolymeric nature of the identical PNIPAAm chains. However, a broader endotherm for all of the three Azo-*co*-PNIPAAm polymers (**P_1%,_ P_2.5%,_ P_5%_**) and hydrogels (**H_1%_**, **H_2.5%_** and **H_5%_**) was observed. The broadening of the phase transition demonstrates the heterogeneity of the co-polymeric system. The effect of the incorporation of the **Azo(1)** in decreasing the LCST across all amounts (1, 2.5 and 5 mol%) was identical in both the linear and crosslinked materials. It is highly likely that the polymerization of the linear and crosslinked network incorporates same amount of the photochrome as a co-monomer. Thus, the photo-switchable isomeric enrichment of (*E*) and (*Z*) isomers obtained from the functional polymers via ^1^H NMR can be approximated to the hydrogel system as well.

**Figure 5:**
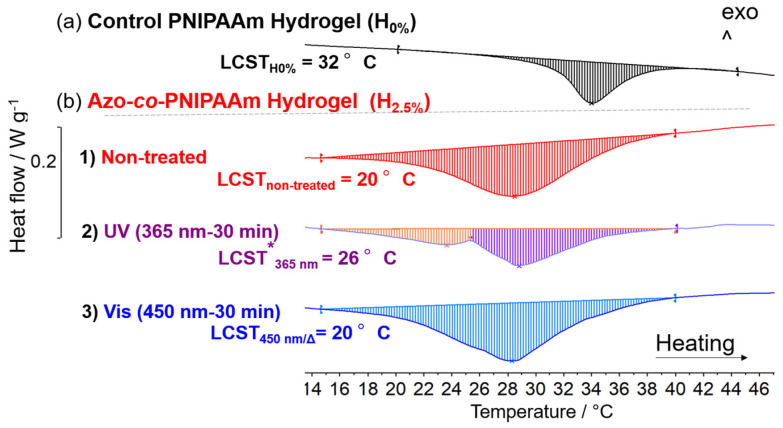
DSC scans (10 K/min) comparing the lower critical solution temperature (LCST) of the Control PNIPAAm (**H_0%_**) to the azobenzene (2.5 mol%)-functionalized Azobenzene-*co*-PNIPAAm hydrogels (**H_2.5%_**) hydrogels. (**a**) The onset of the LCST of **H_0%_** in agreement with the literature range between 32 °C for pure PNIPAAm-based systems. (**b1**) A 12 °C lowering of the LCST in the **H_2.5%_** hydrogel relative to **H_0%_**, indicating the effect of incorporating hydrophobic azobenzene in PNIPAAm; (**b2**) A bimodal LCST in the photochromic **H_2.5%_** hydrogel with a second endothermic transition temperature, LCST*_H2.5%-(*Z*)_ = 26°C on irradiation UV light (λ= 365 nm, 30 min); (**b3**) reversal of the LCST by irradiation with blue light (λ= 450 nm, 30 min), LCST**_H2.5%-(*E*)_** = 20 °C.

The non-treated hydrogel represented the thermodynamically stable isomer (*E*) in 68% and (*Z*) 32% with LCST**_H2.5%non-treated_** = 20 °C (Figure 5b). The first heating curve of the measurement, however, showed a slight bimodal character (see Supplementary Material, Figure S13) above 25 °C, which was smoothed out in the second heating curve (Figure 5b). In order to study the influence of the light as a stimulus for controlling the LCST, the hydrogel **H_2.5%_** was irradiated for increasing durations with UV and vis light (15 min, 30 min and 240 min) (see Supplementary Material, Figures S14 and S15), and thus increasing UV dosages (see Supplementary Material for the UV dosage values). No change in the LCST endotherm was observed on UV irradiation (λ = 365 nm, 15 min). The duration of irradiation increased the amount of UV light (λ = 365 nm, 30 min, intensity = 11.2 mW/cm^2^), thus isomerizing the photochrome to the (*Z*) form in a higher amount, i.e., 96%. As a result, the endotherm split from a unimodal into a bimodal curve, indicating two phase transitions in the first heating curve. The onset of the second endotherm represents a new phase transition temperature, LCST*_H2.5%__-**(*Z*)**_ = 26 °C. A further increase in the UV irradiation did not significantly alter the nature of the bimodal curve (see Supplementary Material, Figure S14). Thus, UV irradiation for 30 min enriched the (*Z*) isomer to its PSS state, i.e., from 22% to 96%. The hydrogel was back-switched with blue light (λ = 450 nm, 30 min, 9.8 mW/cm^2^), resulting in 75% (*E*). A reversal in the transition, LCST*_H2.5%-(*E*)_ = 20 °C, largely representing a unimodal endotherm similar to the non-treated hydrogel, was detected. Only the minor bimodal character of the 25% unswitched (*Z*) isomer could be observed. Additionally, the UV-switched LCST, as a bimodal feature, was only observed in the first heating cycle. Complete thermal relaxation of the (*Z*) to the (*E*) isomer took place at the end of the first heating cycle due to the elevated temperature of 50 °C. All of the consecutive heating cycles therefore resulted in it regaining its unimodal nature (see Supplementary Material, Figure S16).

On the other hand, for the hydrogel with the lowest **Azo(1)** content, **H_1%_**, the onset was recorded at LCST**_H1%-nontreated_** = 26 °C. On irradiation with UV light (λ = 365 nm, 30 min), LCST*_H1%-(*Z*)_ = 28 °C, representing a 2 °C shift to a higher temperature. Additionally, the back irradiation with vis light (λ = 450 nm) effectively reversed the LCST to 26 °C following a longer exposure time of 60 min (see Supplementary Material, Figure S17). On the other hand, no LCST shift in the hydrogel with the highest content of **Azo(1)**, **H_5%_**, was observed in response to UV or vis light (see Supplementary Material, Figure S18). The onset of the LCST remained constant at LCST**_H5%-nontreated/(*E*)/(*Z*)_** = 9 °C. The thermodynamic characteristics of the hydrogels are given in Table 2, which were determined on the basis of at least four samples for the non-treated and UV-switched hydrogels, along with the standard deviation (see Table S13).

### 2.4. Light Driven Structural Modulation in Azo-co-NIPAAm Hydrogels (H_2.5%_)

**Figure 6:**
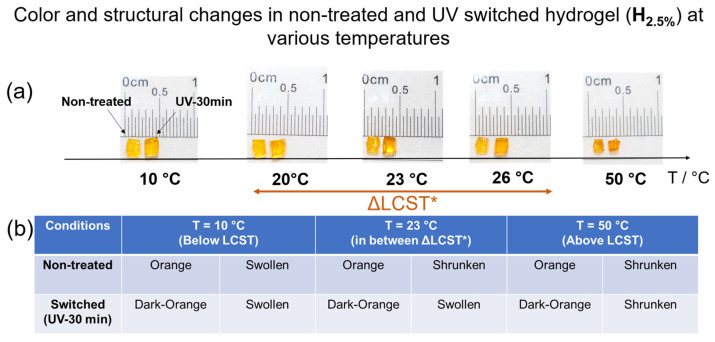
Photographic images of hydrogel (**H_2.5%_**) in non-treated and UV-switched state at various temperatures: (**a**) 10 °C (below LCST), the bistable temperature range 20 °C to 26 °C with a central temperature 23 °C and 50 °C (above LCST); (**b**) tabulated information on the color and structural change in the hydrogel at the forementioned temperatures in °C.

The hydrogel with the largest bistability of 6 °C, **H_2.5%_**, was used to showcase the effect of UV-switching on the structure of the hydrogel in comparison to the non-treated sample (Figure 6). On UV irradiation of one sample for 30 min, the color of the hydrogel turned dark orange, in contrast to the non-treated hydrogel. At 10 °C, which is below LCST, both samples remained in the swollen state. The temperature was raised to the start of the bistable temperature, 20 °C, such that only the volume change in the non-treated hydrogel would commence. This was clearly evident at 23 °C, in between the ΔLCST*, at which point non-treated sample shrank whereas the UV-switched sample remained swollen. The UV-switched sample retained its swollen state up to 26 °C, the end of the ΔLCST*, whereas the non-treated hydrogel continued to shrink. Further increasing the temperature to 50 °C, above the LCST, resulted in shrinkage in both of the gels. The photo-induced LCST shifted to higher temperatures, thus retaining the hydrophilicity of the UV-treated hydrogels.

## 3. Discussion

The azoacrylate moeity **Azo(1)** serves as an ideal photochromic candidate for co-polymerization with NIPAAm to produce functional hydrogels, due to its excellent photochemical properties, which include rapid rate of photo-isomerization and excellent interconvertibility between the (*E*) and (*Z*) isomer. The PSS of all three functional co-polymers **P_1%_**, **P_2.5%_**, **P_5%_**was attained within 1 min of UV irradiation, indicating a fast photoisomerization, even in an aqueous medium. Additionally, the cyclic switching of the co-polymers with consecutive UV and vis irradiation over 10 repetitions demonstrated that the **Azo(1)** was stable against photo-fatigue when covalently linked into PNIPAAm. A similar rapid photochemical isomerization and cyclability was achieved for **H_2.5%_**, resulting in the production of a light-responsive hydrogel. The thermal relaxation kinetic rates varied as the systems grew from a monomer to polymer to a crosslinked network. In comparison to the reported azobenzene acrylate moiety without the -*CH_3_* group, used as a co-monomer to functionalize DMA polymers, the half-life time of **Azo(1)** in the PNIPAAm co-polymer **P_2.5%_** was approximately 17 times longer [27]. Our study revealed that the half-life of azobenzene copolymerized with PNIPAAm had higher stability than the DMA-based co-polymers [27]. When compared to its crosslinked hydrogel systems, the rate of the first-order kinetics in **H_2.5%_** was k = −2.56 × 10^−3^ h^−1^ with a *t*_1/2_ = 11.2 d, which is 2.6 times slower than its corresponding polymer, **P_2.5%_**. The lower relaxation rate can be attributed to the low degrees of freedom of the polymer chains between two crosslinked points, i.e., in a gel, in comparison to a free linear polymer chain in solution. The results revealed that a light-switchable hydrogel with fast photoisomerization and slow thermal relaxation of the (*Z*) isomer at 25 °C temperature was produced. As a temperature gradient was paramount for determining the phase transition via DSC (5 °C to 50 °C), slow thermal back-switching was necessary, as it is a background reaction, and light should be the main stimulus for achieving controllable bistability. Because of the long half-life times, the effect of the UV irradiation on increasing the LCST to a higher temperature can be clearly distinguished in the first heating curve of the DSC measurement of **H_2.5%_**. The consecutive heating curves, on the other hand, reveal no history of light irradiation due to thermal relaxation. The endotherm reverses to a unimodal, continuous character, indicating the back conversion of (*Z*) to (*E*). The isothermal segment was able to rapidly convert (*Z*) to (*E*) in 10 min at 50 °C, where attaining the half-life at 25 °C had required 11.2 d. From this it can be concluded that only at elevated temperature, i.e., T >> LCST, are the time scale of thermal relaxation and photoisomerization similar.

The **H_1%_** with the lowest amount of the **Azo(1)** content showed a phase transition 6 °C lower compared to the homopolymer hydrogel, **H_0%_**. The lower temperature shift in the LCST in the functionalized hydrogels was due to the hydrophobic nature of the (*E*) isomer. This shift was thus termed a “hydrophobic shift” due to the non-polar (*E*) isomer. The (*E*) to (*Z*) photoisomerization of azobenzene led to a dipole change from 0.0 D (1 Debye, D = 3.33564 × 10^−30^ C m) to 3.1 D [24,25,46]. Therefore, when the hydrogel was irradiated with UV (30 min), the enrichment of the (*Z*) isomer, which is hydrophilic in nature, shifted the phase transition 2 °C towards the PNIPAAm LCST. The shift arose when the hydrogel attained a configuration with 86% enriched (*Z*). The back-conversion of (*Z*) to (*E*) required a longer irradiation time with vis light (60 min) to reverse the LCST to 26 °C. The effect of the isomerization on the polarity of PNIPAAm chains in the hydrogel can thus be seen at low levels of **Azo(1)** content with both the UV and vis light irradiation. The temperature difference of the hydrogel when predominantly in (*E*) or (*Z*) form, which can be tuned using light as the stimulus, was termed the bistable temperature, ΔLCST*. Photocontrol of the LCST, indicating that ΔLCST*_H1%_ = 2 °C, was possible at only 1 mol% of **Azo(1)**. When compared to the first reported photocontrol in linear copolymeric systems of NIPAAm and *N*-(4-phenylazophenyl)acrylamide, no photo-tuning was reported for 1 mol% of the photochromic moiety when investigated via turbidity measurement [24]. We thus report that the photocontrol of the LCST in order to increase or decrease the temperature on the basis of the isomeric state of the covalently linked **Azo(1)** in turn changes the hydrophilicity-hydrophobicity of the hydrogel network.

Polarity changes in the network due to the hydrophilic character of the light-induced (*Z*) form was the highest with 2.5 mol%, **H_2.5%_**, as the phase separation from the predominantly (*E*) character altered the endothermic characteristic from being of a unimodal continuous, to a bimodal discontinuous quality. The bistable temperature was ΔLCST*_H2.5%_ = 6 °C, which, to the best of our knowledge, is the highest reported in crosslinked hydrogels via DSC analyses. When the hydrogel **H_2.5%_** was switched using UV light, the hydrophobic (*E*) isomer was converted to the hydrophilic (*Z*) isomer to an extent of 96%, showing a distinct onset at higher temperature. The switched (*Z*)-rich form can be reversed back to the (*E*) form upon irradiation or with thermal energy. An indication of the reversible switching in the hydrogels was the recurrence of the predominant single-phase transition temperature upon irradiation with vis light at 450 nm for 30 min.

Our observations are in agreement with previous reports in the literature stating that the highest degree of photocontrol of the LCST was with 2.5–2.7 mol% of azobenzene photochromic co-monomer, albeit in a linear polymer [24]. Further increase in the amount of the photochromic moiety (>3.7 mol%) yielded a CPT of 12 °C, which was not affected by UV-light treatment [24].Similarly, we observed that the hydrogel with 5 mol%, LCST**_H5%_** = 9 °C, was non-photoresponsive, as no change in the onset of the LCST was observed via DSC. This limits the utility of higher amounts of photochromes used as a co-monomer for obtaining light-induced modification in the phase transition of PNIPAAm-based systems. The corresponding crosslinked hydrogels, **H_1%_**, **H_2.5%_**, **H_5%_**, exerted a significant effect on the swelling ratio, with reductions of 88%, 70% and 30% with increasing azo content (1, 2.5 and 5 mol%). Such low degrees of swelling at 25 °C disqualify **H_5%_** as a hydrogel material. Our study reveals that an optimum amount of photochromic co-monomer of 2.5 mol% was necessary to achieve the highest level of photocontrol over LCST, as determined via DSC. The light-triggered LCST shift in the hydrogel was also responsible for controlling the volume change in the bulk sample. Such materials may find application in the development of photochromic micro actuator systems in the future.

## 4. Conclusions

Photo-/thermo-sensitive PNIPAAm hydrogels were produced by copolymerization of the photoactive azobenzene acrylate in order to control the lower critical solution transition temperature, LCST, using light as the stimulus. The photochromes underwent reversible photochemical reactions in the hydrogel, **H_2.5%_**, and the UV-activated (*Z*) isomer showed high stability, with a half-life of 11.2 d at 25 °C. The influence of the photo-induced hydrophilic (*Z*) isomer on the LCST of the functional hydrogel was therefore investigated on the basis of dynamic thermal measurements by means of differential scanning calorimetry (DSC). A shift in the onset temperature of the transition due to the hydrophilicity of the (*Z*) form relative to the hydrophobic (*E*) form yielded a bistable temperature range, ΔLCST* = 6 °C, which was highest for 2.5 mol% of **Azo(1)**. The ΔLCST* is twice as large as any other crosslinked system in water, functionalized with a neutral photochromic moiety. It can be concluded from our study that the photoactivated hydrogel was desirable when the time scales for photoisomerization and the thermal half-life around the bistable temperature were different. In addition, a large bistable temperature range was observed only with 2.5 mol% of **Azo(1)**, due to the high degree of enrichment of the (*Z*) isomer, which reached 96%, in aqueous medium. Concomitantly, the photo-switched hydrogel sample retained its swollen structure in the ΔLCST* temperature range, whereas the non-treated hydrogel structure underwent deswelling. Such light-induced control of the LCST in the hybrid azobenzene-functionalized PNIPAAm hydrogels could be used for volume change control in actuator systems.

## 5. Experimental Section/Methods

### 5.1. Materials

*N*-isopropylacrylamide (NIPAAm, 98%, Sigma-Aldrich, St. Louis, MO, USA), *N,N*’-methylenebisacrylamide (BIS 99%, Sigma-Aldrich), ammonium persulfate (APS, 97%, Sigma-Aldrich), *N,N,N*’,*N*’-tetramethylethylenediamine (TEMED) and Azobisisobutyronitrile (AIBN, 99%, Sigma Aldrich) were purchased. The NIPAAm monomer was purified via recrystallization at 50 °C from methanol (10.0 g in 10 mL of methanol). The recrystallisation of AIBN was carried out by warming methanol (10 mL) in a conical flask to 30 °C and dissolving approximately 7.00 g of AIBN with stirring. As AIBN is a thermal initiator, the recrystallization temperature was kept below 40 °C to prevent radical formation.

### 5.2. Methods

- Sample preparation and method for DSC measurement

The reswollen hydrogel was cut into rectangular strips with dimensions of 3.5 mm (length) × 2 mm (width) × 1 mm (thickness) and placed inside a 100 µL aluminum pan filled with 50 µL of distilled water. The open pan was placed inside the DSC and the furnace was closed with a quartz lid. The samples were irradiated at a wavelength of 365 nm or 450 nm (1030 mW and 900 mW) for a specified amount of time (15 min, 30 min, 1 h, 2 h, 3 h and 4 h). To eliminate the effect of heating from the light source, all of the irradiations of the sample were carried out at 5 °C. The distance of the lamp from the sample was 2.7 cm. The spot size of the lamps with wavelengths of 365 nm and 450 nm had a radius r = 16 mm. After irradiation, the sample was weighed and placed in a new 40 µL pan, which was hermetically sealed to avoid any loss of water during measurement.

For the determination of the LCST, a DSC method was used at temperatures from 5 °C to 50 °C, with an isothermal segment (10 min) at the starting and ending temperatures, and a dynamic temperature segment at 10 K/min. The method was run three times. The measurements were performed for at least four samples, and standard deviation was calculated (see Supplementary Material, Table S13). In Table 2, the values noted are the rounded-up temperatures obtained the LCST values of the four samples.

- Photographs for volume change in UV-switched hydrogel

Two samples were cut from the same hydrogel to compare the influence of UV-light irradiation on the non-treated gel. Both hydrogels were first equilibrated at 5 °C for 24 h. One sample was irradiated with UV light at 5°C for 30 min and again equilibrated for 1 h at 5 °C. Using a Linkam heating stage, the two samples were heated to temperatures of 10, 20, 23, 26 and 50 °C, respectively, while equilibrating the sample at each temperature for 10 min. Photographic images were recorded using a Canon EOS 700 D Camera with a white background and a calibration scale.

## Figures and Tables

**Table 1:** Formulations for the control PNIPAAm and Azo-co-NIPAAm hydrogels.

Sample	Azo /Mol%	Azo /mg	NIPAAM /mg	AIBN /mg	BIS /mg	Comments
**H_0%_**	-	-	102.5	10.0	9.0	Control gel
**H_1%_**	1.0%	2.5	101.5	10.0	9.0	Gel
**H_2.5%_**	2.5%	6.0	100	10.0	9.0	Gel
**H_5%_**	5.0%	12.0	97.5	10.0	9.0	Gel

**Table 2:** Thermodynamic characteristics of Control-PNIPAAm and Azo-PNIPAAm hydrogels.

Hydrogel	H_0%_	H_1%_	H_2.5%_	H_5%_
LCSTnon-treated	32°C	26 °C	20 °C	9 °C
LCST*(Z)	N/A	28 °C	26 °C	-
LCST*(*E*)	N/A	26 °C	20 °C	-
ΔLCST*	N/A	2 °C	6 °C	No LCST shift

## Data Availability

Not applicable.

## Supplementary Materials

The following supporting information can be downloaded at: https://www.mdpi.com/article/10.3390/gels9020075/s1, Figure S1. UV-vis absorption spectra of the azoacrylate monomer, **Azo(1)** in methanol (50 µmol) with varying irradiation time (0 s, 30 s, 1 min, 2 min) to reach the photostationary state (PSS): (a) UV irradiation (365 nm, nominal intensity = 11.2 mW/cm^2^) and (b) vis (450 nm, nominal intensity = 9.8 mW/cm^2^). The inset figure has the same X and Y-axis as the main plot. Figure S2. UV-vis absorption spectra of the azoacrylate monomer, **Azo(1)** in methanol (50 µmol): (a) Band of spectra after irradiating the solution to PSS (*Z*)-isomer with UV light, λ = 365 nm, for 1 min and recorded at an interval of 30 min (b) First-order thermal relaxation kinetics of **Azo(1)** from PSS (450 nm) at λ_max_ = 434 nm. Figure S3. UV-vis absorption spectra of Azo-*co*-NIPAAm polymers in D_2_O indicating reversible switching when irradiated with UV light (365 nm, 1 min); and vis light (450 nm, 1 min) for (a) **P_1%_**; (b) **P_5%_**; (c) The first-order thermal relaxation kinetics at PSS (450 nm) for **P_1%_**; **P_2.5%_**; **P_5%_** and reversible switch ability of the **P_1%_**; **P_2.5%_**; **P_5%_** for 10 cycles with consecutive UV and visible irradiation. Figure S4.^1^H NMR spectra of the **Azo(1)** monomer in MeOD (3.9 mmol/L) indicated the (*E*) isomer (>98%) in non-treated form; enriched (*Z*) isomer mixture (90%) after irradiation with UV light (red) and back switched to (*E*) rich isomer mixture (71%) on irradiation with visible light (blue). Full spectrum of the non-treated sample in Figure S24. Figure S5. DSC scans (10 K/min) from 25 °C to 130 °C: (a) Control PNIPAAm polymer, **P_0%_** (1.418 mg, black); (b) Azo-co-NIPAAm copolymers with increase in mol% of azoacrylate monomer **Azo(1)**: **P_1%_** (1.716 mg, blue); **P_2.5%_** (1.420 mg, orange) and **P_5%_** (1.282 mg, green) show the respective glass transition temperature (T_g_) of the polymers. Figure S6. DSC scans of the control PNIPAAm hydrogel, **H_0%_** at different heating (solid line) cooling rates (dashed line): 10 K/min (green); 15 K/min (orange); 20 K/min (red). Figure S7. Reproducible LCST onset temperature of 20 °C obtained for Azo-co-NIPAAm hydrogels, **H_2.5%_** with a heating rate of 10 K/min for 1, 3 and 6th heating cycles (cycles 2, 4 and 5th represents the same endotherm. Figure S8. DSC scans (10 K/min) comparing the effect on the lower critical solution temperature (LCST) of the control PNIPAAm polymer, **P_0%_**by the increase in the azobenzene content (1 mol%, 2.5 mol%) in the copolymer Azo-*co*-NIPAAm **P_1%_** and **P_2.5%_** respectively. Figure S9. DSC scans (10 K/min) of Azo-*co*-NIPAAm polymer, **P_5%_** with **Azo(1)** content of 5 mol%. Figure S10. DSC scans (1 K/min) of Azo-*co*-NIPAAm polymer, **P_5%_** with **Azo(1)** content of 5 mol%. Figure S11. DSC scans (10 K/min) comparing the effect on the lower critical solution temperature (LCST) of the Control PNIPAAm polymer, **P_0%_** by the increase in the azobenzene content (1, 2.5 mol%) in the hydrogels from Azo-*co*-NIPAAm, **H_1%_** and **H_2.5%_** respectively. Figure S12. DSC scans (1 K/min) of Azo-*co*-NIPAAm hydrogel, **H_5%_** with **Azo(1)** content of 5 mol%. Figure S13. Effect of thermal reversal of the of (Z) character on the non-treated Azo-co-NIPAAm hydrogel, **H_5%_** in the DSC scans at (10 K/min) heating rate: 1^st^ Cycle shows a slight shoulder peak which arises from the 32% (Z) character; 2nd Cycle represents a smooth endotherm reversing the effect due to the previous heating segment. Figure S14. Effect of irradiation time UV (365 nm) on the onset of the lower critical solution temperature of Azo-co-NIPAAm hydrogel, **H_2.5%_**. DSC scans at (10 K/min) heating rate. Figure S15. Effect of irradiation time of vis light (450 nm) on the onset of the lower critical solution temperature of Azo-co-NIPAAm hydrogel, **H_2.5%_**. DSC scans at (10 K/min) heating rate. Figure S16. DSC scans (10 K/min) showing three consecutive cycles on UV (365 nm, 30 min) irradiated Azo-co-NIPAAm hydrogel, **H_2.5%_** indicates the heat induced reversibility on the LCST. Figure S17. DSC scans (10 K/min) comparing the lower critical solution temperature (LCST) of the Control PNIPAAm (**H_0%_**) to the azobenzene (1 mol%) functionalized, Azobenzene-*co*-PNIPAAm hydrogels (**H_1%_**) hydrogels. (a) The onset of the LCST of **H_0%_** in agreement with the literature range between 31 °C for pure PNIPAAm based systems. (b) red: A 5 °C lowering of the LCST in the non-treated **H_1%_** hydrogel relative to **H_0%_**, indicates the effect of incorporating hydrophobic azobenzene in PNIPAAm; purple: A LCST shift in the photochromic **H_1%_** hydrogel with a new endothermic transition temperature, LCST***_H1%-(*Z*)_** = 28 °C on irradiation UV light (λ= 365 nm, 30 min); blue: partial reversing of the LCST by irradiation with blue light (λ= 450 nm, 30 min); light blue: full reversal of the LCST**_H1%-(*E*)_** = 26 °C. Figure S18. DSC scans (1 K/min) of Azo-*co*-NIPAAm hydrogel, **H_5%_** with **Azo(1)** content of 5 mol%; red: non-irradiated; purple: UV (365 nm, 30 min); blue: visible (450 nm, 30 min). Figure S19. GPC plots with calibration of PS polymer standards (380 to 1.8 million) Da, the normalized intensity of RI (green) and UV (red) detector signal vs the retention volume/mL of (a) Control PNIPAAm polymer, **P_0%_** and Azo-*co*-PNIPAAm polymers with varying azo content; (b) **P_1%_**; c) **P_2.5%_**; d) **P_5%_**. Figure S20. GPC elugram with UV detector set at 340 nm with the PNIPAAm polymer as the negative control. Figure S21. ^1^H NMR spectrum of (E)-4-(p-tolyldiazenyl)phenyl acrylate **(s1)** in CDCl_3_. Figure S22. ^13^C{H} NMR spectrum of (E)-4-(p-tolyldiazenyl)phenyl acrylate **(s1)** in CDCl_3_. Figure S23.^13^C{H} NMR spectrum of (*E*)-4-(*p*-tolyldiazenyl)phenyl acrylate, **Azo(1)** in CDCl_3_. Figure S24. ^1^H NMR spectrum of (*E*)-4-(p-tolyldiazenyl)phenyl acrylate, **Azo(1)** in CDCl_3_. Figure S25. ^1^H NMR spectrum of **Azo(1)** at PSS (365nm, 10 min) in CDCl_3_. Figure S26. ^1^H NMR spectrum of **Azo(1)** at PSS (450 nm, 30 min) in CDCl_3_. Figure S27. ^1^H NMR spectrum of **Azo(1)** in MeOD. Figure S28. ^1^H NMR spectrum of Azo-co-NIPAAm Hydrogel, **H_2.5%_** in D_2_O. Figure S29. ^1^H NMR spectrum of the Azo-*co*-NIPAAm Hydrogel, **H_2.5%_** in D_2_O, pre-heated to 50 °C to allow deuterium exchange and visualize the azoacrylate, **Azo(1)** signals. Figure S30.^1^H NMR spectrum of the control PNIPAAm polymer, **P_0%_** in D_2_O Figure S31. ^1^H NMR spectrum of non-irradiated Azo-co-NIPAAm Polymer, **P_2.5%_** in D_2_O. Figure S32. ^1^H NMR spectrum of Azo-*co*-NIPAAm Polymer, **P_2.5%_** in D_2_O at PSS (365 nm, 15 min) in D_2_O. Figure S33. ^1^H NMR spectrum of Azo-*co*-NIPAAm Polymer, **P_2.5%_** at PSS (450 nm, 15 min) in D_2_O. Figure S34. ^1^H NMR spectrum of Azo-*co*-NIPAAm polymer, **P_1%_** in D_2_O with magnified regions of **Azo(1)** aromatic signals under following conditions: top-nontreated; center: UV (365 nm, 15 min); bottom: visible (450 nm, 15 min). Figure S35. ^1^H NMR spectrum of Azo-*co*-NIPAAm polymer, **P_5%_** in D_2_O with magnified aromatic regions of **Azo(1)** under the following conditions: top-nontreated; overlapping and unresolvable signals at center: UV, 365 nm, 15 min and bottom: visible (450 nm, 15 min). Scheme S1. Synthetic scheme to obtain azoacrylate **Azo(1)** functionalized NIPAAm, (Azo-*co*-NIPAAm) polymers via free radical polymerization.
