# Supplementary material for: Controlling the LCST-Phase Transition in Azobenzene-Functionalized Poly (N-Isopropylacrlyamide) Hydrogels by Light
Source: Gels. 2023 Jan 17;9(2):75. doi: 10.3390/gels9020075 (PMC9956105; doi:10.3390/gels9020075)
Supplement: Supplementary file 1 [file gels-09-00075-s001.zip › gels-2128376-supplementary.pdf]

Supplementary Materials

Controlling the LCST-Phase Transition in Azobenzene-Functionalized Poly (*N*-isopropylacrlyamide) Hydrogels by Light

Ruchira Colaco [1,2], Clement Appiah [1,2] and Anne Staubitz [1,2,*]

Contents

1. Abbreviations 1-3
2. Chemicals and Solvents 5
3. Analytical Equipment 7
4. Experimental Section 10
   4.1 Syntheses 10
   4.2 Sample Labeling of the Monomer, Hydrogels and Polymers 10
   4.3 (*E*)-4-(*p*-Tolyldiazenyl)phenol (s1)[1] 11
   4.4 (*E*)-4-(*p*-Tolyldiazenyl)phenyl acrylate (Azo (1))[1] 12
   4.5 Control Poly(*N*-isopropylacrylamide) (PNIPAAm) polymer 14
   4.6 Azoacrylate, Azo(1) functionalized *N*-isopropylacrylamide polymer: poly(Azo-*co*-NIPAAm) 15
5. Hydrogel Reaction Formulations 16
6. Syntheses of Azo-*co*-PNIPAAm-BIS hydrogels: 19
7. Swelling Test of the Hydrogels 20
8. UV-vis Spectroscopy of Monomer, Hydrogel and Polymers 22
   8.1 Monomer: 22
   8.2 Azo-*co*-NIPAAm polymer: 24
9. Switching Tests by NMR Spectroscopy 25
   9.1 ^{1}H NMR of the Azoacrylate Monomer, Azo (1) 25
   9.2 ^{1}H NMR of Hydrogel Azo-*co*-NIPAAm Hydrogels 28
   9.3 ^{1}H NMR of Azobenzene-Functionalized Azo-*co*-NIPAAm Polymers 29
10. Calculation of the Nominal Intensity and Dosage of the UV-vis Irradiation on the Monomer, Polymer and Hydrogel Ssamples 30
11. Differential Scanning Caloriemetry (DSC) of Polymers and Hydrogels 31
   11.1 Determination of the Glass Transition Temperature (T_g) 31

11.2 Optimizing the Heating Rate for Lower Critical Solution Temperature measurements 32
11.3 Cycle Reproducibility 33
11.4 Effect of the Increase in Azo(1) mol% on the Onset Temperature of PNIPAAm Polymer and Hydrogel....34
11.5 Effect of the Increase in Azo(1) mol% on the Onset Temperature of PNIPAAm Hydrogels. 35
11.6 Effect of the Thermal Cycle on the Partial (*Z*) in Non-treated Hydrogel ($H_{2.5\%}$). 38
11.7 Effect of the Irradiation Time to Induce the Reversible LCST Shift....39
11.8 Effect of the Thermal Cycles on the UV Light-induced LCST Shift in $H_{2.5\%}$.41
11.9 Effect of UV and Vis Irradiation on the LCST of $H_{1\%}$ and $H_{5\%}$ Hydrogels..42
12. Gel Permeation Chromatography of the Synthesized PNIPAAm and Azo-*co*-PNIPAAm Polymers....44
13. ^{1}H NMR and NMR Spectra of the Synthesized Compounds....47
References 55

1. Abbreviations

AIBN	Azobisisobutyronitrile
APS	Ammonium persulfate
ATR	Attenuated total reflection
a.u	Arbitrary unit
calcd	Calculated
DCM	Dichloromethane
DMSO	Dimethylsulfoxide
DSC	Differential scanning calorimetry
EI	Electron ionization
EG	End group
GPC	Gel permeation chromatography
HRMS	High resolution mass spectrometry
IR	Infrared
LCST	Lower critical solution transition temperature
MEHQ	Hydroquinone monomethyl ether
M_n	Number average molecular weight
mol%	Mole percent
M_w	Weight average molecular weight
NIPAAm	*N*-isopropylacrylamide
PDI	Polydispersity index
PNIPAAm	Poly *N*-isppropylacrylamide
R_f	Retention factor
RI	Refractive index
TEMED	*N,N,N′,N′*-Tetramethylethylenediamine

T_g	Glass transition temperature
TGA	Thermogravimetric analysis
THF	Tetrahydrofuran
TLC	Thin layer chromatography
UV	Ultraviolet
UV-vis	Ultraviolet-visible
wt %	Weight percent

2. Chemicals and Solvents

All chemicals and solvents were commercially available and were used as received unless noted otherwise in **Table S1**. The dry solvents were either purchased (over molecular sieves with an Acrosseal) or dried with a solvent purification system (SPS), from Inert Innovative Technology, Inc. Company. The inert reactions were carried out using Schlenk techniques under a dry, inert nitrogen atmosphere or in the glovebox form the Inert, Innovative Technology, Inc. Company (0.1 ppm O_2 and 0.1 ppm H_2O).

Table S1. List of supplies and purities of the solvents used.

**Solvent**	**Supplier**	**Purity**
Acetic acid	Grüssing	99.5%
Chloroform-*d*	Euriso-top	99.8% Deuterated
Dichloromethane	VWR	HPLC grade, dry from SPS
Dioxane	Sigma-Aldrich	>99%, dry on Molecular Sieves
Dimethyl sulfoxide	VWR	HPLC grade, dry from SPS
Dimethyl sulfoxide-d_6	Euriso-top	99.8%
Ethanol	Sigma-Aldrich	>99.8%
Ethanol (dry)	Acros Organics	99.5%, extra dry
Ethyl acetate	VWR	99.9%
n-Heptane	AlfaAesar	>99%
n-Hexane	Sigma-Aldrich	>99.9%, HPLC grade
Methanol	Acros Organics	99.9%
Tetrahydrofuran	VWR	HPLC grade

If not noted otherwise, all reagents were used as received (**Table S2**).

Table S2. List of suppliers and purities of the chemicals used.

**Reagent**	**Supplier**	**Purity**	**Comments**
Acryloyl chloride	Sigma-Aldrich	97%	
Ammonium persulfate	Sigma-Aldrich	97%	
Azobisisobutyronitrile	Sigma Aldrich	99%	Recrystallized before use
Hydrochloric acid	Sigma-Aldrich	37%	
Magnesium sulphate	Grüssing	99%	
N-isopropylacrylamide (NIPAAm)	Sigma-Aldrich	98% (stabilized with MEHQ)	Recrystallized before use
N,N′-methylenebis(acrylamide) (BIS) solution	Sigma-Aldrich	2% in H_2O	
N,N′-methylenebis(acrylamide) (BIS) powder	Sigma-Aldrich	99.5%	
N,N,N′,N′-Tetramethylethylenediamine (TEMED)	Acros Ogranics	99.5%	Redistilled with Acros seal
Phenol	Sigma-Aldrich	99%	
Silica gel 60	Merck		
Sodium chloride	Carl Roth	≥99.5%	
Sodium carbonate	Sigma-Aldrich	99%	
Sodium hydroxide	Sigma-Aldrich	≥97%	
Sodium nitrite	Alfa Aesar	98%	
Sodium sulphate	Sigma-Aldrich	>99%	
Triethylamine	Sigma-Aldrich	99%	

3. Analytical Equipment

Gel permeation chromatography (GPC) was performed on a PSS (polymer standard service) SECurity GPC system with a conventional calibration using polystyrene standards (PS). The control and azobenzene functionalized PNIPAAm polymers, poly(Azo-*co*-PNIPAAm) were dissolved in THF (1.5 mg/mL) and GPC traces was recorded. The number average molecular weight (M_n) and the weight average molecular weight (M_w) information obtained was used to calculate the poly dispersity index (PDI) of the synthesized polymers.

Dynamic scanning calorimetry measurements (DSC) were performed on a Mettler Toledo DSC3+ instrument in aluminum crucibles (40 µL, 100 µL), under nitrogen (N_2) at a flow rate of 20 mL/min and heating rates of 10 K/min or 5 K/min.

Thermogravimetric analysis (TGA) was performed on a Mettler Toledo TGA/DSC3+ instrument in aluminum crucibles (100 µL), under nitrogen (N_2) at a flow rate of 20 mL/min.

The UV-vis absorption measurements were recorded at 25 °C on a UV-2700 spectrometer from Shimadzu with a double monochromator. The solvent used for the measurement was HPLC or spectroscopy grade methanol, tetrahydrofuran and deuterated water (D_2O). The irradiation experiments were carried out using UV (365 nm, 1030 mW, nominal intensity = 11.2 mW/cm^2) and blue light (450 nm, 900 mW, nominal intensity = 9.8 mW/cm^2) LEDs. The intensity of the LEDs at longer irradiation time is tabulated in **Table S12**. The absorption of the sample was measured after 30 s or (1, 2 or 30) min of UV and blue light irradiation which was specified in the respective measurement.

Nuclear magnetic resonance spectroscopy (NMR) spectra (^{1}H-NMR, ^{13}C{^{1}H}-NMR) were either recorded on a Bruker DRX 500 at 300 K The NMR spectra were referenced against the solvent residual proton signals (^{1}H) or the solvent itself (^{13}C{^{1}H}).

Mass spectrometric (MS) measurements were performed in the positive ion mode using a JEOL-Accu TOF 4GGCV EI mass spectrometer or on a double focusing mass spectrometer MAT 95+ or Mat 8200 form Finnigan Mat. Electron ionization (EI) was performed using an ionization potential of 70 eV.

IR spectra were recorded with a diamond-ATR-unit spectrometer (iS10) Nicolet by Thermo Scientific. The resolution was 4 cm-1. Relative intensities of the IR bands were assigned as vs = very strong, s = strong, m = middle or w = weak, b = broad.

Freeze drying of hydrogels was performed using the gels swollen in dioxane for ^{1}H-NMR measurements. The gels were flash cooled in liq. N_2 before using a freeze-drying apparatus CHRIST, BETA 2-8 LD plus (-64 at 0.021 mbar) to remove dioxane traces form the hydrogel network.

The samples were analyzed for the swelling and deswelling properties in the temperature range of 5°C to 50°C using the TC1 Temperature Controller from Quantum Northwest. Each sample was equilibrated for 10 min and tracked via the T-App Temperature Control Application at the respective temperature.

The melting temperature of the purified products was recorded on the melting / boiling point apparatus (M-560) by Buchi.

Centrifuge by Hettich (EBA21) was used during the work-up of polymer at 6000 r.p.m to separate the supernatant from the precipitated polymer.

The photographic images of the hydrogels were captured using a Canon EOS 700 D camera, equipped with a zoom lens (18-455 mm). A calibration scale in cm was used in the background of every image captured.

Linkam heating stage (T96 Series) with an end temperature range of -200 to 600 °C was used in combination with the temperature controller unit from Linkam Scientific Instruments Ltd (Model: LNP96-S, serial No: 14102-2805). The hydrogels samples were equilibrated from 5 °C to 50 °C.

4. Experimental Section

4.1 Syntheses

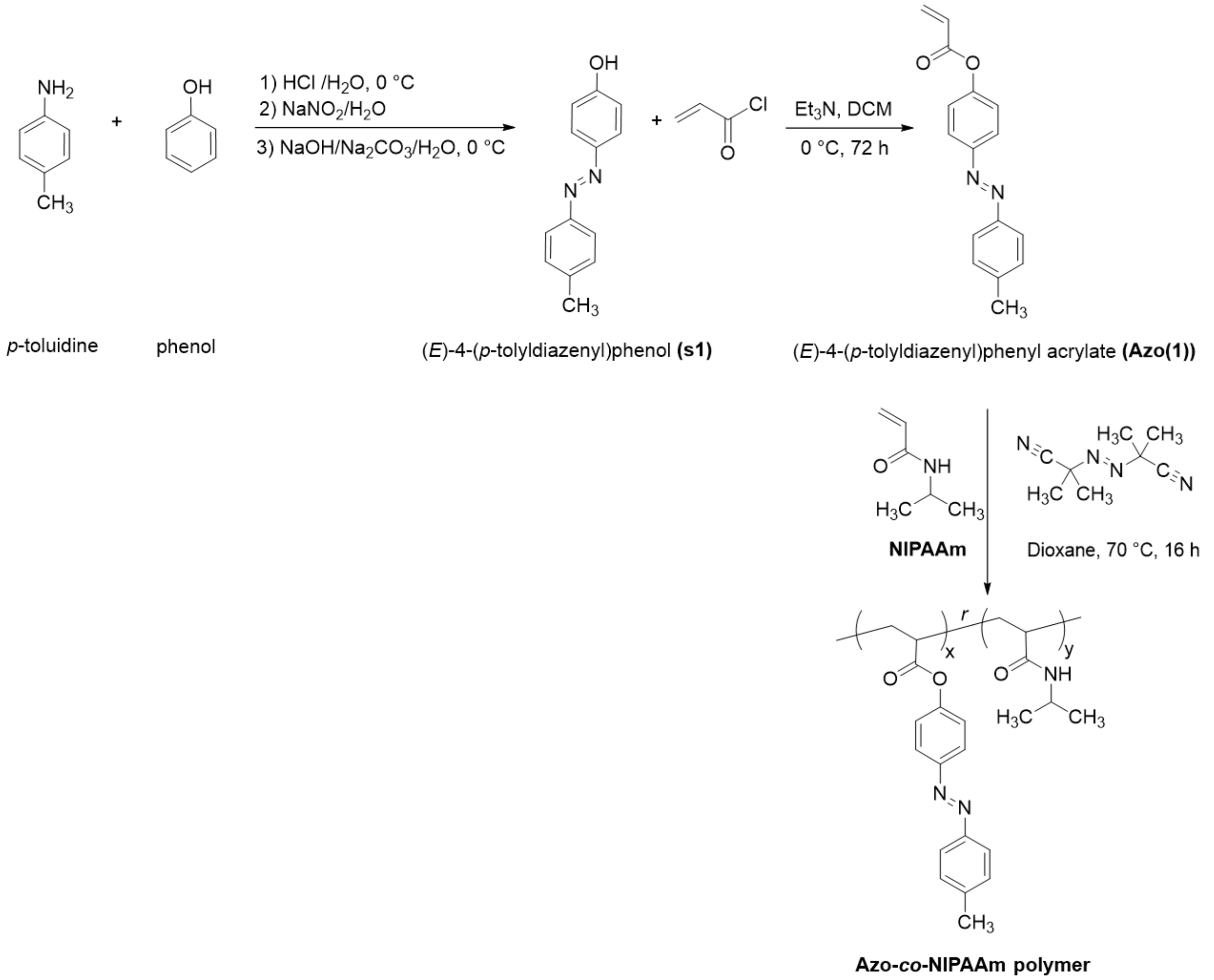

Scheme S1. Synthetic scheme to obtain azoacrylate **Azo(1)** functionalized NIPAAm, (Azo-*co*-NIPAAm) polymers via free radical polymerization.

4.2 Sample Labeling of the Monomer, Hydrogels and Polymers

The azobenzene moiety used for copolymerization with NIPAAm was (*E*)-4-(*p*-tolyldiazenyl)phenyl acrylate **(Azo(1))**. For simplification, the latter is referred to with its common name azoacrylate monomer, **Azo(1)**. The PNIPAM was used as a co-monomer with increase in the azoacrylate monomer, **Azo(1)** content (1, 2.5 and 5) mol%. The labelling of the corresponding linear polymers is $\mathbf{P_{1\%}}$, $\mathbf{P_{2.5\%}}$, $\mathbf{P_{5\%}}$ and of the hydrogels as $\mathbf{H_{1\%}}$, $\mathbf{H_{2.5\%}}$ and $\mathbf{H_{5\%}}$ respectively.

4.3 (*E*)-4-(*p*-Tolyldiazenyl)phenol (s1)[1]

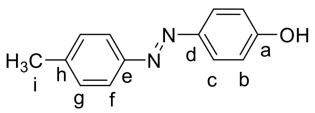

p-Toluidine (2.00 g, 19.0 mmol) was dissolved in a solution of hydrochloric acid in water (2 M, 30 mL) with stirring and the solution was cooled to 0 °C. A solution of sodium nitrite (1.28 g, 19.0 mmol), in water (8 mL) was added slowly to form the diazonium salt. In a separate flask, an aqueous solution of phenol (1.75 g, 19.0 mmol), sodium hydroxide (2.00 g, 50.0 mmol) and sodium carbonate (Na_2CO_3) (5.00 g, 47.5 mmol) were prepared and cooled to 0 °C. The diazonium salt solution was slowly added to the phenolic solution at 0 °C and stirred for 2 h. After neutralization with HCl (2 M, 2 mL) the precipitate was filtered and washed with water (30 mL). The crude substance was dried and purified by recrystallization from ethanol (40 mL) to obtain the compound as a brown solid (2.38 g, 11.2 mmol, 60%, Lit.[1]:75%).[a]

^{1}H NMR (600 MHz, $CDCl_3$, 300K): δ = 7.88 (d, ^3J = 8.8 Hz, 2H, H-c), 7.82 (d, ^3J = 8.3 Hz, 2H, H-f), 7.30 (d, ^3J = 8.3 Hz, 2H, H-g), 6.96 (d, ^3J = 8.8 Hz, 2H, H-b), 5.54 (s, 1H, -*OH*), 2.43 (s, 3H, H-i) ppm.

^{13}C{H} NMR (151 MHz, $CDCl_3$, 300K): δ = 158.3 (C-a), 150.9 (C-e), 147.3 (C-d), 141.1(C-h), 129.9 (C-g), 125.0 (C-c), 122.72 (C-f), 116.0 (C-b), 21.6 (C-i) ppm.

IR (ATR): $\tilde{\upsilon}$ = 3026 (b), 2916 (w), 1601 (m), 1580 (s), 1502 (m), 1460 (w), 1275(s), 1221 (s), 1139 (s), 826 (vs), 721 cm^{-1}.

HRMS (EI): *m/z* (%): [M]$^+$ calcd. for $[C_{13}H_{12}N_2O]^+$ 212.09441; found; 212.09418 (30); 91.1 (100) [M-$C_6H_5N_2O$].

[a] The product **(s1)** was prepared by a modification of synthesis previously reported. The ^{1}H NMR, ^{13}C{H} NMR, data in agreement with the reported. HRMS, IR and melting temperature data were not reported.

R$_f$ = 0.4 (1:1, Cyclohexane:DCM)

Melting temperature: T = 150 °C.

4.4 (*E*)-4-(*p*-Tolyldiazenyl)phenyl acrylate (Azo (1))[1]

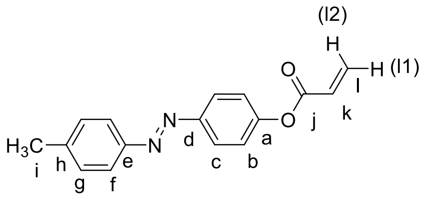

(*E*)-4-(*p*-Tolyldiazenyl)phenol) **(s1)** (500 mg, 2.36 mmol) was dissolved in dry DCM (2 mL) under a nitrogen atmosphere. To the solution, triethylamine (476 mg, 4.71 mmol) was added and the mixture was cooled to 0 °C. Then, acryloyl chloride (213 mg, 2.36 mmol) was diluted in DCM (2 mL) and added dropwise with a syringe pump (0.1 mL/min). After the addition had completed, the solution was stirred at 0°C for 2 h, was allowed to gradually attain 25 °C in the cooling bath and further stirred for 48 h. The crude product was purified by elution on a silica gel column with cyclohexane : DCM (90:10) to obtain the azobenzene-acrylate monomer as orange solid (430 mg, 1.61 mmol, 68%, Lit.[1]: 73%).[b]

^{1}H NMR (601 MHz, $CDCl_3$, 300 K): δ = 7.96 (d, ^3J = 8.9 Hz, 2H, H-c), 7.83 (d, ^3J = 8.3 Hz, 2H, H-f), 7.32 (d, ^3J = 8.3 Hz, 2H, H-g), 7.29 (d, ^4J = 8.9 Hz, 2H, H-b), 6.65 (dd, ^3J = 17.3 Hz, ^3J = 1.1 Hz, 1H, H- l2), 6.35 (dd, ^3J = 17.3 Hz, ^3J = 10.5 Hz, 1H. H-k), 6.06 (dd, J = 10.5, 1.1 Hz, 1H, H-l1), 2.44 (s, 3H, H-i) ppm.

^{1}H NMR (601 MHz, MeOD, 300 K): δ = 7.95 (d, ^3J = 8.9 Hz, 2H, H-c), 7.82 (d, ^3J = 8.3 Hz, 2H, H-f), 7.36 (d, ^3J = 8.3 Hz, 2H, H-g), 7.33 (d, ^4J = 8.9 Hz, 2H, H-b), 6.62 (dd, ^3J = 17.3 Hz, ^3J = 1.1 Hz, 1H, H-l2), 6.41 (dd, ^3J = 17.3 Hz, ^3J = 10.5 Hz, 1H. H-k), 6.11 (dd, J = 10.5, 1.1 Hz, 1H, H-l1), 2.43 (s, 3H, H-i) ppm.

---

[b] The product **(s2)** was prepared by a modification of synthesis previously reported. The ^{1}H NMR, $^{13}C\{H\}$ NMR, HRMS data in agreement with the reported. IR and the melting temperature data were not reported.

^{13}**C{H} NMR** (151 MHz, $CDCl_3$, 300K): δ = 164.2 (C-j), 152.3 (C-a), 150.6 (C-e), 150.3 (C-d), 141.6 (C-h), 133.0(C-l), 129.7 (C-g), 127.7 (C-k), 123.9 (C-c), 122.8(C-f), 122.1(C-b), 21.5 (C-i) ppm.

IR (ATR): $\tilde{\upsilon}$ = 3127 (w), 2915 (w), 1732 (s), 1581 (w),1489 (m), 1403 (m), 1246 (m), 1192 (m), 1142 (m), 1008 (m), 989 (m), 824 (s), 801 (s) cm^{-1}.

HRMS (EI): *m/z* (%): $[M]^+$ calcd. for $[C_{16}H_{14}N_2O_2]^+$ 266.10498; found 266.10473 (25); 91.0 (100) $[M-C_9H_7N_2O_2]^+$.

R_f = 0.5 (50% DCM)

Melting temperature: T = 88°C.

4.5 Control Poly(*N*-isopropylacrylamide) (PNIPAAm) polymer

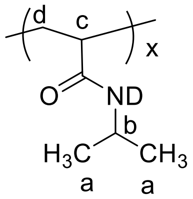

In a vial, *N*-isopropylacrylamide (NIPAAm, 102.5 mg, 906.5 μmol) and azobisisobutyronitrile (10.0 mg, 58.9 μmol, 6.5 mol%) were weighed and transferred into the glovebox. To the reaction vial, dry dioxane (0.75 mL) was added and the solution was stirred for 15 min. Then, the reaction was heated to 70 °C and the polymerization was carried out for 16 h in the glovebox. On completion, the reaction was opened to air and the polymer was purified by precipitation into diethyl ether (50 mL). The mixture was centrifuged for 4 min at 6000 rpm, the supernatant decanted and the precipitated polymer was redissolved in THF (0.75 mL). The purification process was repeated at least 3 times. The polymer was air dried for 24 h followed by heating in the oven at 50 °C for 12 h.

^{1}H NMR (601 MHz, D_2O, 300K): δ = 3.88 (1H, H-b), 1.99 (1H, H-c), 1.55 (2H, H-d), 0.95 (6H, H-a) ppm.

GPC (THF, 1 mL/min): M_n = 12 kDa, M_w = 20 kDa, PDI = M_w/M_n = 1.6.

DSC: T_g = 95 °C.

4.6 Azoacrylate, Azo(1) functionalized *N*-isopropylacrylamide polymer: poly(Azo-*co*-NIPAAm)

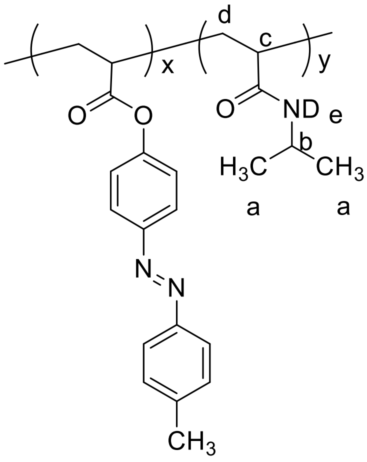

In a vial, *N*-isopropylacrylamide (NIPAAm, 100 mg, 884 µmol), azoacrylate monomer, **Azo(1)** (6.0 mg, 22.5 µmol, 2.5 mol%) and Azobisisobutyronitrile (AIBN, 10.0 mg, 58.9 µmol, 6.5 mol%) were weighed and transferred into the glovebox. To the reaction vial, dry dioxane (0.75 mL) was added and the solution was stirred for 15 min. The reaction was heated to 70 °C and the polymerization was carried out in the glovebox for 16 h. On completion, the reaction was opened to air and the polymer polymer was purified by precipitation into diethyl ether (50 mL). The solution was centrifuged for four minutes at 6000 rpm, the supernatant decanted and the precipitated polymer was redissolved in THF (0.75 mL). The purification process was repeated at least 3 times. The polymer was air dried for 24 h followed by heating in the oven at 50 °C for 12 h.[c]

^{1}H NMR (601 MHz, D_2O, 300K): δ = 3.88 (1H, H-b), 1.99 (1H, H-c), 1.55 (2H, H-d), 0.95 (6H, H-a) ppm.

GPC (THF, 1 mL/min): M_n = 17 kDa, M_w = 32 kDa, PDI = M_w/M_n = 1.9.

DSC: T_g = 97 °C.

[c] The assignment of the ^{1}H NMR shift of the azoacrylate, **Azo(1)** is discussed in the section 7 of the SI.

5. Hydrogel Reaction Formulations

The reaction formulations for the syntheses of hydrogels in the **Table S3**,

Table S*4* and **Table S5** used the *N*,*N*'-methylenenbisacrylamide (BIS) crosslinker commercially available as 2 *w/w*% aqueous solution in water. The control, PNIPAAm hydrogels formulations without the photochrome as a co-monomer, always yielded a crosslinked gel. However, if the azobenzene monomer precipitated out and no gel formation was observed in any formulations, this is marked as 'X'. The respective solutions were degassed with N_2 for 5 min to obtain oxygen free environment. Entry 1-5 are in the solvent system of dioxane and water (4:1). The initiator amount was doubled and the amount of solvent halved for entry 5.

Table S3. Formulations with NIPAAm/Azo : APS/TEMED : 2 w/w% BIS solution (monomers : initiator : crosslinker) systems in a dioxane: water system.

Entry	Azo / mol %	Azo / mg	10 wt% APS sol / µL	2% BIS / µL	TEMED /µL	Solvent system Dioxane : Water (4:1)	Gel Images
**1**	0%	-	50	150	30	1.0 mL	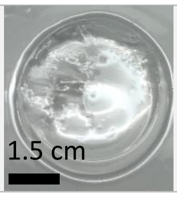
**2**	5%	11.8	50	150	30	1.0 mL	X
**3**	20%	47.2	50	150	30	1.4 mL	X
**4**	0%	-	50	150	10	0.5	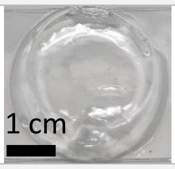
**5**	5%	11.8	110	150	140	0.5	X

(a) The amount of NIPAAm monomer (100 mg) was the same for all the above entries.
(b) All the above reactions were performed at 25 °C for 24 h.
(c) 'X' in the columns indicate no gel formation and the solution remained in liquid state.

Table S4. Formulations with NIPAAm/Azo : APS/TEMED : 2 *w*/*w*% BIS solution in DMSO.

Entry	Azo / mol %	Azo / mg	10 wt% APS / µL	2% BIS / µL	TEMED /µL	Solvent system DMSO	Gel Images
**1**	0%	-	50	150	75	0.9	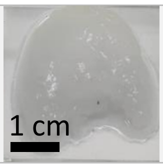
**2**	2.5%	6	50 / 100	150	75	0.9	X

(a) The amount of NIPAAm monomer (100 mg) was the same for all the above entries.
(b) All the above reactions were performed at 25 °C for 24 h.
(c) 'X' in the columns indicate no gel formation and the solution remained in liquid state.

Table S5. Formulations with NIPAAMm/Azo : AIBN : 2 *w*/*w*% BIS Monomers : thermal initiator : crosslinker) in DMSO.

Entry	Azo(1) / Mol %	Azo(1) / mg	AIBN / mg	2% BIS / µL	Solvent / mL	Gel Images
**1**	0%	-	5.6	150	0.75	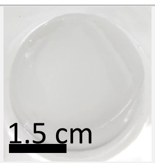
**2**	2.5%	6.0	5.6	150	0.75	X

(a) The amount of NIPAAm monomer (100 mg) was the same for all the above entries.
(b) All the above reactions with the thermal initiator AIBN were performed at 50 °C for 6 h.
(c) X' in the columns indicate no gel formation and the solution remained in liquid state.

The experiments above revealed, that no gel formation took place when the BIS crosslinker in form of aqueous solution (2 *w*/*w*%) in water was used. Water free reaction conditions were necessary for successful co-polymerization and crosslinking of the hydrogels with **Azo(1)**. The crosslinker available as a solid colorless powder was thus used for all further formulations, keeping mol%

constant. To evaluate the effect of azobenzene on the LCST of the control PNIPAAm hydrogel, the mol% of azobenzene was increased (1%, 2.5%, 5%), while keeping the total moles of the two monomers constant. The total ratio of the two monomers (NIPAAm and **Azo(1)** to the initiator was varied from 100:2 to 100:5 until stable hydrogels with highest mol% of Azo were obtained (

Table S6). The reaction time and temperature were increased to 16 h and 70°C. The formulation with 5 mol% crosslinker yielded a soft gel only for one reaction mixture (entry 1).

Table S6. Formulations with NIPAAM/Azo : AIBN : solid BIS (Monomers : initiator : crosslinker) in 1,4-dioxane.

**Entry**	**Azo(1) / Mol %**	**Azo(1) / mg**	**AIBN / mg**	**BIS / mg**	**Solvent / mL**	**Comments**
Monomers:initator:crosslinker (100 : 2 : 5)						
**1**	2.5%	6.0	97.5	2.90	6.81	Gel
**2**	5.0%	12.0	95.0	2.90	6.81	X
**3**	10.0%	24.0	90.0	2.90	6.81	X
Monomers:initator:crosslinker (100 : 5 : 5)						
**4**	5.0%	12.0	95.0	7.30	6.81	X
**5**	10.0%	24.0	90.0	7.30	6.81	X

(a) The amount of NIPAAm monomer (100 mg) was the same for all the above entries.
(b) All the above reactions with the thermal initiator AIBN were performed at 70 °C for 16 h.

6. Syntheses of Azo-*co*-PNIPAAm-BIS hydrogels:

To prepare the azobenezene functionalized PNIPAAm hydrogels by free-radical polymerization, azoacrylate (**Azo(1)**, 6.0 mg, 22.5 µmol), *N*-isopropylacrylamide (NIPAAM, 100 mg, 884 µmol), azobisisobutyronitrile (AIBN, 10.0 mg, 58.9 µmol, 6.5 mol%) as the thermal initiator and *N*, *N*′-methylenenbisacrylamide (BIS, 9.0 mg, 58.9 µmol, 6.5 mol%) as crosslinker were used. The monomers, crosslinker and the initiator were weighed in vials and transferred into the glovebox to ensure an oxygen-free atmosphere. To the vials, dry 1,4-dioxane (0.75 mL) was added and the solution was stirred for 15 min to ensure homogeneous mixing. From the solution, 200 µL was transferred with a microliter syringe into glass molds of 8.8 mm diameter, sealed and heated to 70 °C for 16 h. The crosslinked gels were removed from the glovebox and were immersed in methanol, which was refreshed several times for least one week to remove the unreacted monomers and 1,4-dioxane. The gels were then air dried for 24 h, followed by drying in the vacuum oven at 60 °C for 48 h. The weight of the dry gel was recorded for further analyses. The dried gels were re-swollen in distilled water and used for Differential Scanning Calorimetry (DSC) analyses. The total moles of the solutions were kept constant at 906.5 µmol in 0.75 mL of dioxane. The total ratio of the two monomers (NIPAAm and **Azo(1)**) combine, to the initiator and crosslinker was optimized to 100:6.5:6.5 respectively. The amount of **Azo(1)** was increased as (1, 2.5, 5) mol% and the three corresponding hydrogel systems ($\mathbf{H_{1\%}}$, $\mathbf{H_{2.5\%}}$ and $\mathbf{H_{5\%}}$) were obtained. Using the same protocol, Control PNIPAAm hydrogel ($\mathbf{H_{0\%}}$) without azoacrylate monomer was prepared and used as a reference for DSC characterization. The reaction scheme is given in the manuscript (**Scheme 1**).

7. Swelling Test of the Hydrogels

The swelling ratio W_s(%) is the measure of the amount of water retained in the network when equilibrated at a particular temperature. The mass of the pre-dried hydrogel sample at 50 °C for 48 h was noted and immersed in deionized water for at least 24 h to attain full equilibrium at 25 °C and 5 °C. The sample was removed and dabbed with a wet tissue to remove only the excess surface water and was weighed accurately on a precision balance. At least six to eight samples were used during the swelling test and any sample that cracked was discarded from the series. At least four samples were used to calculate the mean and standard deviation to obtain the swelling using the formula below:

$$W_s(\%) = \frac{m_w - m_d}{m_w} X\ 100\% \quad (1)$$

W_s is the swelling ratio of the hydrogels at 24 h, m_d is the initial dry weight before submersion, and m_w is the weight after 24 h hydration time at 25 °C and 5 °C respectively.

Table S7. Swelling ratio in % for the control and functional hydrogels after equilibrating for 24 h at 25 °C.

Hydrogels @ 25 °C	S1	S2	S3	S4	Mean ± Standard deviation
**$H_{0\%}$**	91	91	91	90	90.7 ± 0.5
**$H_{1\%}$**	89	88	88	89	88.5 ± 0.6
**$H_{2.5\%}$**	70	69	70	70	69.7 ± 0.5
**$H_{5\%}$**	35	32	35	27	32.2 ± 3.7

Table S8. Swelling ratio in % for the control and functional hydrogels after equilibrating for 24 h at 5 °C.

Hydrogels @ 5 °C	S1	S2	S3	S4	Mean ± Standard deviation
**$H_{0\%}$**	94	94	93	93	93.5 ± 0.6
**$H_{1\%}$**	92	93	92	94	92.5 ± 0.6
**$H_{2.5\%}$**	89	87	88	88	88 ± 0.8
**$H_{5\%}$**	81	82	83	80	81.5 ± 1.2

8. UV-vis Spectroscopy of Monomer, Hydrogel and Polymers

The absorption maxima and the photostationary state (PSS) for the azoacrylate monomer **Azo(1)**, Azo-*co*-NIPAAm hydrogel, **H$_{2.5\%}$** and the corresponding polymers (**P$_{1\%}$**, **P$_{2.5\%}$**, **P$_{5\%}$**) were determined via UV-vis spectroscopy. The aforementioned samples were irradiated with UV and visible light for (0 s, 30 s, 1 min, 2 min) to investigate the photostationary state (PSS) of (*Z*) and (*E*) isomer respectively. The thermal relaxation of the (*Z*) to (*E*) isomer was monitored for 16 h at 25 °C by recording a spectra at an interval of every 30 min. The absorption maximum at the respective $\lambda_{max}(Z)$ were plotted against time to estimate the half -life ($t_{1/2}$) given by the equation:

$$t_{1/2} = \frac{\ln 2}{k} \quad (2)$$

8.1 Monomer:

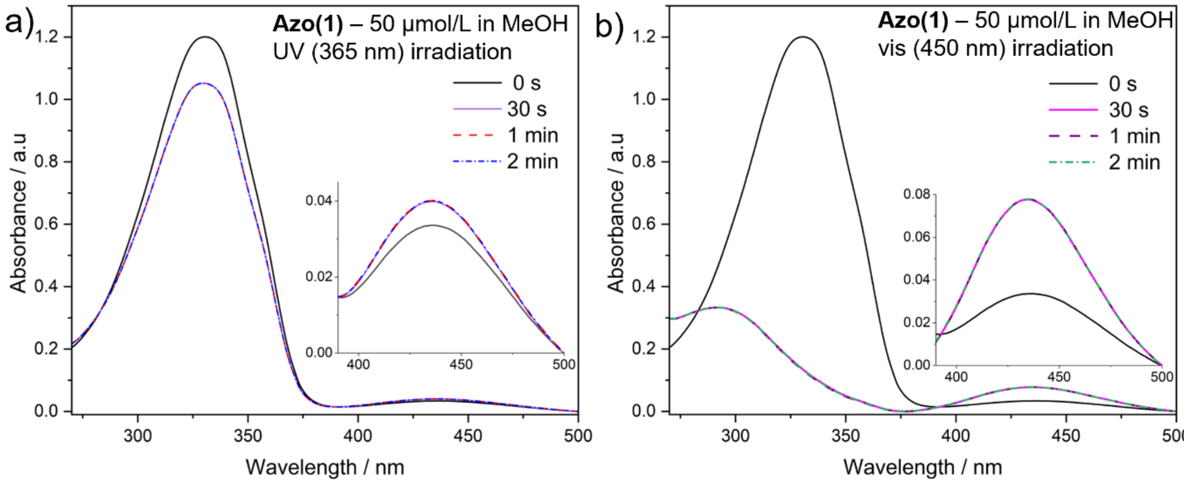

Figure S1.UV-vis absorption spectra of the azoacrylate monomer, **Azo(1)** in methanol (50 µmol) with varying irradiation time (0 s, 30 s, 1 min, 2 min) to reach the photostationary state (PSS): a) UV irradiation (365 nm, nominal intensity = 11.2 mW/cm^2) and b) vis (450 nm, nominal intensity = 9.8 mW/cm^2). The inset figure has the same X and Y-axis as the main plot.

Solution of the monomer **Azo(1)** (50 µmol in methanol) was irradiated with UV light (365 nm, 1 min) to monitor the thermal relaxation at (λ_{max} = 434 nm). The half-life of $t_{1/2}$ = 1.7 days (**Figure S2**).

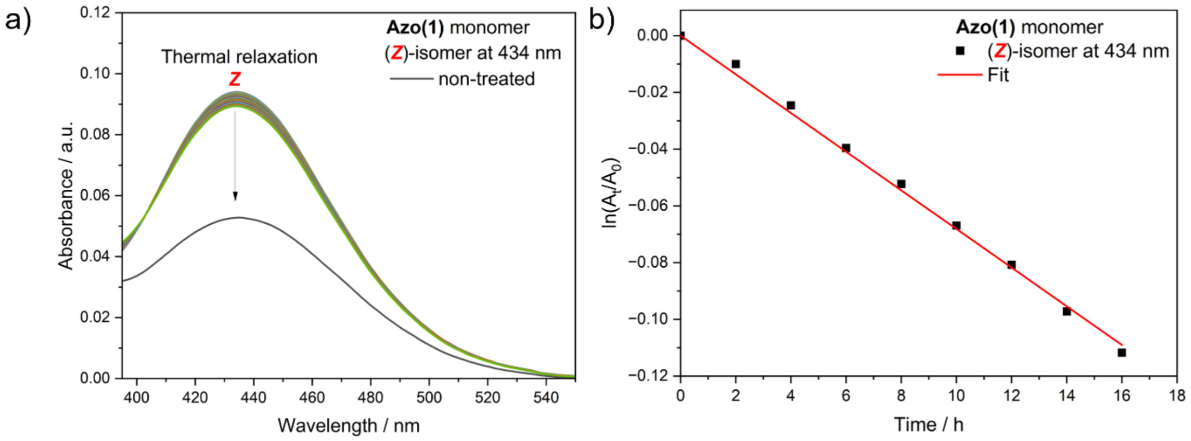

Figure S2. UV-vis absorption spectra of the azoacrylate monomer, **Azo(1)** in methanol (50 µmol): a) Band of spectra after irradiating the solution to PSS (*Z*)-isomer with UV light, λ = 365 nm, for 1 min and recorded at an interval of 30 min b) First-order thermal relaxation kinetics of **Azo(1)** from PSS (450 nm) at λ_{max} = 434 nm.

8.2 Azo-*co*-NIPAAm polymer:

In order to investigate the photoinduced influence of **Azo(1)** on PNIPAAm polymer chains, the polymer solutions of **P1%**, **P5%** were prepared in D_2O. The switchability of the azo moiety in an aqueous when covalently linked to a PNIPAAm polymer (**Figure S3**) was investigated by UV-vis spectroscopy.

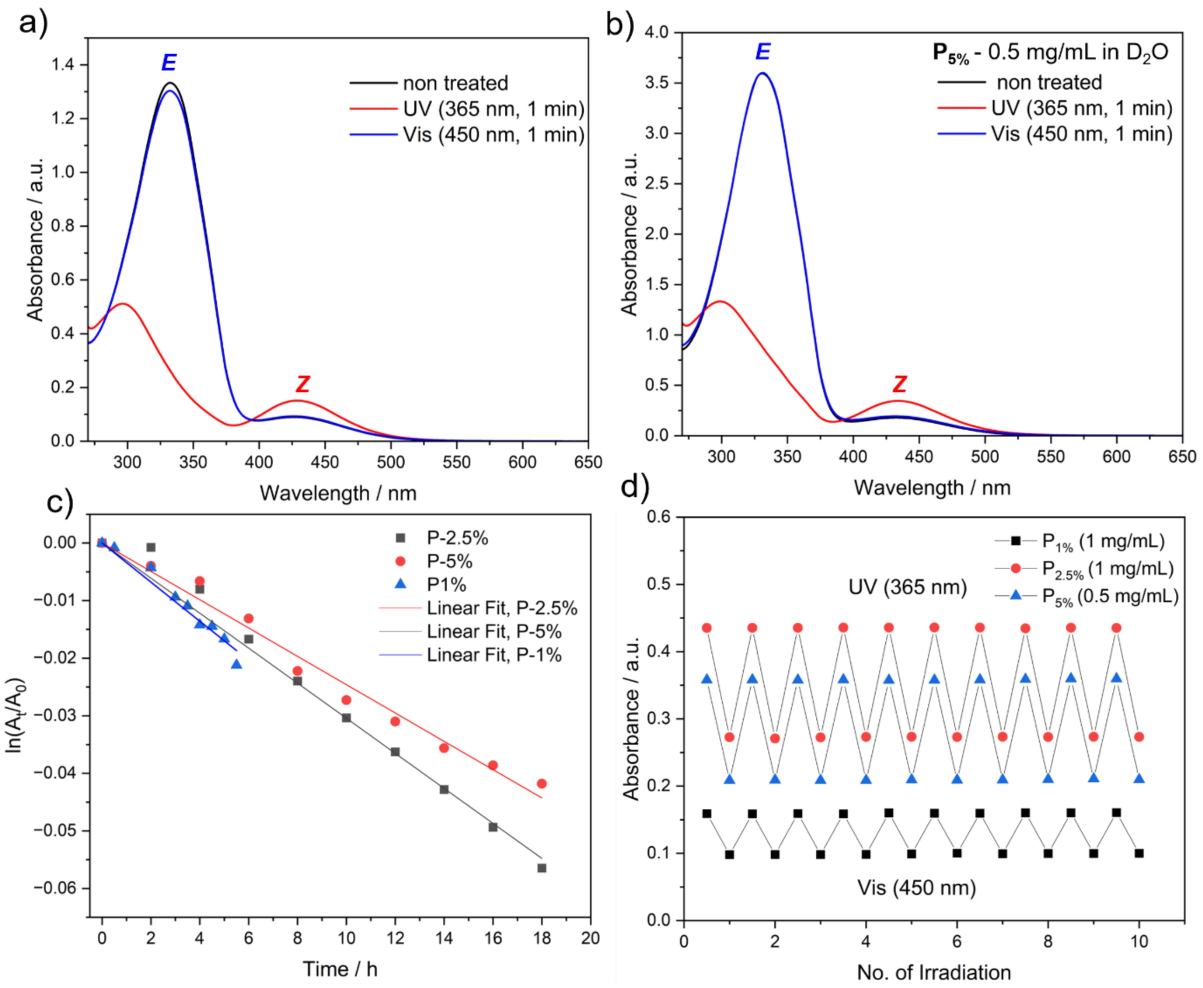

Figure S3. UV-vis absorption spectra of Azo-*co*-NIPAAm polymers in D_2O indicating reversible switching when irradiated with UV light (365 nm, 1 min); and vis light (450 nm, 1 min) for a) **P1%**; b) **P5%**; c) The first-order thermal relaxation kinetics at PSS (450 nm) for **P1%**; **P2.5%**; **P5%** and reversible switch ability of the **P1%**; **P2.5%**; **P5%** for 10 cycles with consecutive UV and visible irradiation.

9. Switching Tests by NMR Spectroscopy

9.1 ^{1}H NMR of the Azoacrylate Monomer, Azo (1)

The azoacrylate monomer **Azo(1)** switches between the (*Z*) and the (*E*) form on irradiation with 365 nm and 450 nm wavelength respectively. The effect of irradiation time (10, 15, 30 and 40 min), leading to the photostationary state (PSS) in MeOD and $CDCl_3$ are given in

Table S9 and **Table S10** respectively. In MeOD, the signals for the thermodynamically stable (*E*) isomer were observed in the downfield region as multiplet peak, ***a*** (7.34 ppm) and set of two doublets, ***b*** (7.83 ppm) and ***c*** (7.96 ppm) respectively. On UV irradiation for 10 min, the (*E*) isomer switched to 90% of the (*Z*) isomer. The enriched peaks denoting the (*Z*) isomer showed a distinct mulitplet ***a'*** (7.10 ppm) and the two doublets, ***b'*** (6.79 ppm) and ***c'*** (6.91 ppm) respectively. In addition to this, a pattern of two doublets of the (*E*) isomer as side peaks also appeared next to the main ***b*** and ***c*** peaks. On back irradiation with the visible light, 71% enrichment in the (*E*) isomer was obtained (see SI, **Figure S4**).

Table S9. Irradiation of the azoacrylate monomer, **Azo(1)** to determine the percentage of **(*E*)** and **(*Z*)**-isomer from ^{1}H NMR in MeOD respectively.

Sample	(*E*)%	(*Z*)%	Conditions
**Azo(1)-**monomer in MeOD	100	0	Non treated
	10	90	**PSS 365 nm-10 min**
	71	29	450 nm-10 min
	77	23	**PSS 450 nm-30 min**

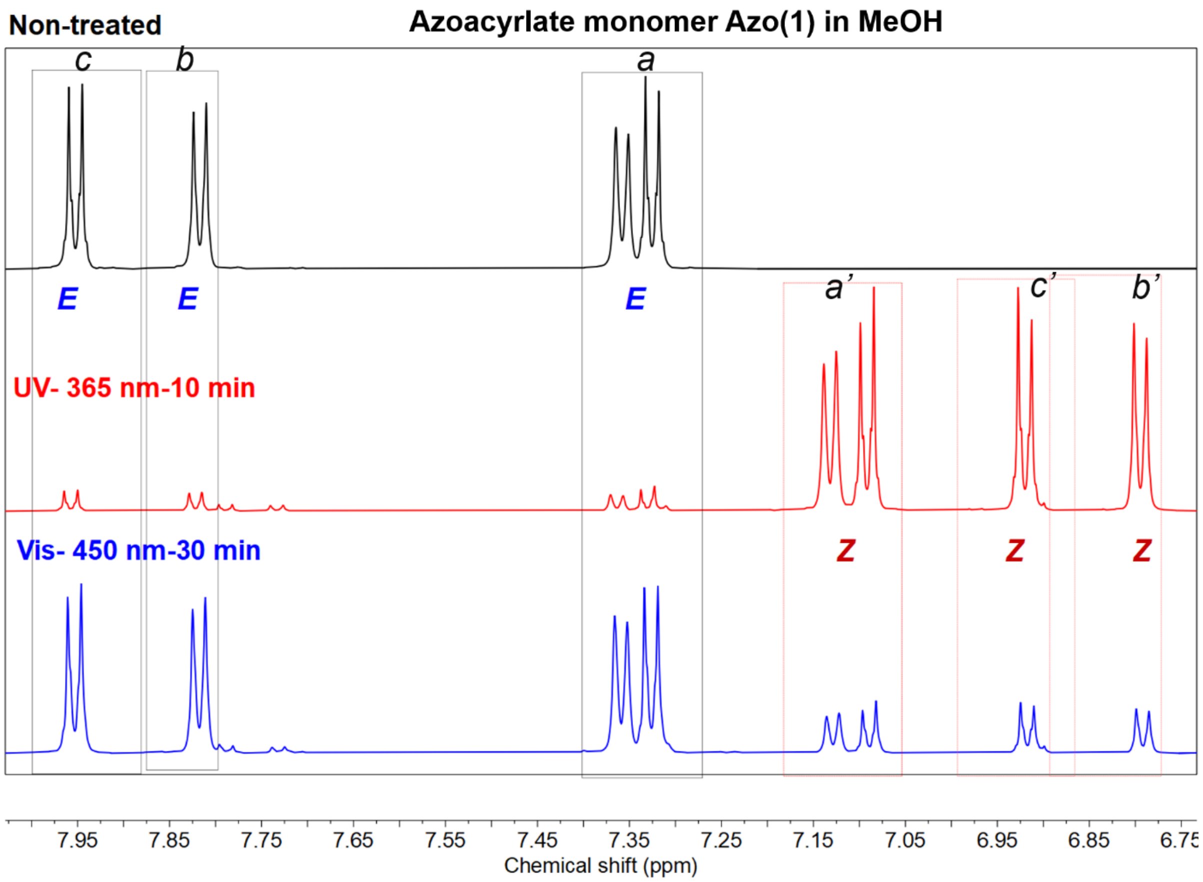

Figure S4. ^{1}H NMR spectra of the **Azo(1)** monomer in MeOD (3.9 mmol/L) indicated the (*E*) isomer (>98%) in non-treated form; enriched (*Z*) isomer mixture (90%) after irradiation with UV light (red) and back switched to (*E*) rich isomer mixture (71%) on irradiation with visible light (blue). Full spectrum of the non-treated sample in **Figure. S24**.

In $CDCl_3$, on irradiation with 365 nm for 10 min, the PSS was attained giving 93% of the (*Z*) form. Further irradiation did not enrich the mixture in the (*Z*) isomer. The back switching with 450 nm required a longer irradiation time of 30 min to attain equilibrium of 80% (*E*) with 20% of (*Z*) isomer. The integration of signals at PSS (365 nm and 450 nm) were carried out after phase and baseline correction and the full spectra are given in (See SI, **Figure 19-20**).

Table S10. Irradiation of the azoacrylate monomer, **Azo(1)** to determine the percentage of **(*E*)** and **(*Z*)**-isomer from ^{1}H NMR in $CDCl_3$ respectively.

**Sample**	**(*E*)%**	**(*Z*)%**	**Conditions**
**Azo(1)-**monomer in $CDCl_3$	100	0	Non treated
	7	93	**PSS 365 nm-10 min**
	63	37	450 nm-10 min
	80	20	**PSS 450 nm-30 min**

9.2 ^{1}H NMR of Hydrogel Azo-*co*-NIPAAm Hydrogels

To record the ^{1}H NMR spectrum of the swollen hydrogel, from the stock solution to prepare hydrogel, (0.4 mL) was transferred into NMR tube, sealed and the reaction was carried out at 70°C for 16 h in the glovebox. After completion, the tube was removed and excess of dioxane was added (three times) to wash out the unreacted monomers. The layer of dioxane was decanted and the tube was supercooled in liq. N_2 for 1 min. The hydrogel was dried at the lyophilizer for 24 h. The dried hydrogel was reswollen for 24 h in D2O to record the 1H NMR (600 MHz, 80 scans) which gave broad signals throughout (see Section 13). The -NH peak of the amide functional group in the control PNIPAAm chain was observed in the aromatic region, as a broad feature, overlapping the **Azo(1)** signals. The sample in the NMR tube was heated to accelerate the deuterium exchange (-NH to -ND). Heating the tube sample to 50 °C for 10 min resulted in the exchange and eliminated the -NH signal. However, due to the high signal to noise ratio, the **Azo(1)** signals corresponding to the (*E*) and (*Z*) isomer could not be resolved to determine the isomer ratio. Therefore, for quantifying the (*Z*) and the (*E*) isomer percentage, ^{1}H NMR spectra of the corresponding azoacrylate **Azo(1)** functionalized NIPAAm, Azo-co-NIPAAm polymers were recorded as the closest possible comparison.

9.3 ^{1}H NMR of Azobenzene-Functionalized Azo-*co*-NIPAAm Polymers

The ^{1}H NMR spectra of the azobenzene-functionalized Azo-*co*-NIPAAm polymers (**$P_{1\%}$, $P_{2.5\%}$, $P_{5\%}$**) with an increasing amount of **Azo(1)** content was recorded in D_2O. The polymer was reversibly switched with 365 nm and 450 nm for (15 and 30) min respectively. The PSS was reached with 15 min of irradiation time, given in (**Table S11**). As extremely weak signals were integrated post manual phase and baseline correction, we expect an error of 7 to 10% in the switching integrals tabulated below.

Table S11. Irradiation of the azobenzene-functionalized Azo-*co*-NIPAAm Polymers (**$P_{1\%}$, $P_{2.5\%}$, $P_{5\%}$**) to determine percentage of (*E*) and (*Z*)-isomer from ^{1}H NMR spectroscopy in D_2O.

Polymer sample	(*E*) %	(*Z*) %	Conditions in D_2O
**$P_{1\%}$,**	80%	20%	Non treated
	14%	86%	**PSS 365 nm-15 min**
	75%	25%	**PSS 450 nm-15 min**
**$P_{2.5\%}$**	68%	32%	Non treated
	4%	96%	**PSS 365 nm-15 min**
	75%	25%	**PSS 450 nm-15 min**
**$P_{5\%}$**	77%	23%	Non treated
	Overlapping signals	Overlapping signals	**365 nm-15 min**
	N/A	N/A	**450 nm-15 min**

10. Calculation of the Nominal Intensity and Dosage of the UV-vis Irradiation on the Monomer, Polymer and Hydrogel Ssamples

The lamps used for irradiation were UV (365 nm, 1030 mW, nominal intensity = 11.2 mW/cm^2) and blue light (450 nm, 900 mW, intensity = 9.8 mW/cm^2). The lamp fitted inside the holder above the sample at a distance of 2.7 cm. The spot size of the lamps with 365 nm and 450 nm wavelength has a radius r = 1.6 cm. The radius of the circular opening of the quartz lid was 0.3 cm. The intensity of the dosage is calculated as follows: The sample distance from the lamp in meters, which is the radius of sphere around the lamp was used to calculate the area of the sphere, $4(\pi)r^2$. The power of the lamp in Watt (W) was divided by the area to get the intensity in W/m^2 or can be converted to mW/cm^2. Exposure time which was the duration of the UV or the vis light delivering the radiation to the surface was used to further calculate the UV dosage as:

UV Dose = UV Intensity (W/cm^2) x Exposure Time (seconds)

The result of this calculation is expressed in = W s/cm^2 or J/cm^2 and tabulated for the various irradiation time in (**Table S12**).

Table S12. UV and Vis dosage during irradiation experiment of the hydrogels at the DSC.

Time / min	Time / s	UV dosage in J / cm^2	Blue light dosage in J / cm^2
1 min	60	0.7	0.6
2 min	120	1.4	1.2
15 min	900	10.8	8.8
30 min	1800	20.2	17.6
1 h	3600	40.3	35.3

11. Differential Scanning Caloriemetry (DSC) of Polymers and Hydrogels

11.1 Determination of the Glass Transition Temperature (T_g)

The glass transition temperature of the control and the functional polymers recorded via DSC, 20 °C to 130 °C are given below (Figure S5).

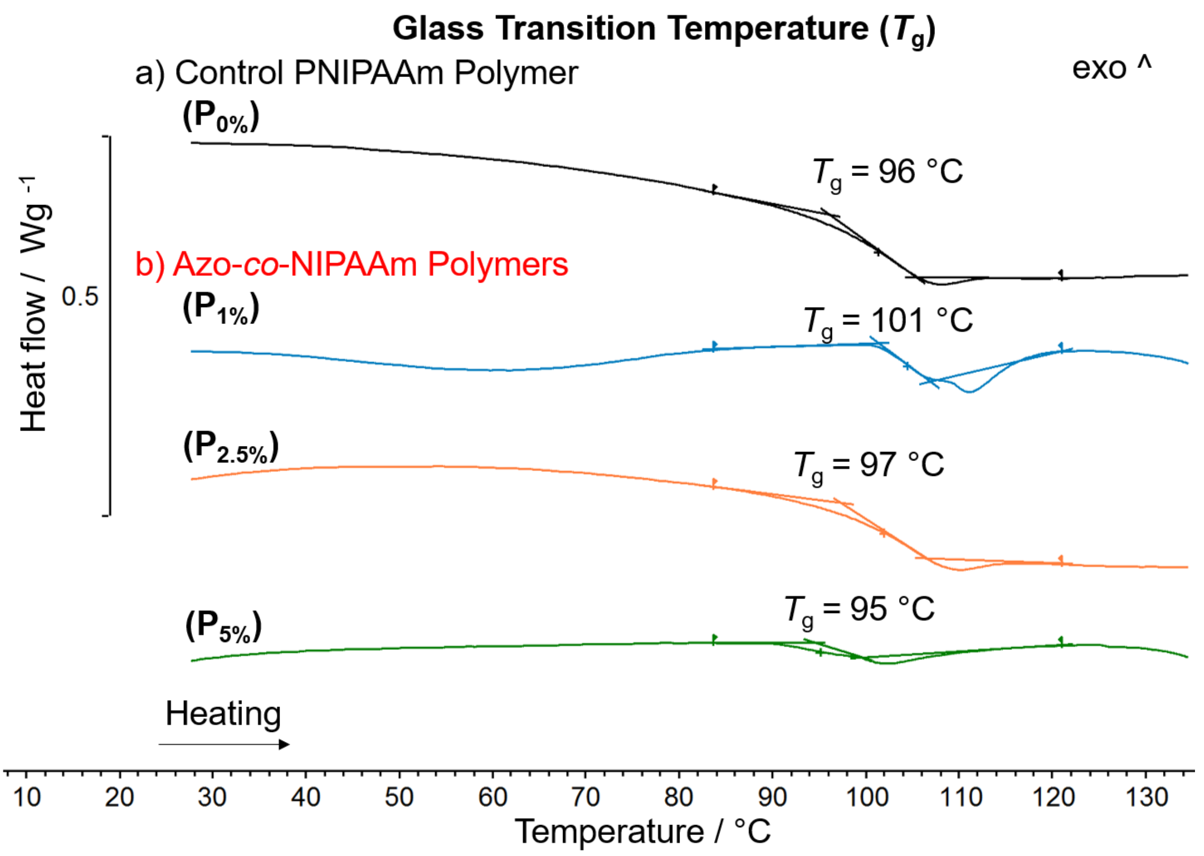

Figure S5. DSC scans (10 K/min) from 25 °C to 130 °C: a) Control PNIPAAm polymer, $\mathbf{P_{0\%}}$ (1.418 mg, black); b) Azo-*co*-NIPAAm copolymers with increase in mol% of azoacrylate monomer **Azo(1)**: $\mathbf{P_{1\%}}$ (1.716 mg, blue); $\mathbf{P_{2.5\%}}$ (1.420 mg, orange) and $\mathbf{P_{5\%}}$ (1.282 mg, green) show the respective glass transition temperature (T_g) of the polymers.

11.2 Optimizing the Heating Rate for Lower Critical Solution Temperature measurements

The effect of the heating rate on the endothermic and exothermic features via DSC can be seen in (**Figure S6**) for the control hydrogel $H_{0\%}$.

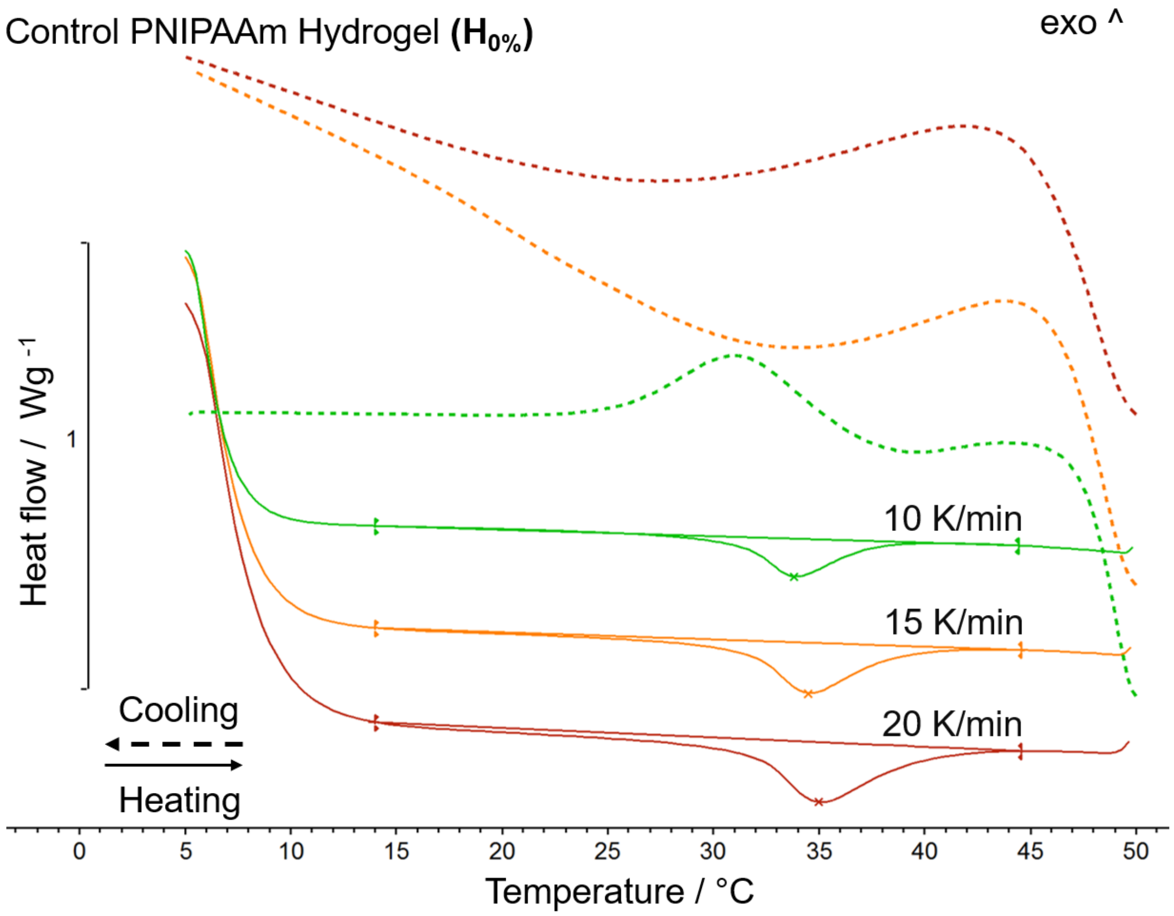

Figure S6. DSC scans of the control PNIPAAm hydrogel, $\mathbf{H_{0\%}}$ at different heating (solid line) cooling rates (dashed line): 10 K/min (green); 15 K/min (orange); 20 K/min (red).

11.3 Cycle Reproducibility

The cycle reproducibility of the endothermic, LCST feature for the heating rate of 10 K/min can be seen in (**Figure S7**).

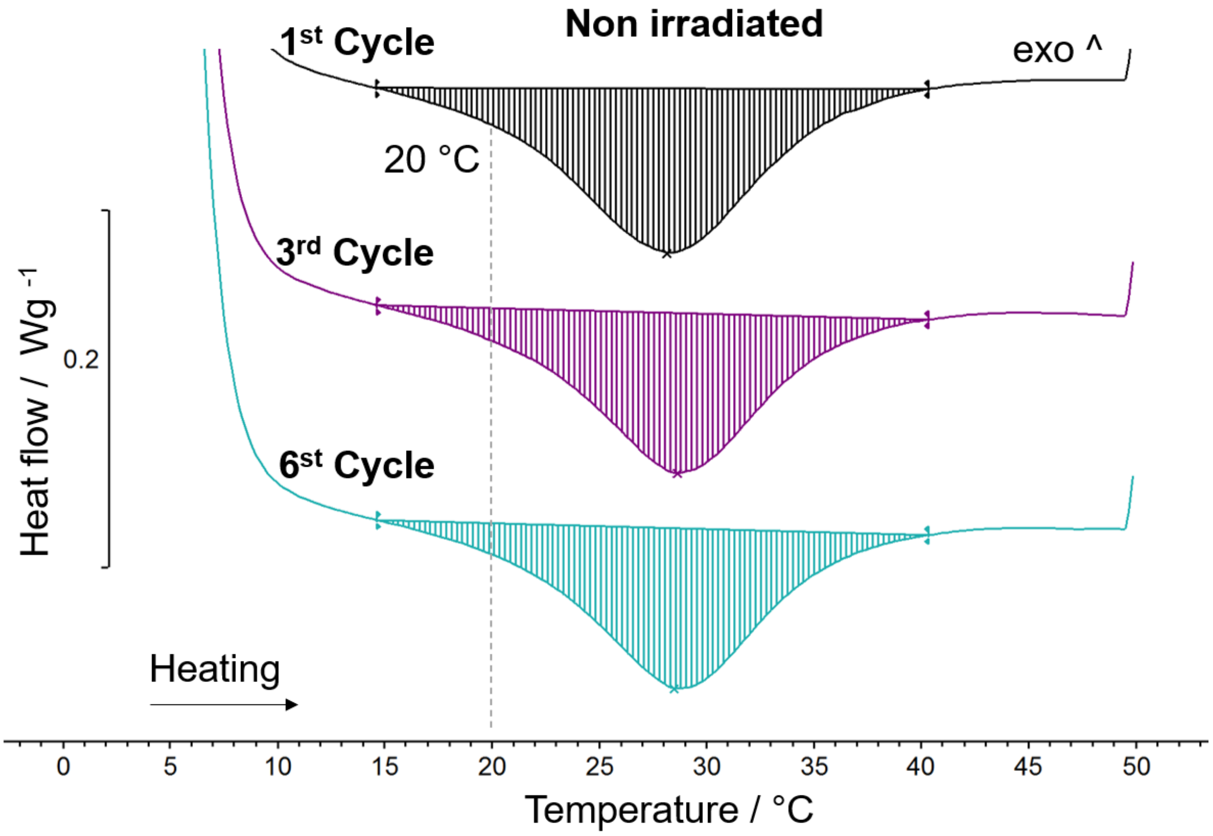

Figure S7. Reproducible LCST onset temperature of 20 °C obtained for Azo-*co*-NIPAAm hydrogels, $\mathbf{H}_{2.5\%}$ with a heating rate of 10 K/min for 1, 3 and 6th heating cycles (cycles 2, 4 and 5th represents the same endotherm.

11.4 Effect of the Increase in Azo(1) mol% on the Onset Temperature of PNIPAAm Polymer and Hydrogel

The onset of the LCST in the linear, control **P0%**, and functional polymers (**P1%**, **P2.5%**) with a heating rate of 10 K/min is shown in (**Figure S8**) and (**Figure S9**) for **P5%** respectively. The heating rate of 10 K/min did not allow to distinguish a clear onset of the LCST in this case. Therefore, the heating rate for the **P5%** was lowered to 1 K/min in order to obtain a baseline separated signal (**Figure S10**). At least three samples were measured and the standard deviation calculated for the LCST onset values.

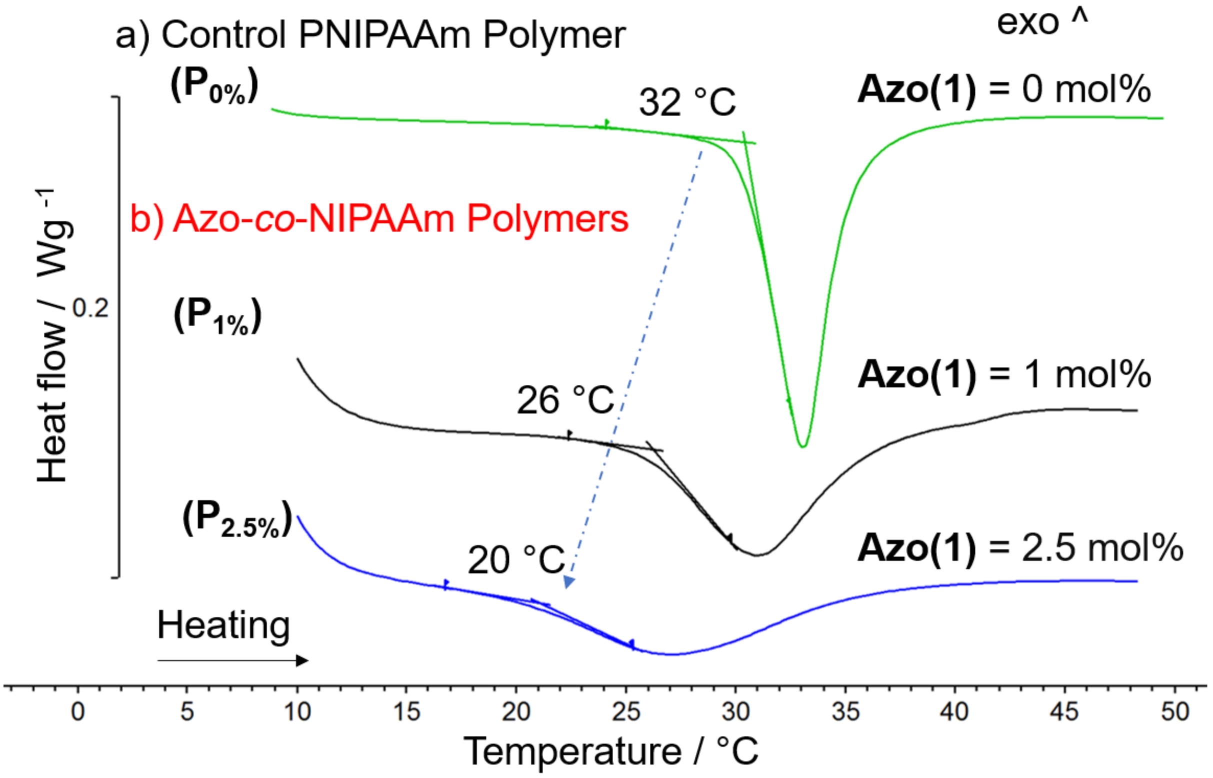

Figure S8. DSC scans (10 K/min) comparing the effect on the lower critical solution temperature (LCST) of the control PNIPAAm polymer, **P0%** by the increase in the azobenzene content (1 mol%, 2.5 mol%) in the copolymer Azo-*co*-NIPAAm **P1%** and **P2.5%** respectively.

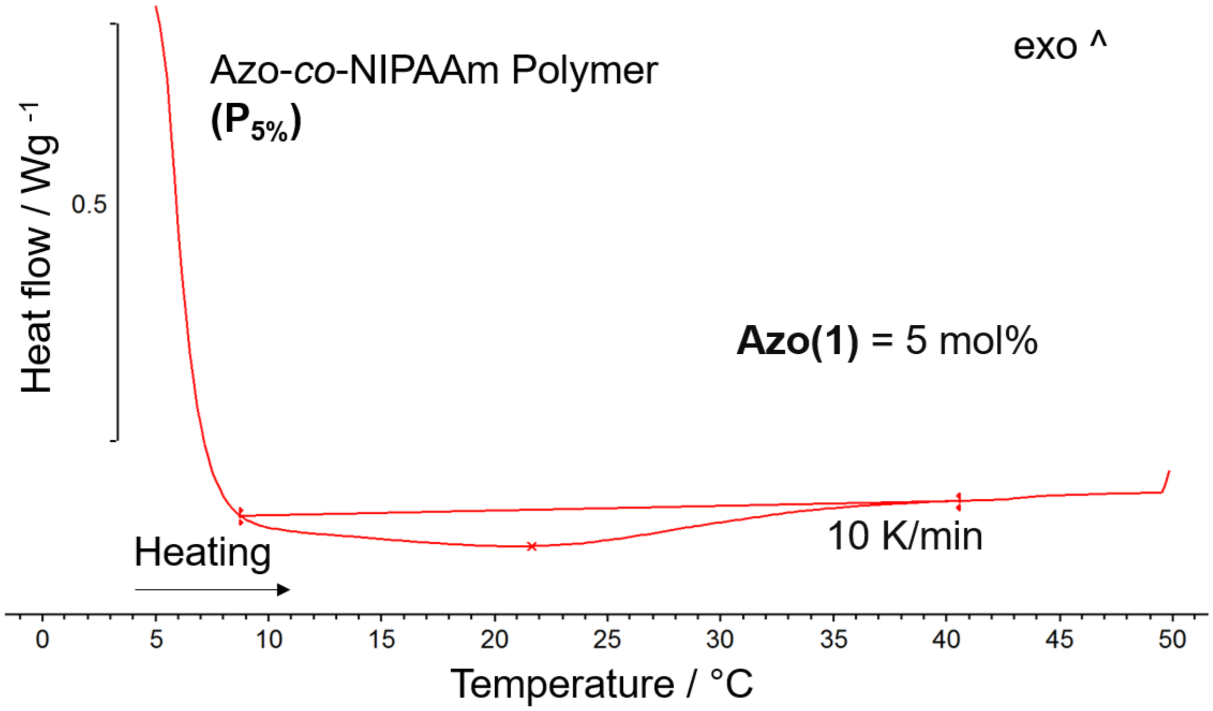

Figure S9. DSC scans (10 K/min) of Azo-*co*-NIPAAm polymer, $\mathbf{P_{5\%}}$ with **Azo(1)** content of 5 mol%.

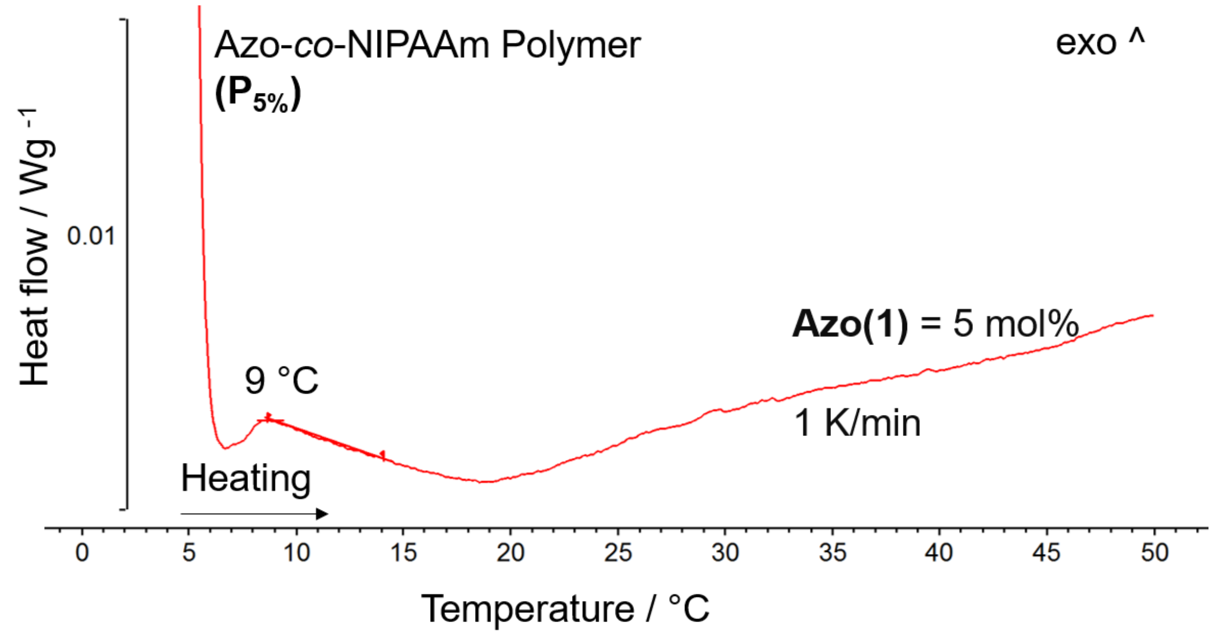

Figure S10. DSC scans (1 K/min) of Azo-*co*-NIPAAm polymer, $\mathbf{P_{5\%}}$ with **Azo(1)** content of 5 mol%.

11.5 Effect of the Increase in Azo(1) mol% on the Onset Temperature of PNIPAAm Hydrogels.

The onset of the LCST in the crosslinked, control $\mathbf{H_{0\%}}$ and functional hydrogels ($\mathbf{H_{1\%}}$, $\mathbf{H_{2.5\%}}$) with a heating rate of 10 K/min is shown in (**Figure S11**) and 1°K/min for $\mathbf{H_{5\%}}$ (**Figure S12**).

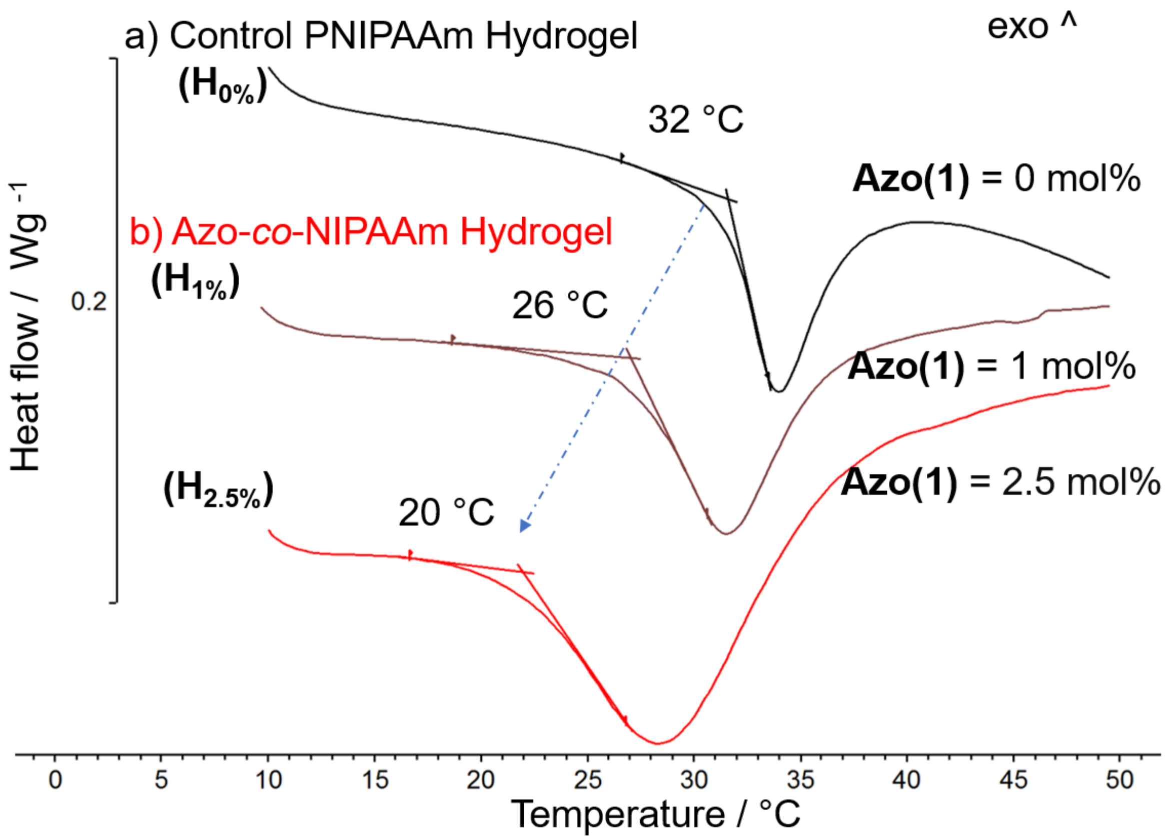

Figure S11. DSC scans (10 K/min) comparing the effect on the lower critical solution temperature (LCST) of the Control PNIPAAm polymer, $\mathbf{P_{0\%}}$ by the increase in the azobenzene content (1, 2.5 mol%) in the hydrogels from Azo-*co*-NIPAAm, $\mathbf{H_{1\%}}$ and $\mathbf{H_{2.5\%}}$ respectively.

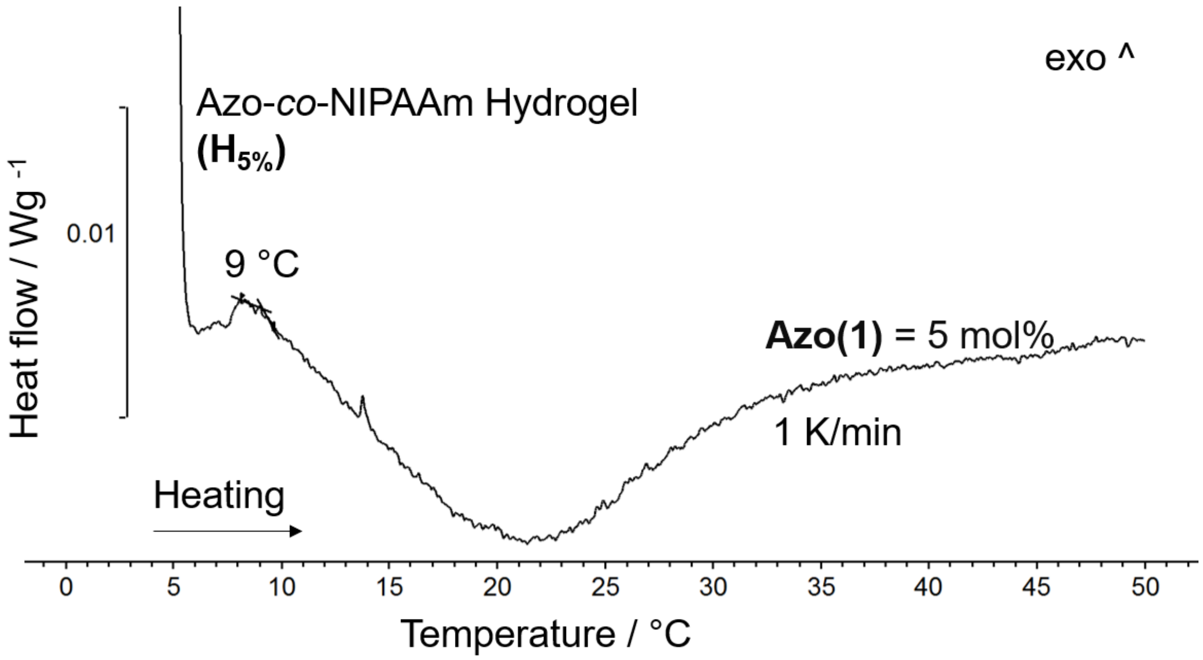

Figure S12. DSC scans (1 K/min) of Azo-*co*-NIPAAm hydrogel, $\mathbf{H_{5\%}}$ with **Azo(1)** content of 5 mol%.

11.6 Effect of the Thermal Cycle on the Partial (*Z*) in Non-treated Hydrogel ($\mathbf{H_{2.5\%}}$).

The first heating cycle in the DSC show for the functional hydrogel, $\mathbf{H_{2.5\%}}$, shows a feature which was ascribed to the (*Z*) isomer (**Figure S13**).

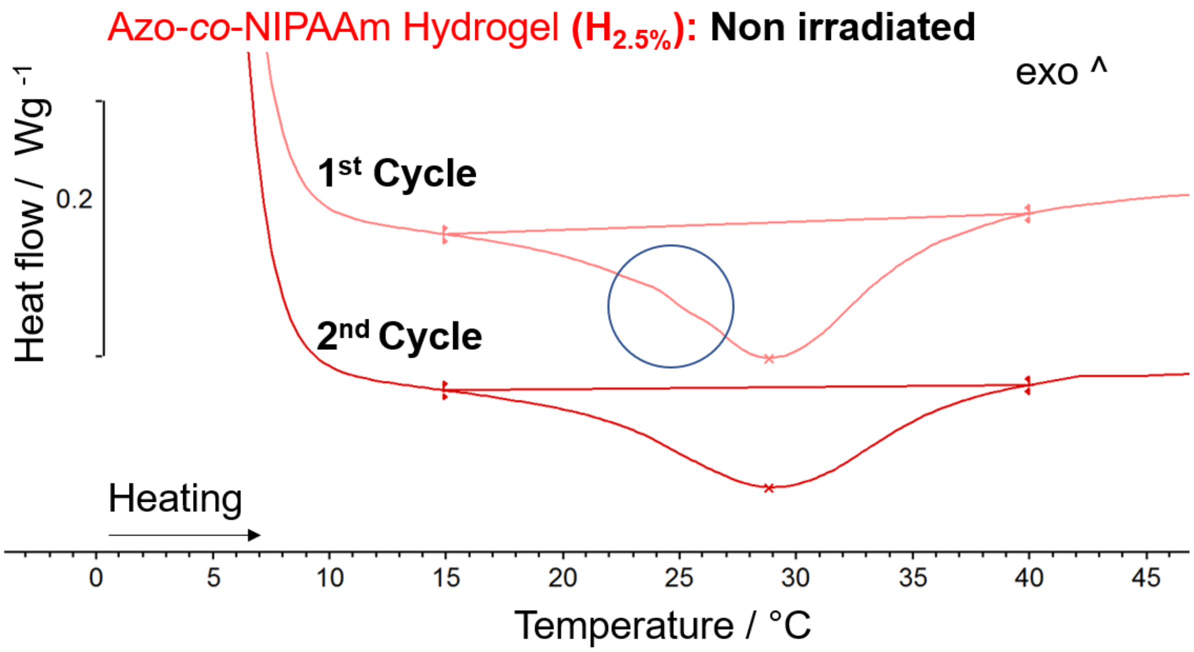

Figure S13. Effect of thermal reversal of the of (*Z*) character on the non-treated Azo-*co*-NIPAAm hydrogel, $\mathbf{H_{5\%}}$ in the DSC scans at (10 K/min) heating rate: 1st Cycle shows a slight shoulder peak which arises from the 32% (*Z*) character; 2nd Cycle represents a smooth endotherm reversing the effect due to the previous heating segment.

11.7 Effect of the Irradiation Time to Induce the Reversible LCST Shift

The influence of the increase in the UV irradiation time on the onset of the LCST of functional hydrogel, **H2.5%** is shown below (**Figure S14**).

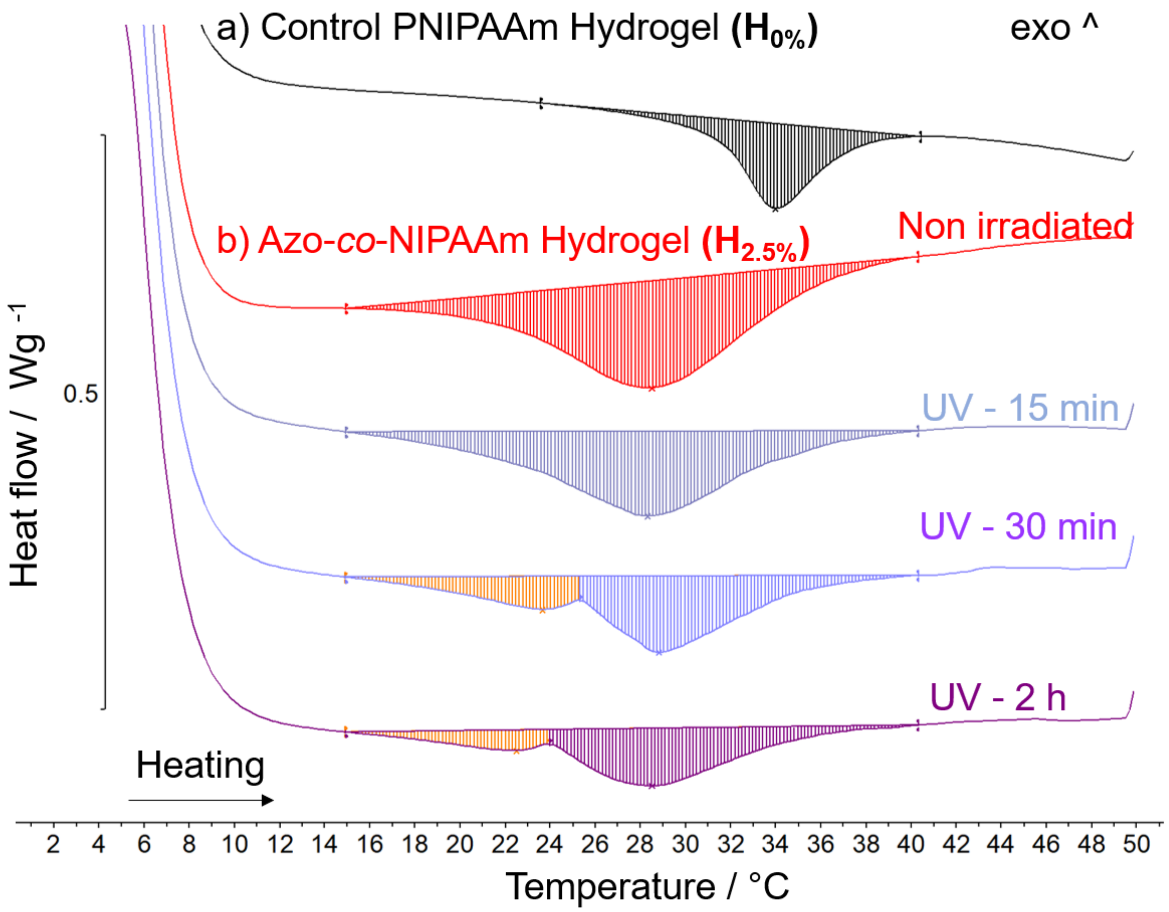

Figure S14. Effect of irradiation time UV (365 nm) on the onset of the lower critical solution temperature of Azo-*co*-NIPAAm hydrogel, **H2.5%**. DSC scans at (10 K/min) heating rate.

The influence of the increase in the visible light (450 nm) irradiation time on the onset of the LCST of functional hydrogel, **H**$_{2.5\%}$ is shown below (**Figure S15**).

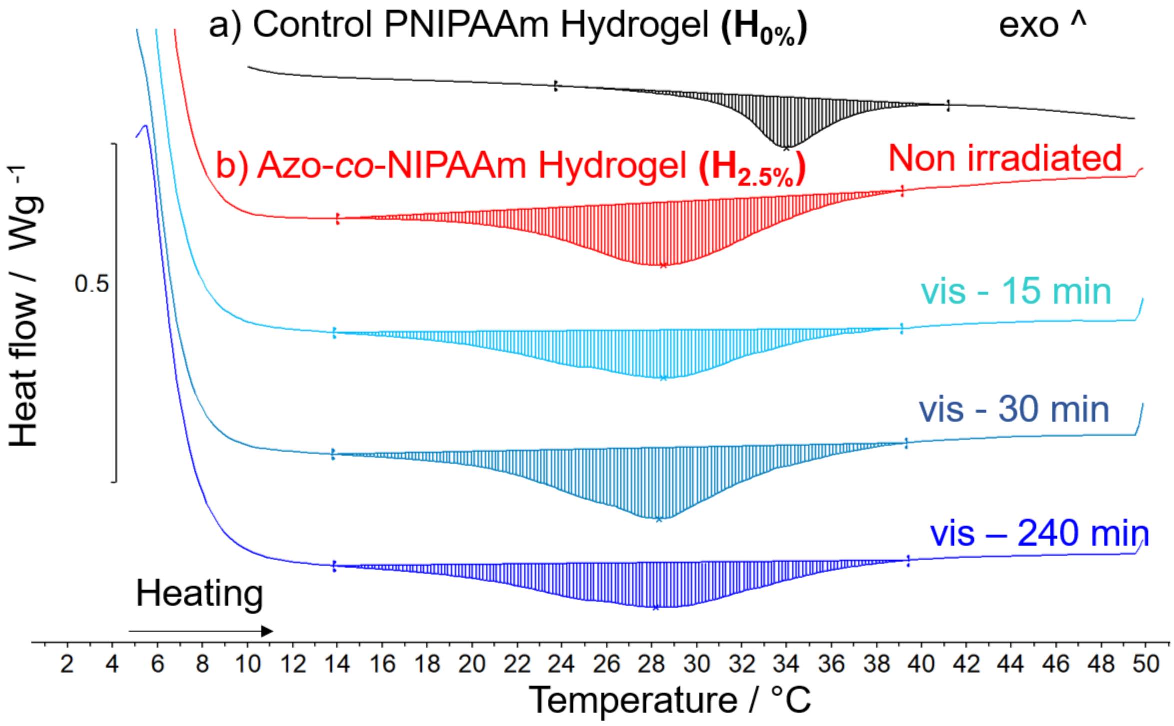

Figure S15. Effect of irradiation time of vis light (450 nm) on the onset of the lower critical solution temperature of Azo-*co*-NIPAAm hydrogel, **H**$_{2.5\%}$. DSC scans at (10 K/min) heating rate.

11.8 Effect of the Thermal Cycles on the UV Light-induced LCST Shift in $H_{2.5\%}$.

The LCST shift induced due to UV irradiation can be thermally reversed due to heat. This effect can be seen in the 2nd and the 3rd heat cycles during the DSC measurement (**Figure S16**).

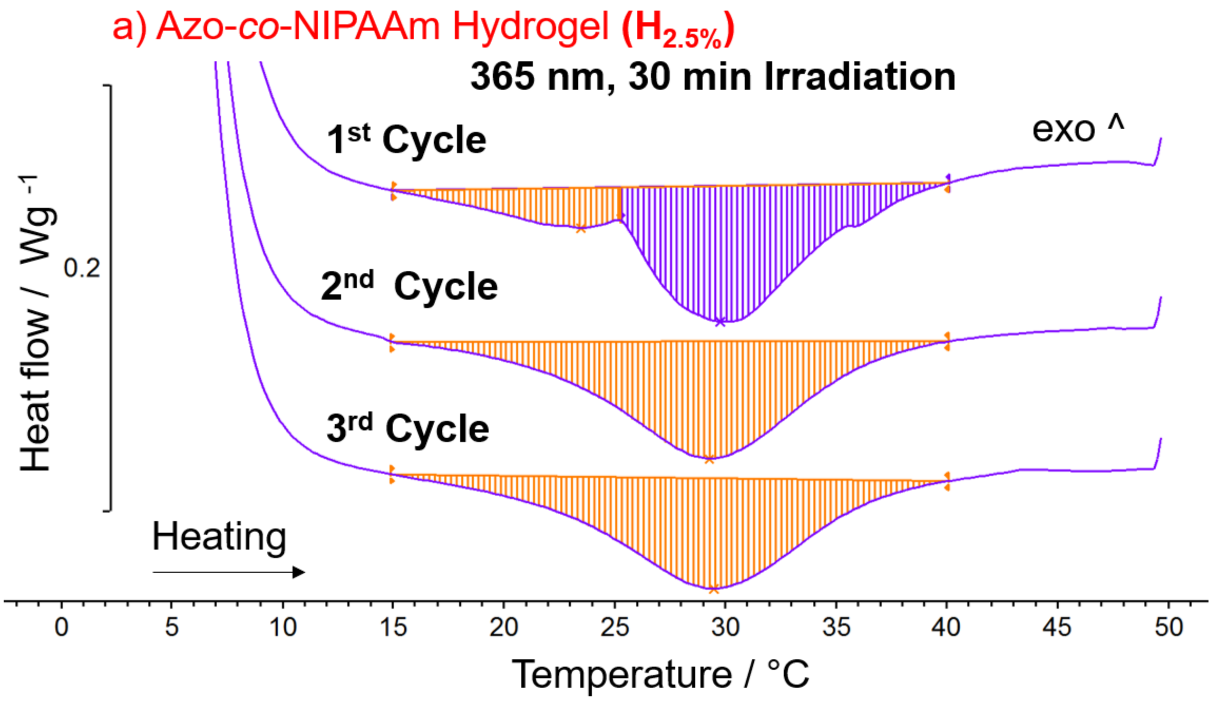

Figure S16. DSC scans (10 K/min) showing three consecutive cycles on UV (365 nm, 30 min) irradiated Azo-*co*-NIPAAm hydrogel, $\mathbf{H_{2.5\%}}$ indicates the heat induced reversibility on the LCST.

11.9 Effect of UV and Vis Irradiation on the LCST of $H_{1\%}$ and $H_{5\%}$ Hydrogels

The reversible LCST shift of $\mathbf{H_{1\%}}$ hydrogel on UV and vis light irradiation is shown in (**Figure S17**).

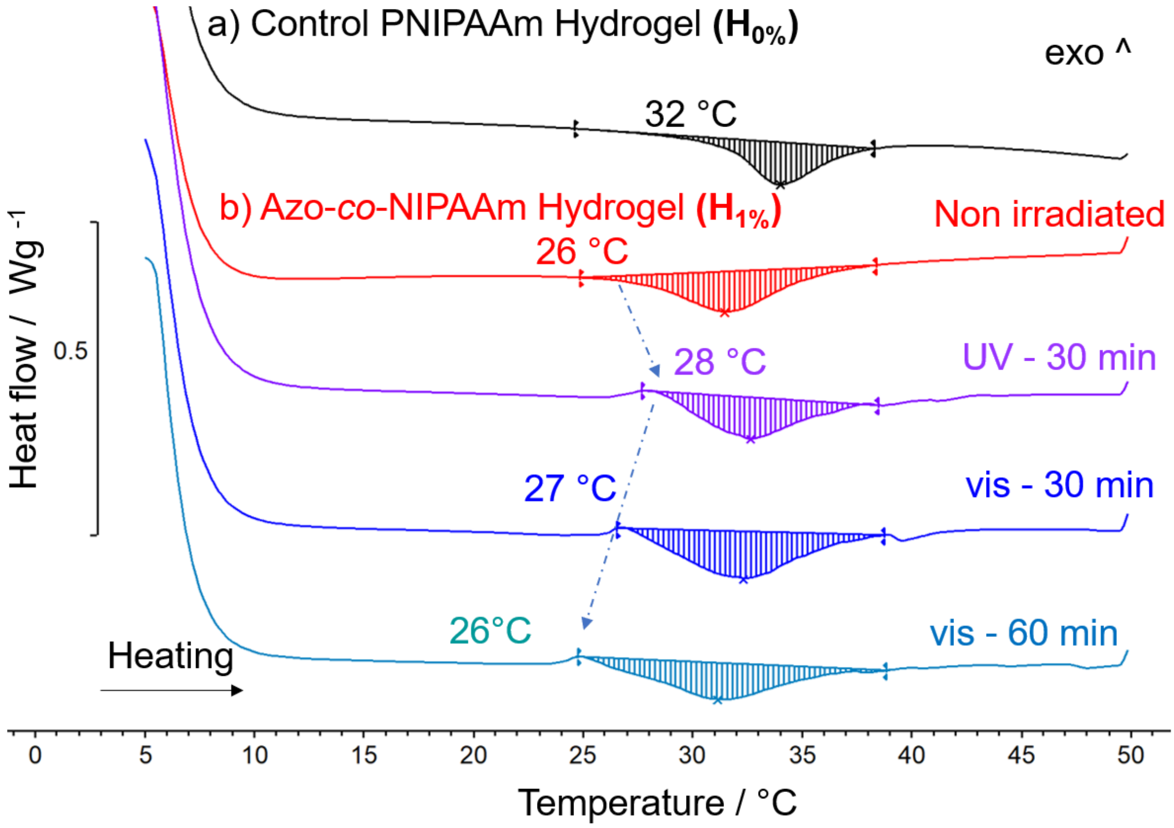

Figure S17. DSC scans (10 K / min) comparing the lower critical solution temperature (LCST) of the Control PNIPAAm ($\mathbf{H_{0\%}}$) to the azobenzene (1 mol%) functionalized, Azobenzene-*co*-PNIPAAm hydrogels ($\mathbf{H_{1\%}}$) hydrogels. a) The onset of the LCST of $\mathbf{H_{0\%}}$ in agreement with the literature range between 31 °C for pure PNIPAAm based systems. b) red: A 5 °C lowering of the LCST in the non-treated $\mathbf{H_{1\%}}$ hydrogel relative to $\mathbf{H_{0\%}}$, indicates the effect of incorporating hydrophobic azobenzene in PNIPAAm; purple: A LCST shift in the photochromic $\mathbf{H_{1\%}}$ hydrogel with a new endothermic transition temperature, LCST*$_{H1\%-(Z)}$ = 28°C on irradiation UV light (λ= 365 nm, 30 min); blue: partial reversing of the LCST by irradiation with blue light (λ= 450 nm, 30 min); light blue: full reversal of the LCST$_{H1\%-(E)}$ = 26 °C.

The effect of UV and vis light irradiation on **H$_{5\%}$** hydrogel on is shown in (**Figure S18**).

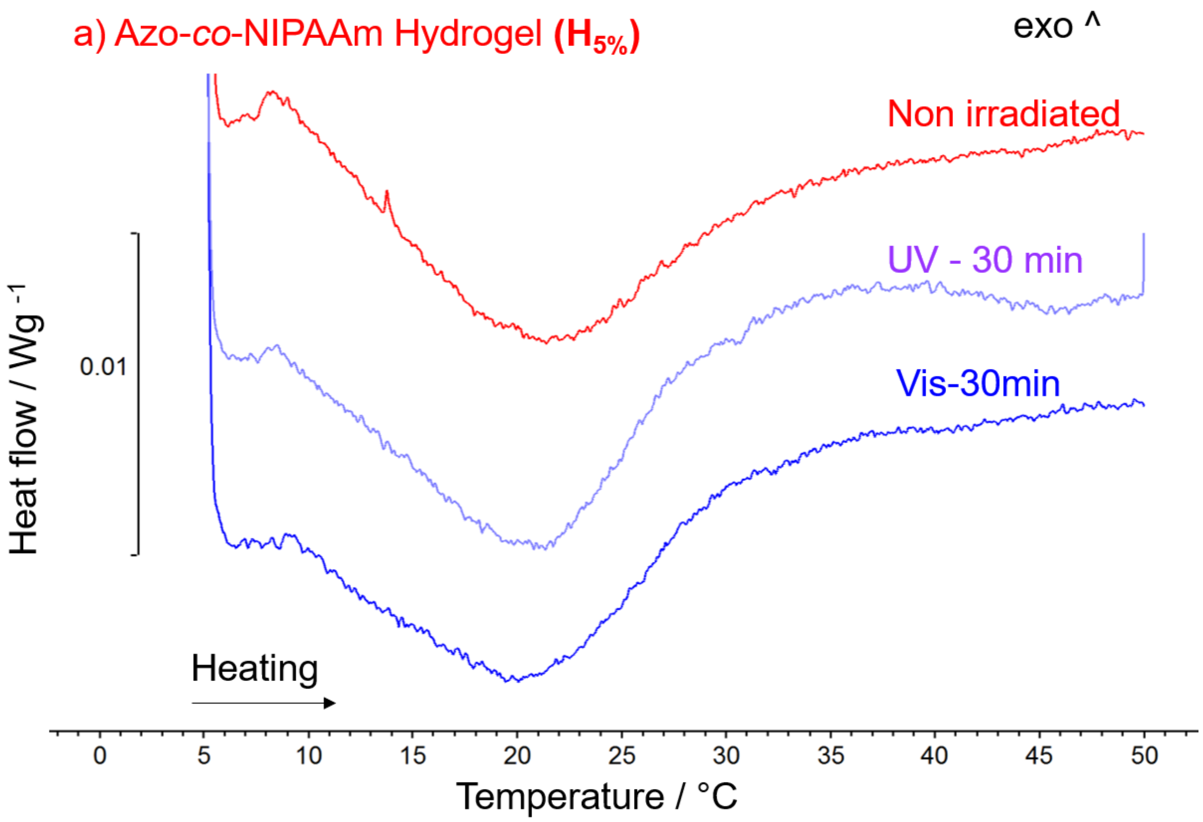

Figure S18. DSC scans (1 K/min) of Azo-*co*-NIPAAm hydrogel, **H$_{5\%}$** with **Azo(1)** content of 5 mol%; red: non-irradiated; purple: UV (365 nm, 30 min); blue: visible (450 nm, 30 min).

Table S13. LCST shift in hydrogels via DSC in the control PNIPAAm (H$_{0\%}$) and functionlized H$_{1\%}$ and H$_{2.5\%}$ hydrogels in non-treated and UV-switched state.

Hydrogels	S1	S2	S3	S4	Mean ± Standard deviation
**H$_{0\%}$**	31.5	31.8	31.5	31.8	31.6 ± 0.1
**H$_{1\%\text{-nontreeated}}$**	25.9	26.4	27.2	26.2	26.5 ± 0.6
**H$_{1\%\text{-365nm-30min}}$**	27.5	27.5	28.4	27.7	27.8 ± 0.6
**H$_{2.5\%\text{-non-treated}}$**	20.5	20.4	20.3	20.1	20.3 ± 0.1
**H$_{2.5\%\text{-365nm-30min}}$**	26.7	26.8	26	25.5	26.2 ± 0.6

12. Gel Permeation Chromatography of the Synthesized PNIPAAm and Azo-*co*-PNIPAAm Polymers

The samples for GPC were prepared in THF (1 mg/mL) and were run on a 3-set column to investigate the characteristics such as number average (**M_n**) and weight average (**M_w**) molecular weight (**Figure S19**) and calculate the corresponding poly dispersity index (PDI), see (**Table S14**). The total time for each measurement was 45 min with a 3-set column and the polymers eluted between 21 to 32 mL.

Table S14. Polymer characteristics obtained via GPC.

**Entry**	**Polymer**	**M_n**	**M_w**	**PDI = M_w/M_n**
1	**$P_{0\%}$**	12 kDa	20 kDa	1.6
2	**$P_{1\%}$**	15 kDa	27 kDa	1.8
3	**$P_{2.5\%}$**	17 kDa	32 kDa	1.9
4	**$P_{5\%}$**	12 kDa	28 kDa	2.3

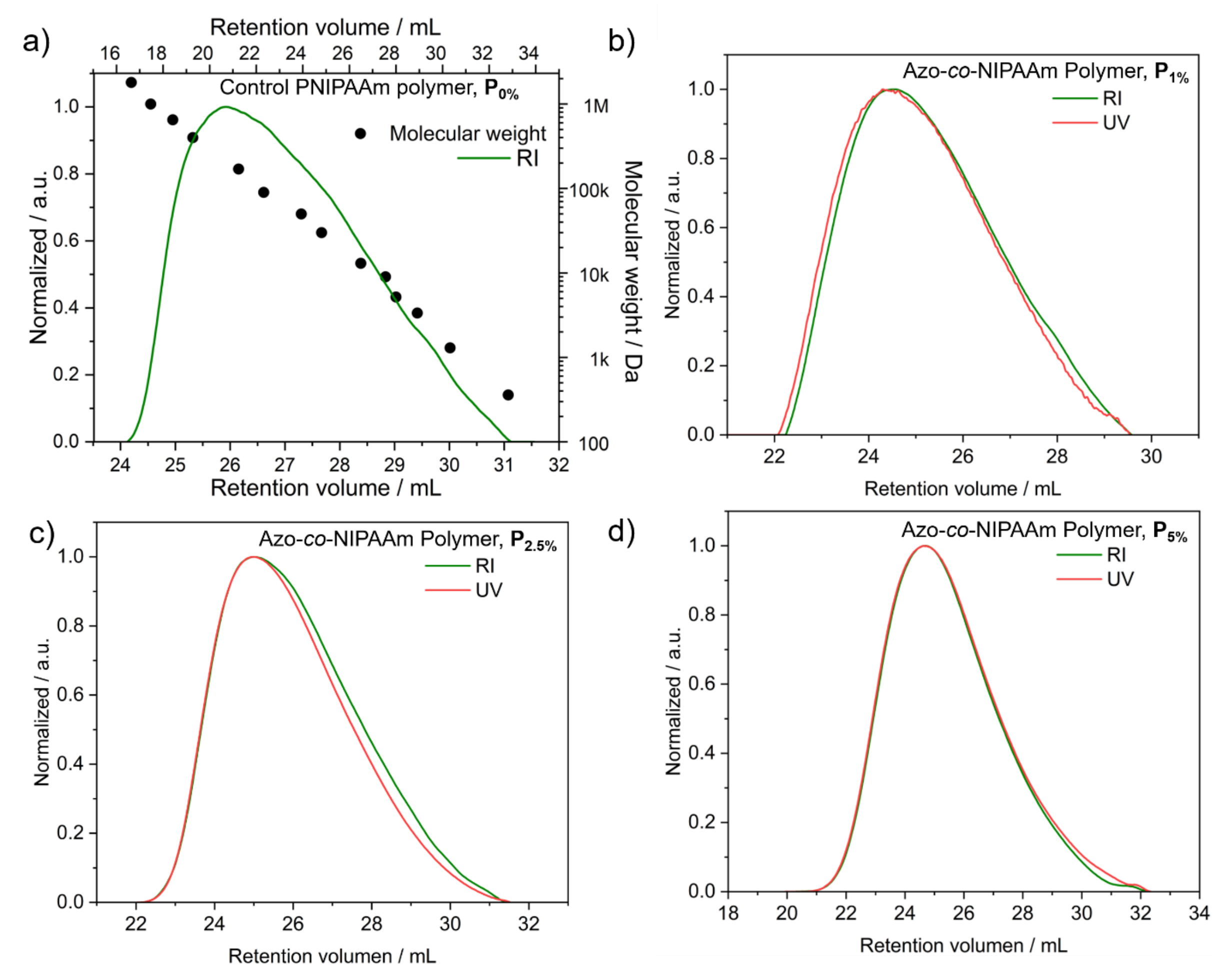

Figure S19. GPC plots with calibration of PS polymer standards (380 to 1.8 million) Da, the normalized intensity of RI (green) and UV (red) detector signal vs the retention volume/mL of a) Control PNIPAAm polymer, $\mathbf{P_{0\%}}$ and Azo-*co*-PNIPAAm polymers with varying azo content; b) $\mathbf{P_{1\%}}$; c) $\mathbf{P_{2.5\%}}$; d) $\mathbf{P_{5\%}}$.

In order to confirm the attachment of the photochromic moiety into the polymer chain, the GPC elugrams were recorded by setting the UV absorbance detector at 340 nm. The comparison of the functional polymers with the control PNIPAAm is shown (**Figure S20**). With a two-set column, the total measurement time was reduced to 30 min, therefore the elution time of the polymer was in the range of 12 to 20 mL.

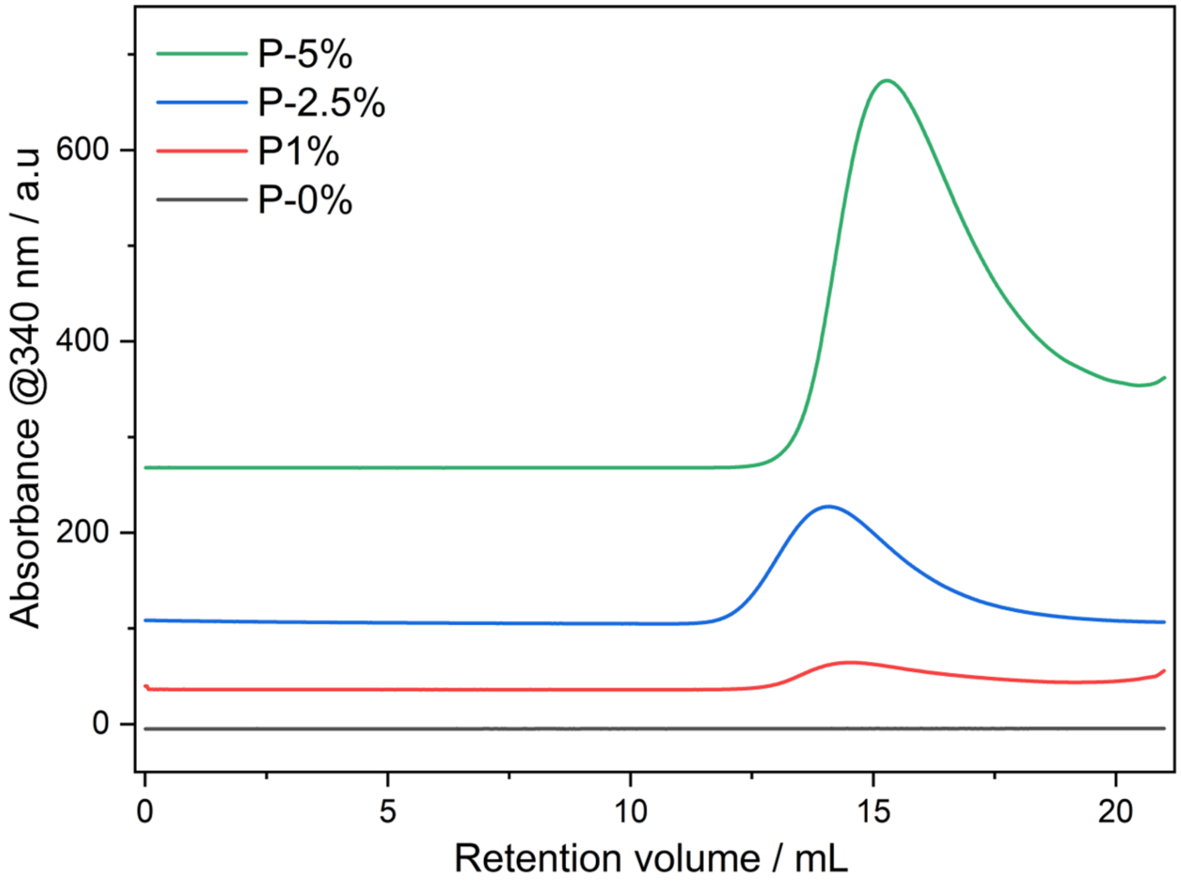

Figure S20. GPC elugram with UV detector set at 340 nm with the PNIPAAm polymer as the negative control.

13. ^{1}H NMR and NMR Spectra of the Synthesized Compounds

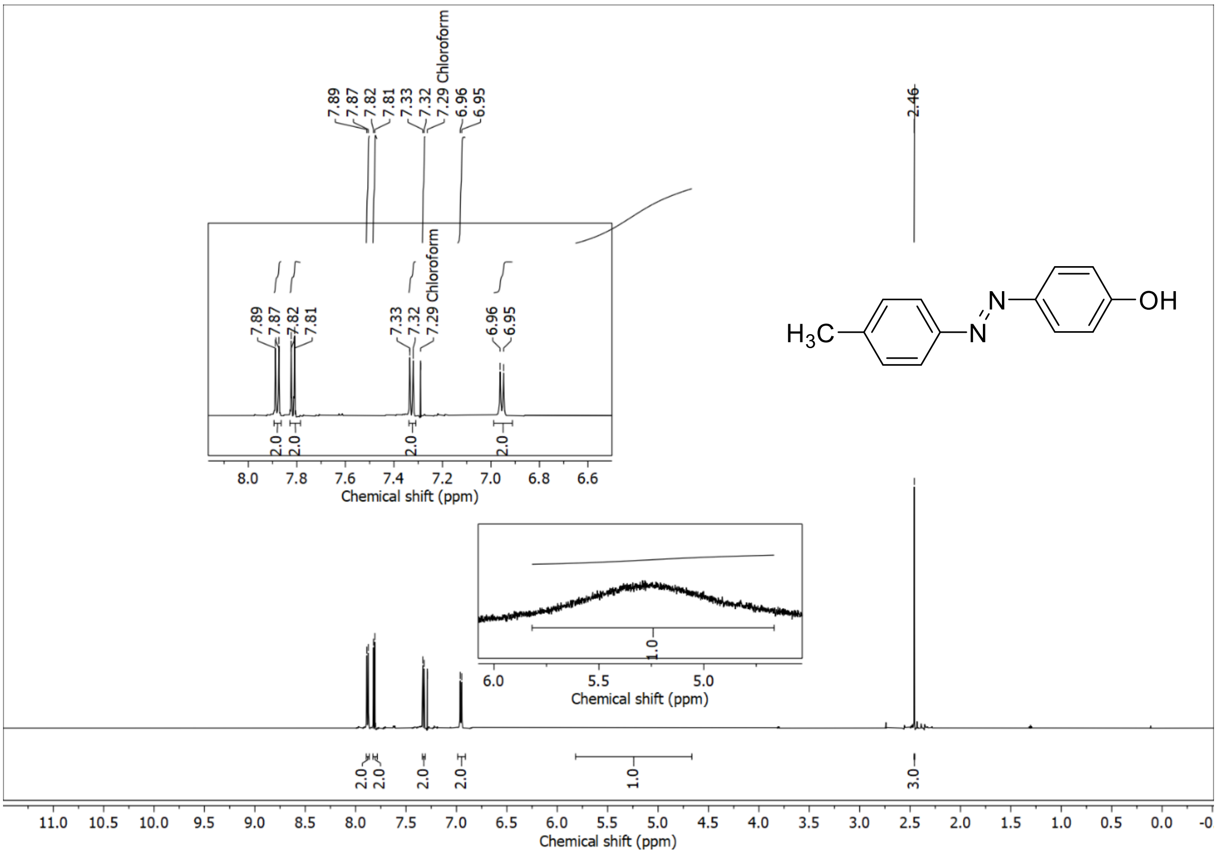

Figure S21. ^{1}H NMR spectrum of (*E*)-4-(*p*-tolyldiazenyl)phenyl acrylate **(s1)** in $CDCl_3$.

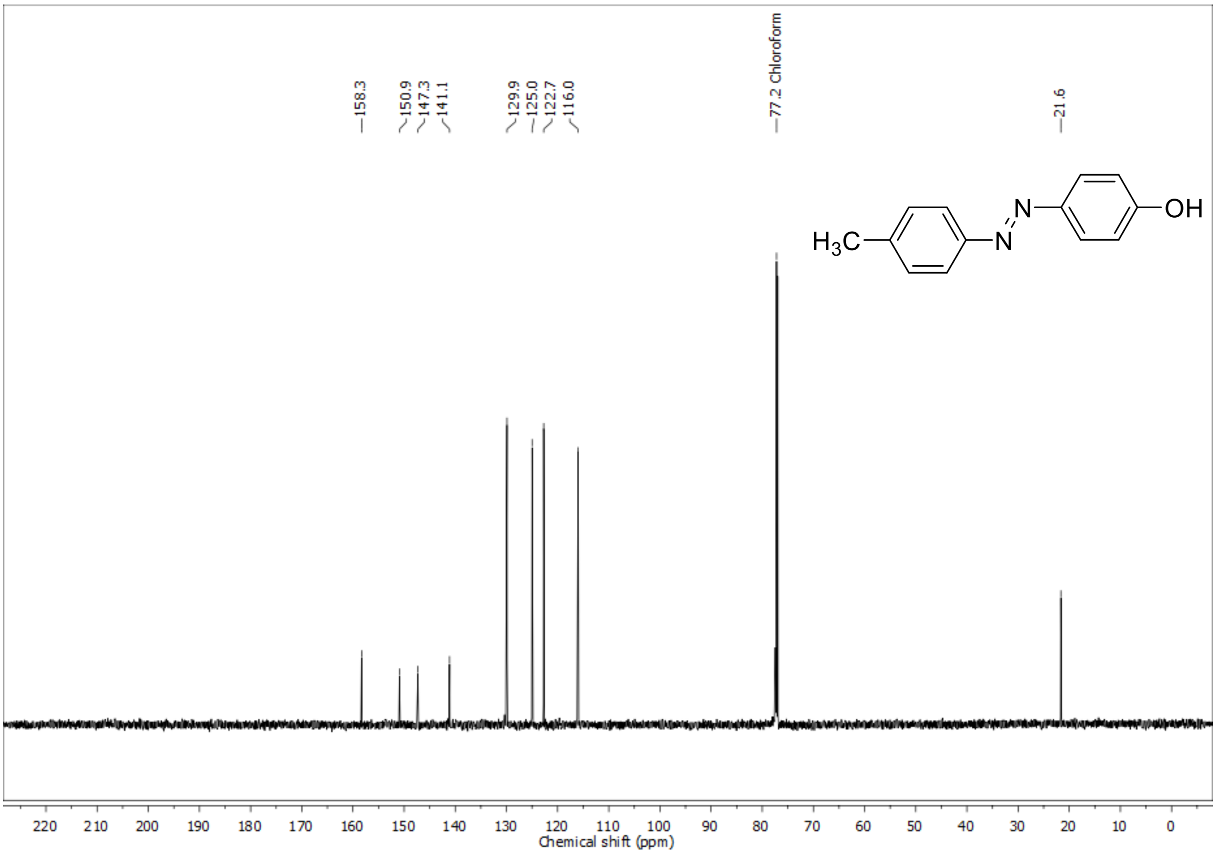

Figure S22. ^{13}C{H} NMR spectrum of (*E*)-4-(*p*-tolyldiazenyl)phenyl acrylate **(s1)** in $CDCl_3$.

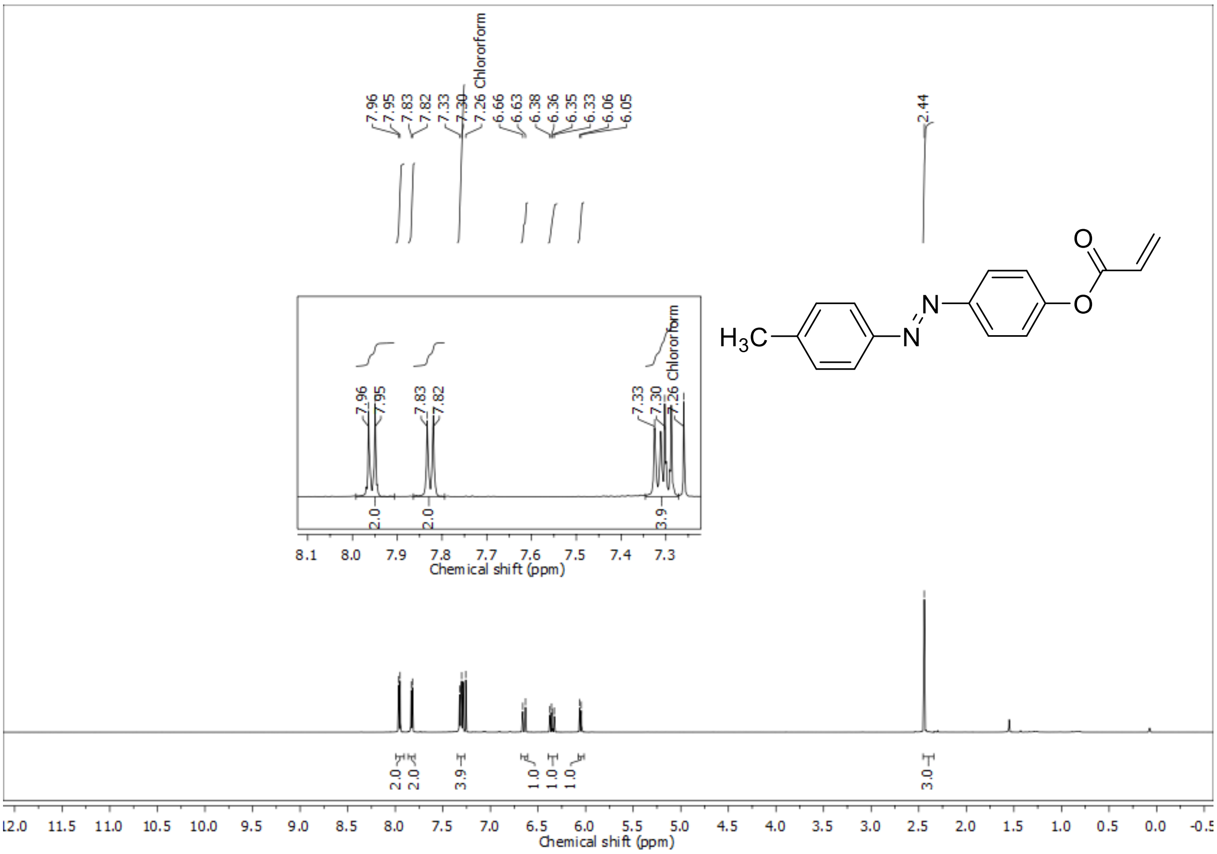

Figure S23.^{13}C{H} NMR spectrum of (*E*)-4-(*p*-tolyldiazenyl)phenyl acrylate, **Azo(1)** in $CDCl_3$.

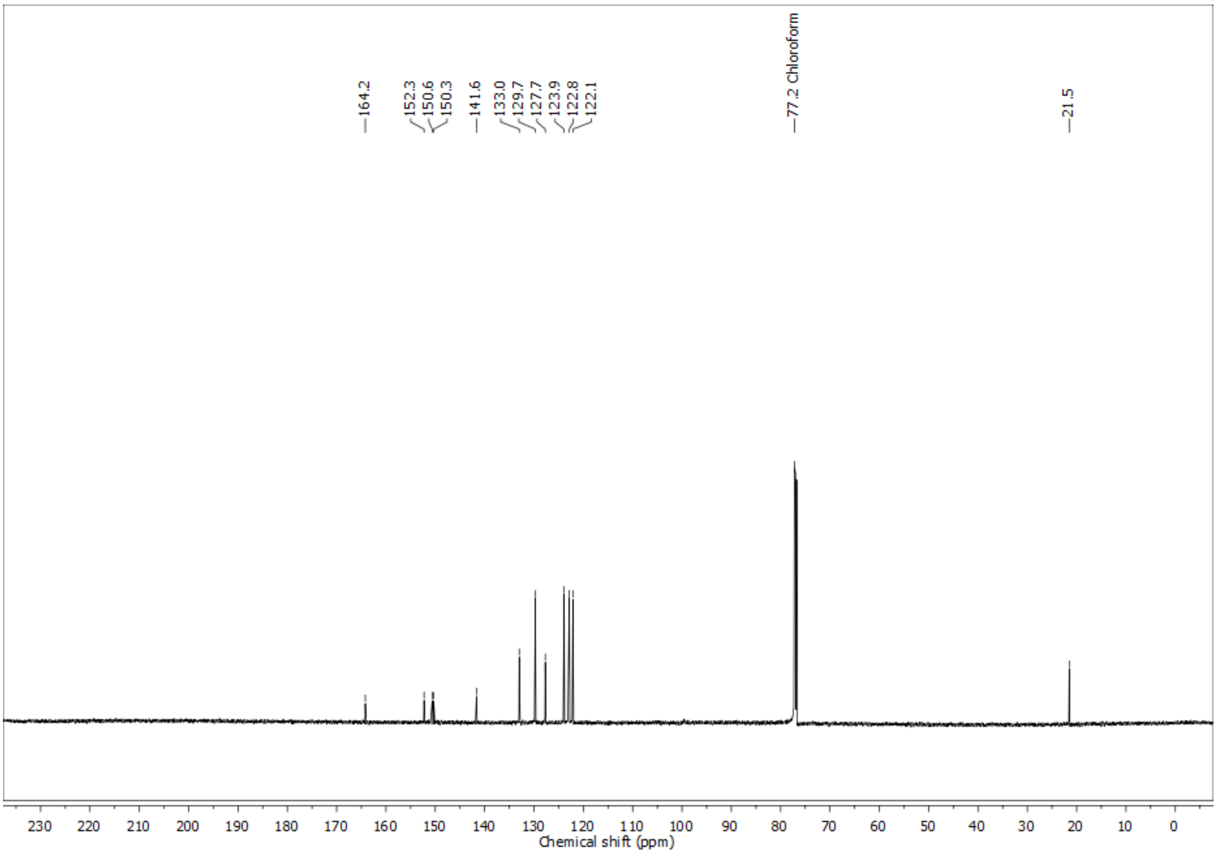

Figure S24. ^{1}H NMR spectrum of (*E*)-4-(p-tolyldiazenyl)phenyl acrylate, **Azo(1)** in $CDCl_3$.

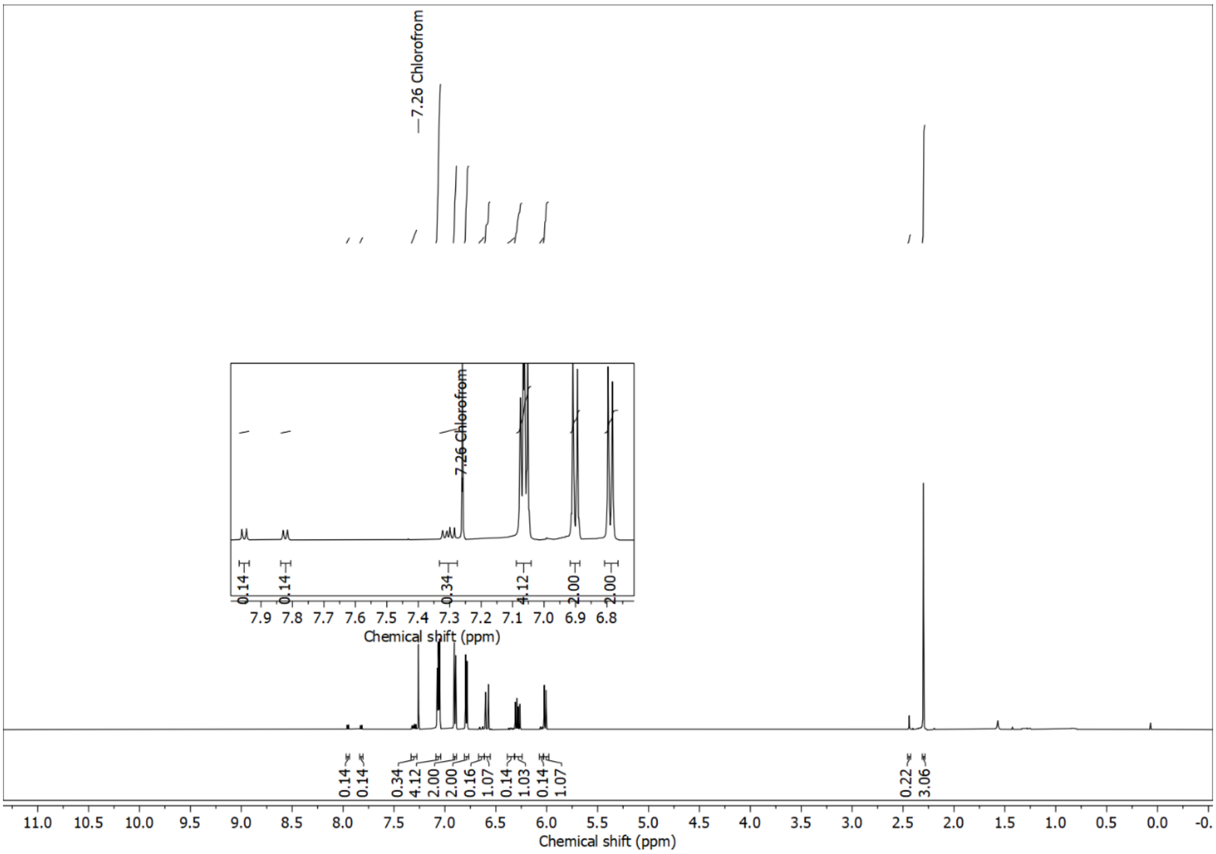

Figure S25. ^{1}H NMR spectrum of **Azo(1)** at PSS (365nm, 10 min) in $CDCl_3$.

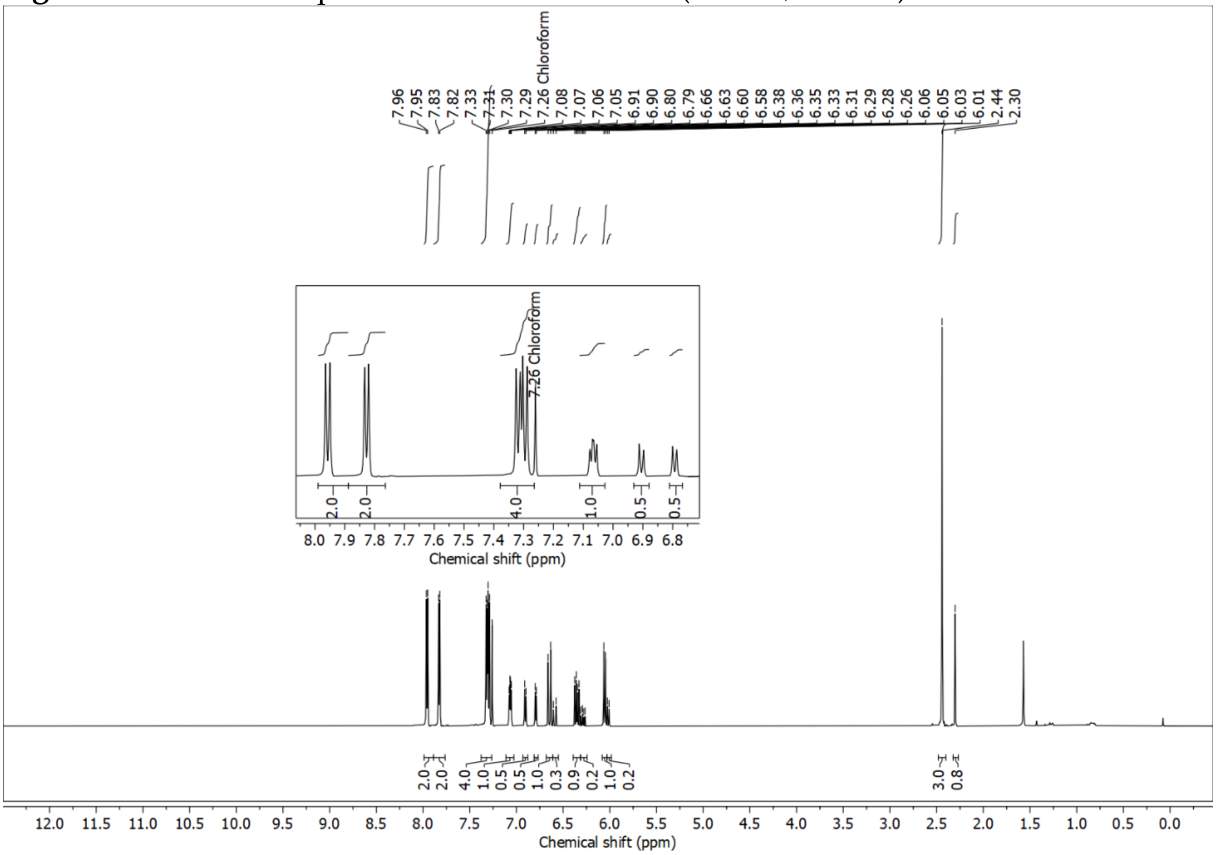

Figure S26. ^{1}H NMR spectrum of **Azo(1)** at PSS (450 nm, 30 min) in $CDCl_3$.

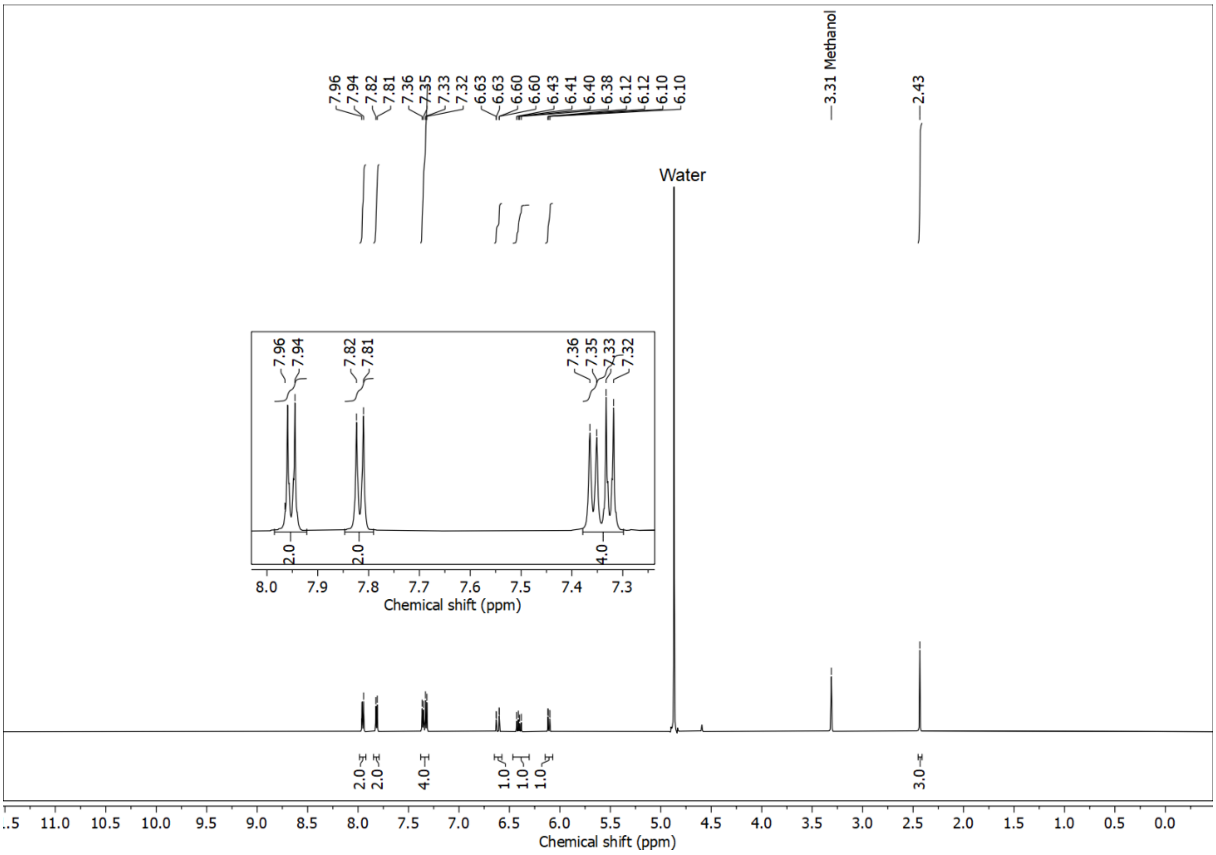

Figure S27. ^{1}H NMR spectrum of **Azo(1)** in MeOD.

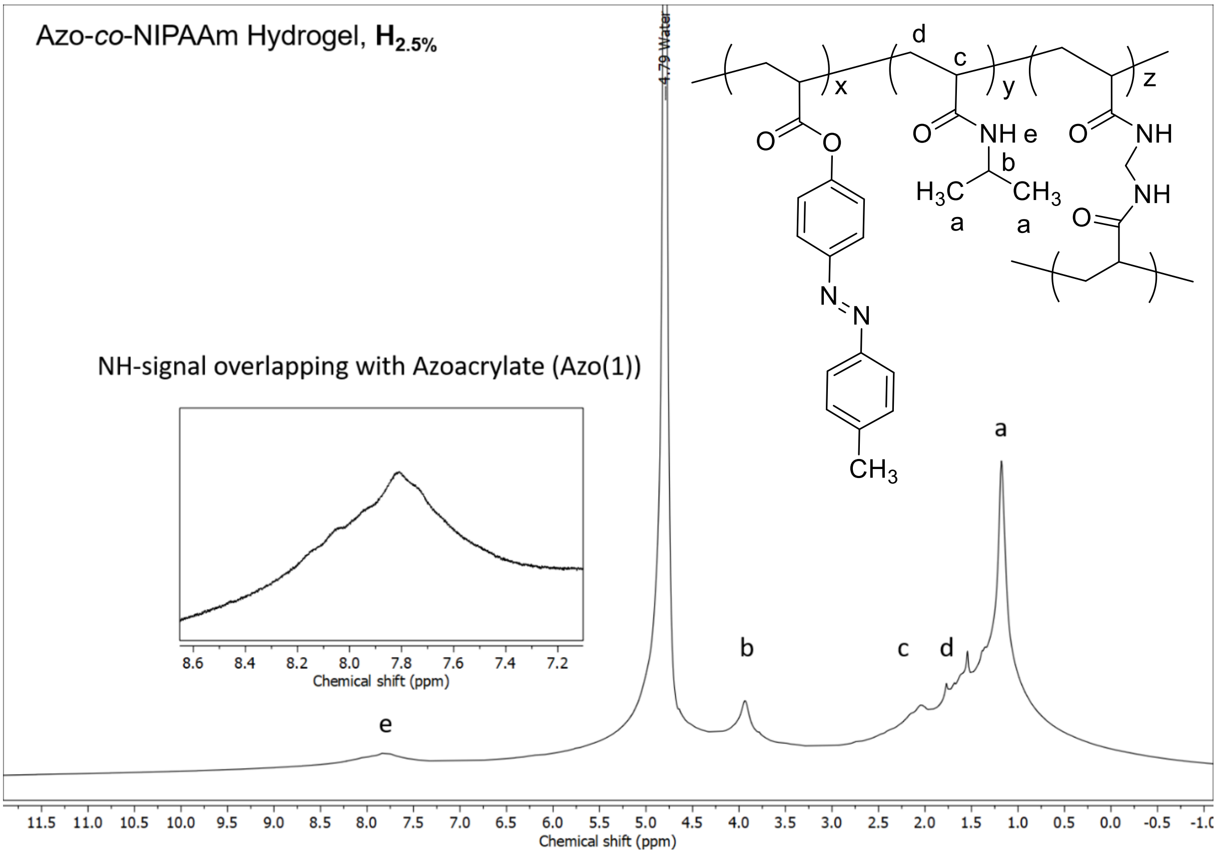

Figure S28. ^{1}H NMR spectrum of Azo-co-NIPAAm Hydrogel, **H$_{2.5\%}$** in D_2O.

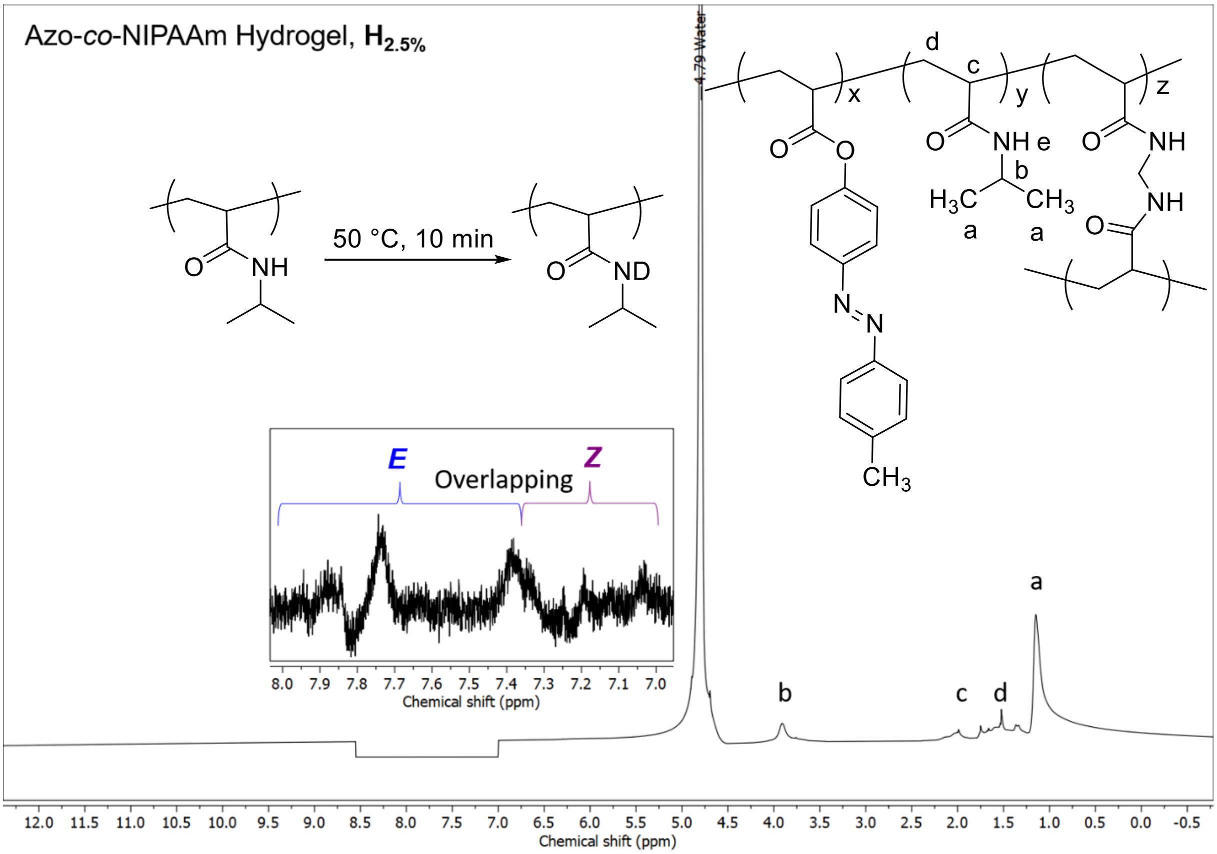

Figure S29. ^{1}H NMR spectrum of the Azo-*co*-NIPAAm Hydrogel, **H$_{2.5\%}$** in D_2O, pre-heated to 50 °C to allow deuterium exchange and visualize the azoacrylate, **Azo(1)** signals.

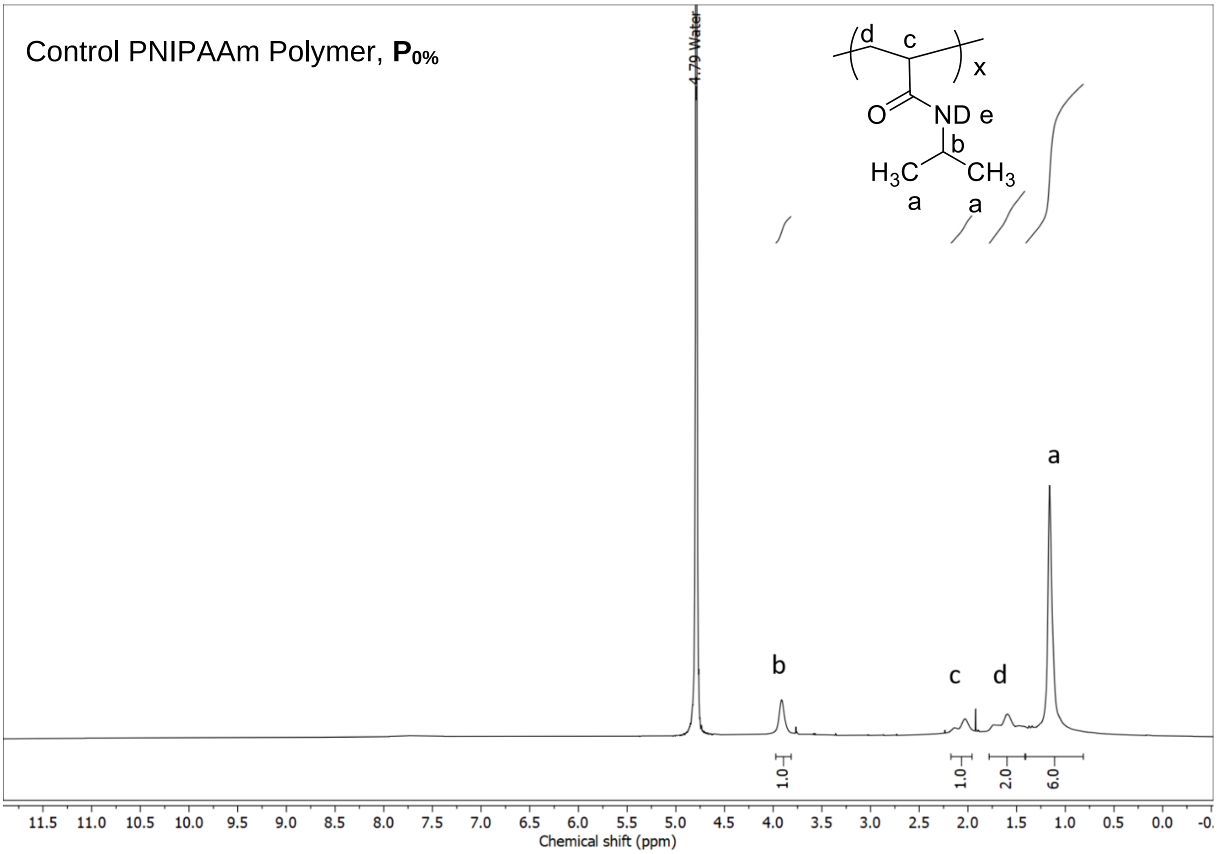

Figure S30.^{1}H NMR spectrum of the control PNIPAAm polymer, **P$_{0\%}$** in D_2O

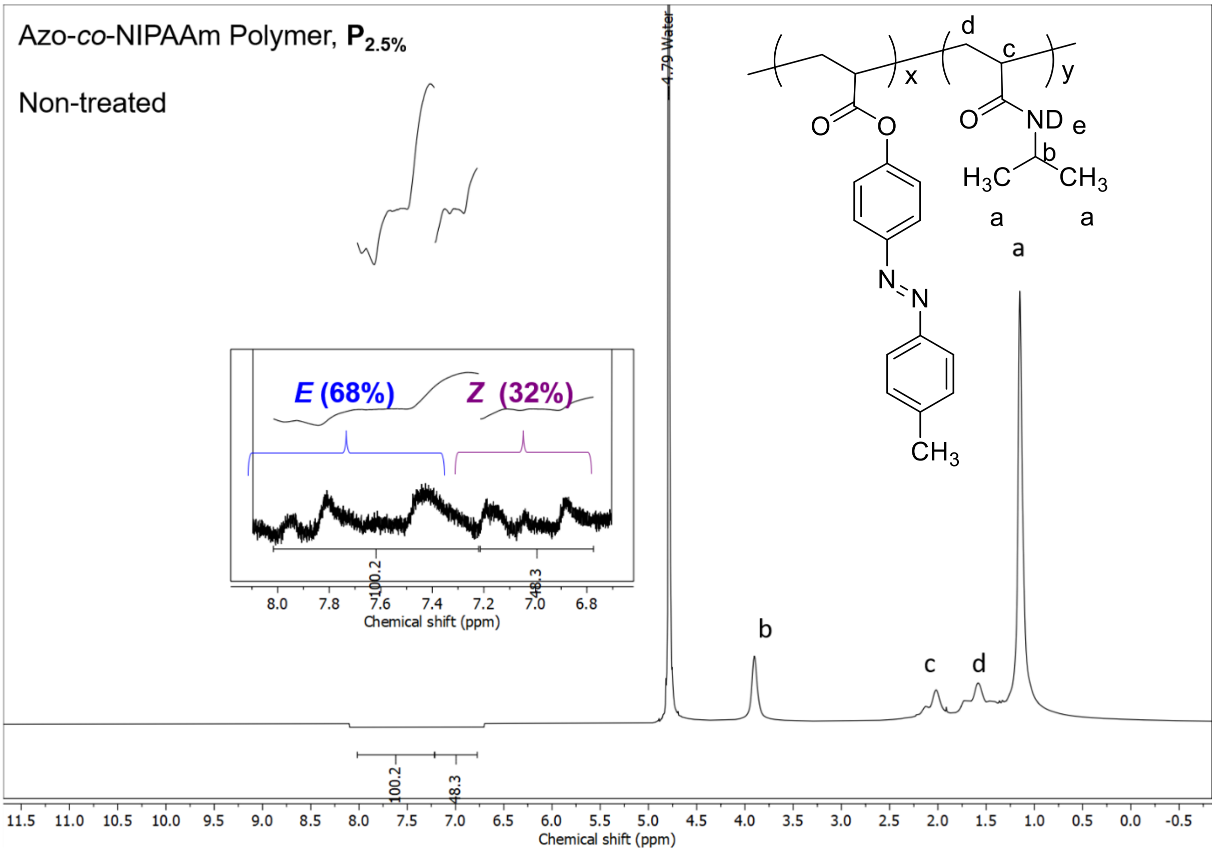

Figure S31. ^{1}H NMR spectrum of non-irradiated Azo-co-NIPAAm Polymer, **P$_{2.5\%}$** in D_2O.

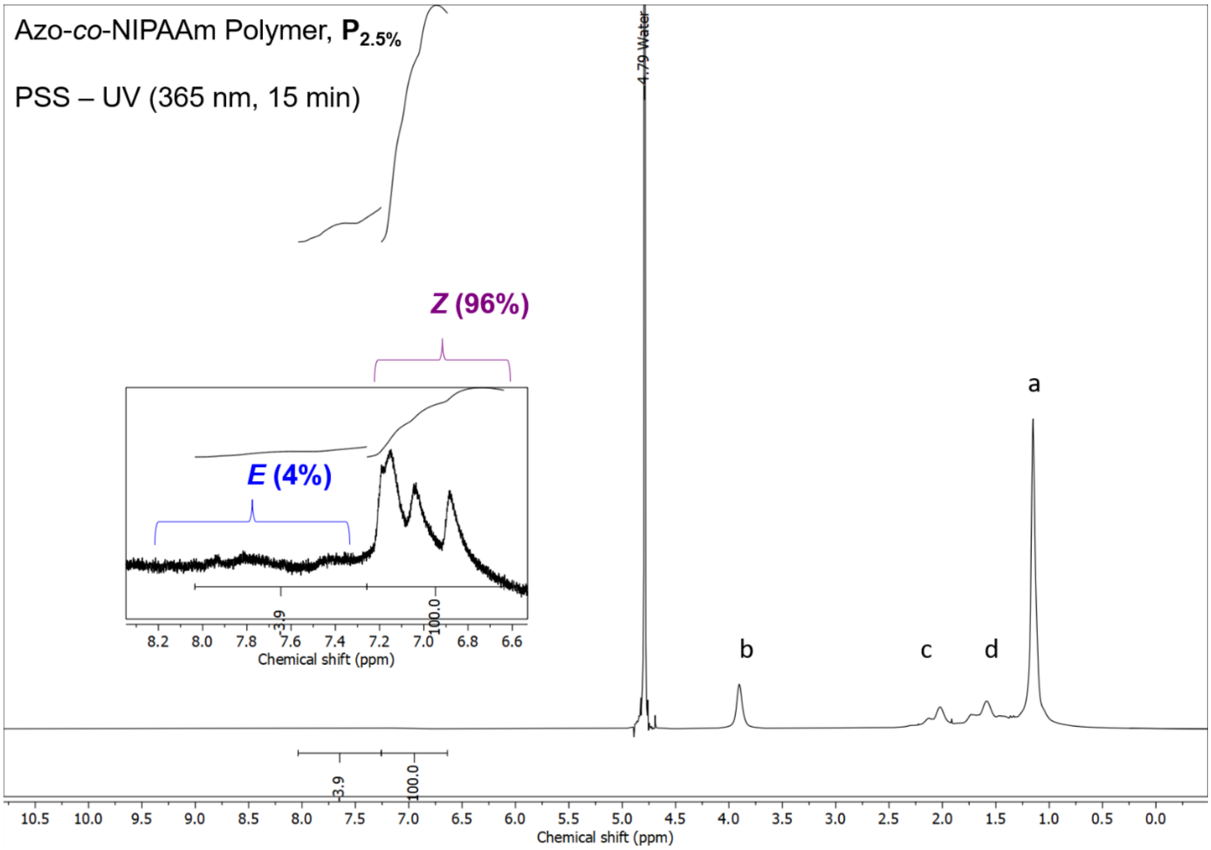

Figure S32. ^{1}H NMR spectrum of Azo-*co*-NIPAAm Polymer, $\mathbf{P}_{2.5\%}$ in D_2O at PSS (365 nm, 15 min) in D_2O.

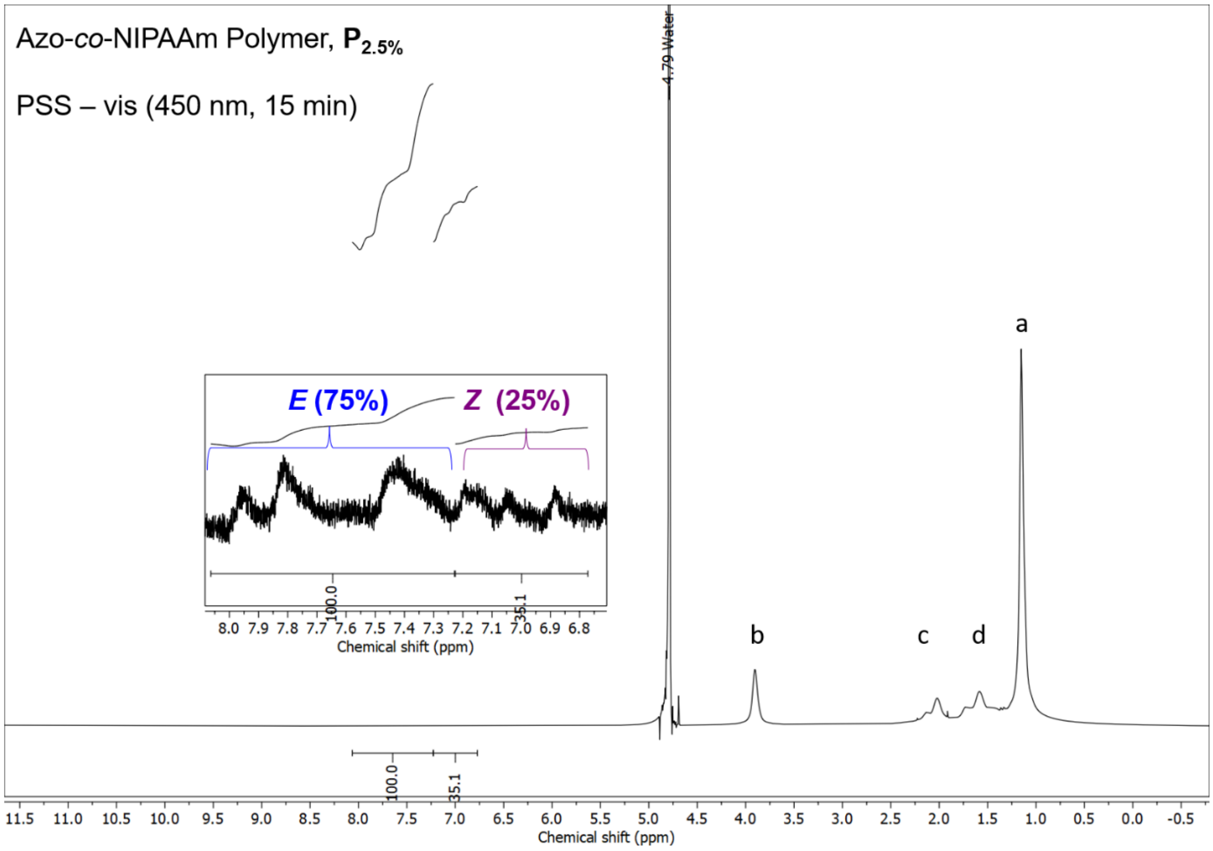

Figure S33. ^{1}H NMR spectrum of Azo-co-NIPAAm Polymer, $\mathbf{P}_{2.5\%}$ at PSS (450 nm, 15 min) in D_2O.

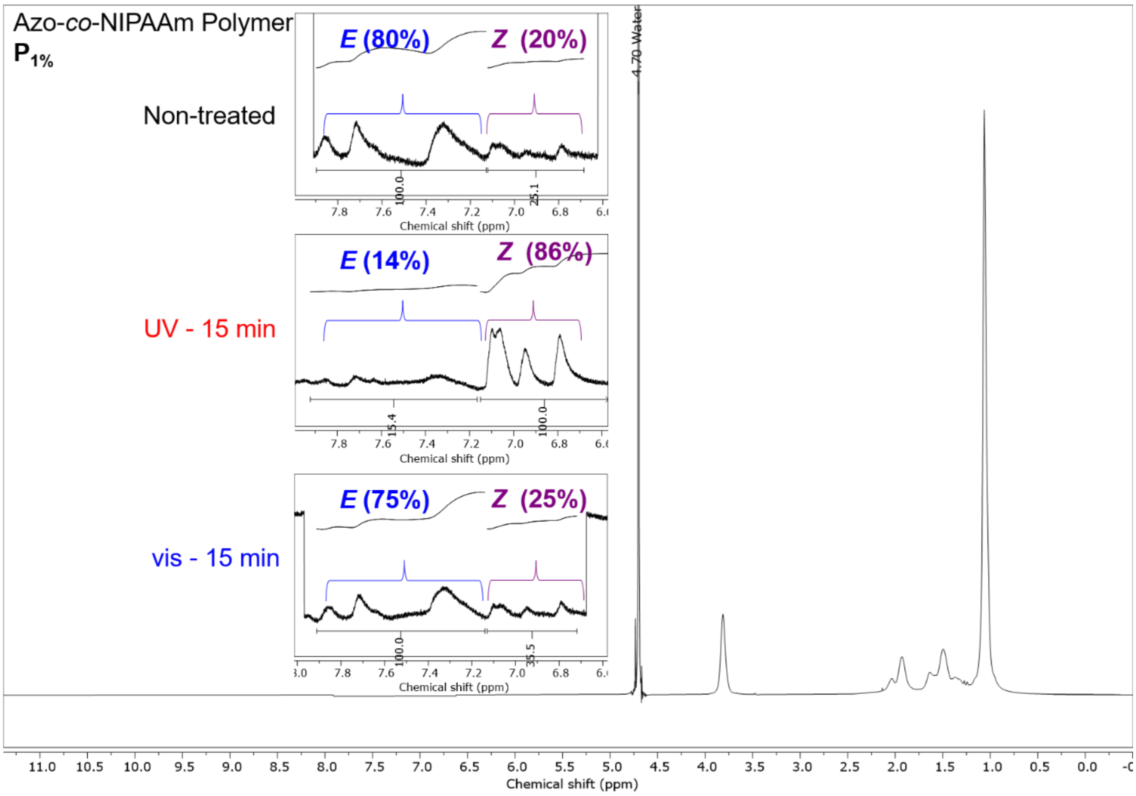

Figure S34. ^{1}H NMR spectrum of Azo-co-NIPAAm polymer, **P1%** in D_2O with magnified regions of **Azo(1)** aromatic signals under following conditions: top-nontreated; center: UV (365 nm, 15 min); bottom: visible (450 nm, 15 min).

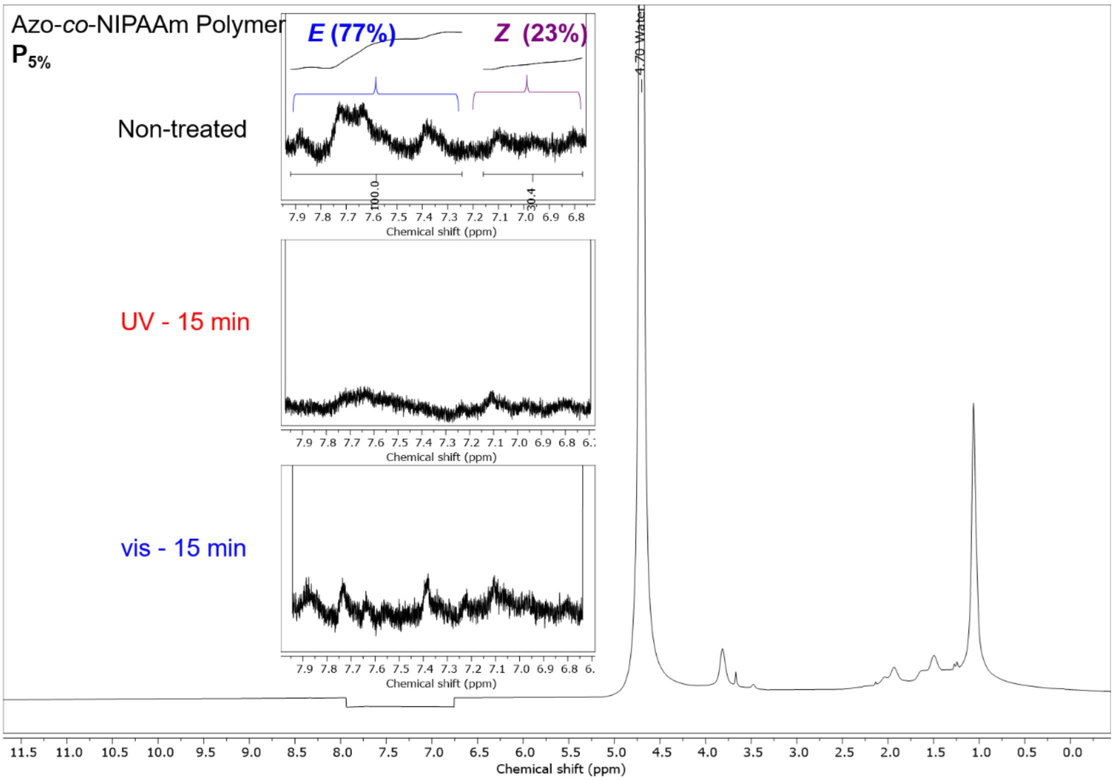

Figure S35. ^{1}H NMR spectrum of Azo-*co*-NIPAAm polymer, **P5%** in D_2O with magnified aromatic regions of **Azo(1)** under the following conditions: top-nontreated; overlapping and unresolvable signals at center: UV, 365 nm, 15 min and bottom: visible (450 nm, 15 min).

References

(1) Weis, P.; Hess, A.; Kircher, G.; Huang, S.; Auernhammer, G. K.; Koynov, K.; Butt, H. J.; Wu, S. Effects of Spacers on Photoinduced Reversible Solid-to-Liquid Transitions of Azobenzene-Containing Polymers. *Chemistry* **2019**, *25* (46), 10946-10953. DOI: 10.1002/chem.201902273.
